# Pooled analysis of epigenome-wide association studies of food consumption in KORA, TwinsUK and LLS

**DOI:** 10.1007/s00394-022-03074-9

**Published:** 2022-12-26

**Authors:** Fabian Hellbach, Lucy Sinke, Ricardo Costeira, Sebastian-Edgar Baumeister, Marian Beekman, Panayiotis Louca, Emily R. Leeming, Olatz Mompeo, Sarah Berry, Rory Wilson, Nina Wawro, Dennis Freuer, Hans Hauner, Annette Peters, Juliane Winkelmann, Wolfgang Koenig, Christa Meisinger, Melanie Waldenberger, Bastiaan T. Heijmans, P. Eline Slagboom, Jordana T. Bell, Jakob Linseisen

**Affiliations:** 1 Institute for Medical Information Processing, Biometry, and Epidemiology, Medical Faculty, Ludwig-Maximilian University Munich, Marchioninistr. 15, 81377 Munich, Germany; 2 Epidemiology, Faculty of Medicine, University of Augsburg, University Hospital Augsburg, Stenglinstraße 2, 86156 Augsburg, Germany; 3 Molecular Epidemiology, Department of Biomedical Data Sciences, Leiden University Medical Center, Einthovenweg 20, 2333 ZC Leiden, The Netherlands; 4 Department of Nutritional Sciences, King’s College London, London, UK; 5 Department of Twin Research and Genetic Epidemiology, King’s College London, London, SE1 7EH England, UK; 6 Institute of Health Services Research in Dentistry, Medical Faculty, University of Münster, Albert-Schweitzer-Campus 1, 48149 Münster, Germany; 7 Institute of Epidemiology, Helmholtz Zentrum München, German Research Center for Environmental Health (GmbH), Ingolstädter Landstr. 1, 85764 Neuherberg, Germany; 8 Research Unit Molecular Epidemiology, Helmholtz Zentrum München, German Research Center for Environmental Health (GmbH), Ingolstädter Landstr. 1, 85764 Neuherberg, Germany; 9 Else Kröner-Fresenius-Center for Nutritional Medicine, TUM School of Life Sciences, Technical University of Munich, 85354 Freising, Germany; 10 Institute of Nutritional Medicine, School of Medicine, Technical University of Munich, Georg-Brauchle-Ring 62, 80992 Munich, Germany; 11 German Center for Diabetes Research (DZD E.V.), Ingolstädter Landstr. 1, 85764 Munich-Neuherberg, Germany; 12 Institute of Neurogenomics, Helmholtz Zentrum München, German Research Center for Environmental Health (HmbH), Ingolstädter Landstr. 1, 85764 Neuherberg, Germany; 13 DZHK (German Centre for Cardiovascular Research), Partner Site Munich Heart Alliance, Pettenkoferstr. 8A & 9, 80336 Munich, Germany; 14 German Heart Centre Munich, Technical University Munich, Lazarettstr. 36, 80636 Munich, Germany; 15 Institute of Epidemiology and Medical Biometry, University of Ulm, Helmholtzstr. 22, 89081 Ulm, Germany

**Keywords:** Diet, EWAS, Food group, High-fat foods, Humans

## Abstract

**Purpose:**

Examining epigenetic patterns is a crucial step in identifying molecular changes of disease pathophysiology, with DNA methylation as the most accessible epigenetic measure. Diet is suggested to affect metabolism and health via epigenetic modifications. Thus, our aim was to explore the association between food consumption and DNA methylation.

**Methods:**

Epigenome-wide association studies were conducted in three cohorts: KORA FF4, TwinsUK, and Leiden Longevity Study, and 37 dietary exposures were evaluated. Food group definition was harmonized across the three cohorts. DNA methylation was measured using Infinium MethylationEPIC BeadChip in KORA and Infinium HumanMethylation450 BeadChip in the Leiden study and the TwinsUK study. Overall, data from 2293 middle-aged men and women were included. A fixed-effects meta-analysis pooled study-specific estimates. The significance threshold was set at 0.05 for false-discovery rate-adjusted p values per food group.

**Results:**

We identified significant associations between the methylation level of CpG sites and the consumption of onions and garlic (2), nuts and seeds (18), milk (1), cream (11), plant oils (4), butter (13), and alcoholic beverages (27). The signals targeted genes of metabolic health relevance, for example, *GLI1, RPTOR,* and *DIO1,* among others.

**Conclusion:**

This EWAS is unique with its focus on food groups that are part of a Western diet. Significant findings were mostly related to food groups with a high-fat content.

**Supplementary Information:**

The online version contains supplementary material available at 10.1007/s00394-022-03074-9.

## Introduction

Examining epigenetic modifications is a crucial step in exploring the effects of diet on human metabolism. Such modifications can occur at different biological levels, including DNA methylation, modification of histones and noncoding RNAs. The availability of precise measurement tools, the level of inter-individual variation and the expected effect sizes make DNA methylation the most appropriate research tool for diet and epigenetics studies [1].

DNA-methyl-transferase enzymes (DNMT) catalyze the generation of 5-methylcytosine, the main contributor of DNA methylation patterns, by utilizing methyl groups. Since 5-methylcytosine is degradable and insufficient activity of a maintenance DNMT enzyme can lead to loss of methylation with each cell division [2], there is a steady need for methyl group supply. Dietary intake represents the main source for methyl groups. Methionine, choline and its metabolite betaine [3], are all embedded in the C1 metabolism, contributing to the synthesis of the main methyl donor in human metabolism: s-adenosylmethionine. This makes the C1 metabolism the hypothesized primary link between diet and DNA methylation. However, research examining this link showed inconclusive results [4, 5], thus indicating that dietary methyl group donors and vitamins involved in the C1 metabolism are not major determinants for DNA methylation pattern changes. Analysis of food consumption data may better reflect synergistic effects of various food components as compared to single nutrients. Another link between diet and DNA methylation could be through modulation of inflammatory processes. Dietary compounds have been shown to be associated with systemic inflammation [6], which in turn can lead to disturbances in the balance of DNA methylation patterns [3].

So far, some analyses on the link between diet and global DNA methylation patterns [7], as well as diet and site-specific epigenetic changes [3], have been performed. In terms of site-specific analysis, the main focus of nutri-epigenomic research has been on epigenome-wide association studies (EWAS) of nutrients involved in human C1 metabolism [3, 4]. EWAS have also been carried out with dietary patterns and few single food groups [8–10]. However, a comprehensive EWAS at the food group level is lacking. Thus, our aim was to explore the association between food consumption and DNA methylation in population-based studies. We aimed to identify DNA methylation associations with food groups that (i) provide nutrients involved in the human C1 metabolism, (ii) are known in the literature for being associated with systemic inflammation (like red meat, cabbage or nuts), or (iii) were shown to be associated with cardio-metabolic disease risks (like sugar-sweetened beverages or vegetables) previously. The results of the EWAS conducted in three cohorts, KORA FF4 (KORA), TwinsUK (TUK) and Leiden Longevity Study (LLS), were included in this meta-analysis.

## Methods

The “Strengthening the Reporting of Observational Studies in Epidemiology—Nutritional Epidemiology (STROBE-nut)” checklist was used to report the findings of the present study [11]. For an overview of key points of methodology used in respective cohorts, see Table 1.

**Table 1:** Key points of methodology used in all three cohorts

	KORAFF4	LLS	TwinsUK
Dietary data	Usual dietary intake in g/day (methodology described as in [20]. Repeated 24 h food lists (246 items) and FFQ as adjusting variable	FFQ (218 items), calculated in g/day	FFQ (131 items), calculated in g/day
Methylation data	Infinium MethylationEPIC BeadCHip (~ 850 k loci); preprocessing with the package minfi	Illumina HumanMethylation450 array (~ 450 k loci); quality control using MethylAid	Illumina HumanMethylation450 array (~ 450 k loci); preprocessing with ENmix and minfi
Statistical model	Linear multivariable regression with technical covariates and food intake residual as exposure and methylation beta values as outcome	Linear multivariable regression with technical covariates and food intake residual as exposure and methylation beta values as outcome	Mixed-model with cohort-specific random-effects and food intake residual as exposure and methylation beta values as outcome

### Populations

The KORA (Cooperative Health Research in the Region of Augsburg) FF4 study is the second follow-up of the population-based KORA S4 examination. It was conducted between 1999 and 2001 in the city of Augsburg and two surrounding counties in Germany. 4261 subjects aged 25–74 years were randomly drawn and agreed to participate in the S4 baseline study. 2279 of them also participated in the FF4 follow-up study (2013/2014). Details regarding the recruitment procedure have been published elsewhere [12]. Methylation data was available for 1928 subjects, and after exclusion of outliers (as described in the DNA methylation section), 1888 subjects remained. Further we excluded cases without available nutrition data (*n* = 541) or with blood cancer (*n* = 4). All participants met the criteria of acceptable caloric intake (500 kcal/d < *x* < 5000 kcal/d). Finally, 1322 subjects had full information on all covariates and were included in the EWAS.

The LLS consists of 1671 members of long-lived families (mean age 60 years) and their 744 partners (mean age: 60 years) as population controls. Dietary intake data in grams per day was collected from 1716 individuals. Members of long-lived families are very similar to the general population, although they have more favorable glucose tolerance [13], more favorable lipid parameters [14], and a lower prevalence of type-2 diabetes and myocardial infarction [15]. We analyzed them as one cohort of middle-aged people, and the current study was restricted to unrelated individuals. EWAS data and nutritional data was available on 507 individuals. All LLS participants met the criteria of acceptable caloric intake (500 kcal/d < *x* < 5000 kcal/d). Finally, 485 subjects had full information on all covariates and therefore were included in the EWAS.

**Fig. 1:**
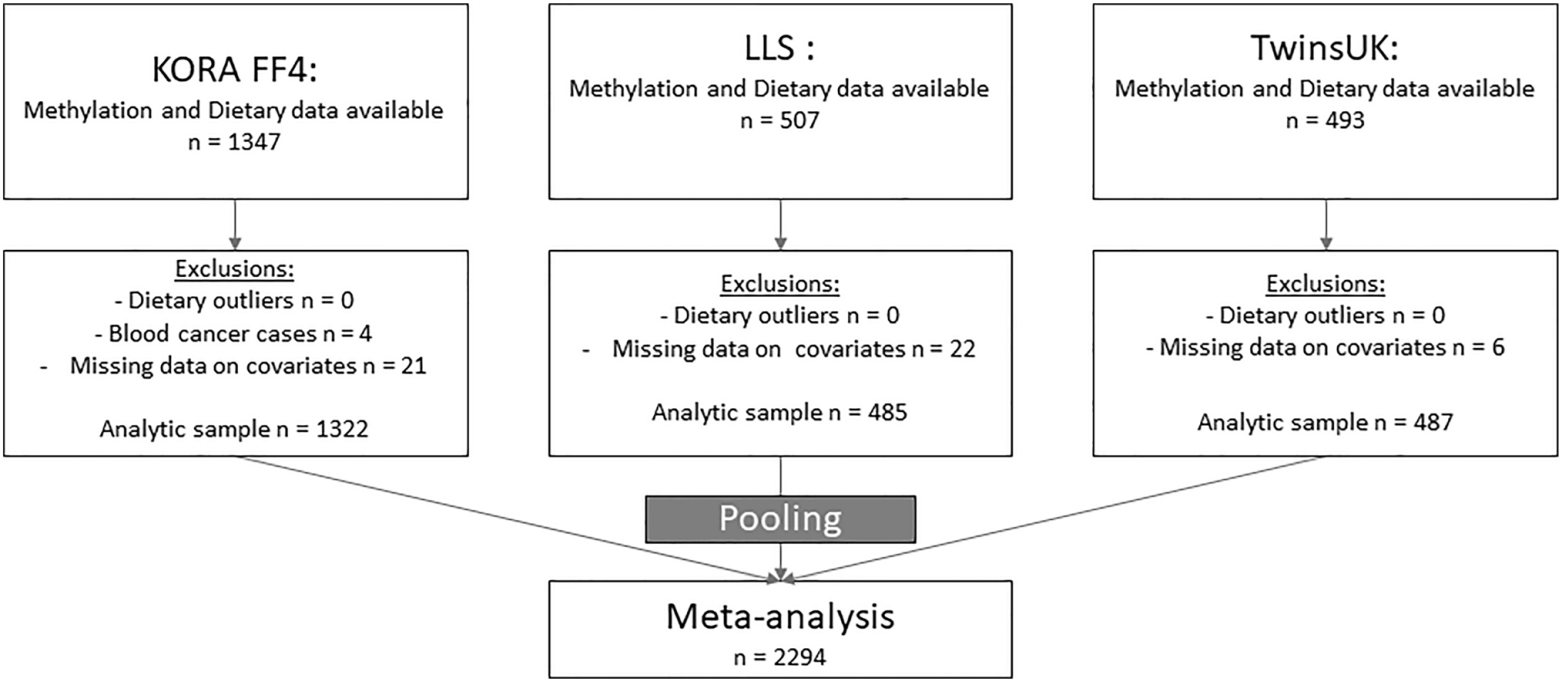
Flow chart of participant selection

The TwinsUK registry included over 14,000 research volunteer twin participants from the United Kingdom since 1992 [16]. Volunteers are monozygotic and dizygotic same-sex twins, predominately female (82%), middle-aged (mean age 59) and over 18 years-old. Volunteers were recruited without selecting for any particular disease or trait and are mostly of European descent. Data on volunteers were collected through longitudinal questionnaires and clinical visits. The registry collected biological samples and further data through analysis of biological samples. Dietary data was collected for > 3000 female twins, and blood DNA methylation data obtained within two years of food frequency questionnaires was available for 493 of the female twins. The caloric intake of all twins included in this study was within the 500–5000 kcal/day range. A total of 487 female twins had information on all covariates and were included in the food group EWASs. A flowchart for the study samples and final analysis sample is given in Fig. 1.

### Dietary intake

In the KORA FF4 study, dietary data was collected via repeated 24 h food lists, comprising 246 items and a food frequency questionnaire (FFQ), including 148 items. The 24 h food list was derived from the NAKO Health study [17] and subjects were asked to report the type of food they consumed. The FFQ was adapted from the German version of the multilingual European Food Propensity Questionnaire [18]. Usual dietary intake was modeled with the amount consumed (if consumed at all) based on portion sizes from the Bavarian consumption study II [19], multiplied by the probability of consumption for an individual subject from at least two non-consecutive 24 h food lists. This was done to reduce measurement error, which is prominent in surveyed dietary data. Further information regarding assessment of dietary intake data and estimation of usual dietary intake is provided elsewhere [20]. The dietary data is classified in 17 main food groups and 71 food subgroups according to the EPIC SOFT classification [21]. Nutrient intake data was calculated based on the German food composition database, Bundeslebensmittelschlüssel, version 3.01 [22].

As part of the LLS study, participants were sent a 218-item FFQ constructed from the 104-item VetExpress FFQ, combined with the Dutch National Food Survey [23]. Food items were categorized into 17 main food groups and 67 subgroups, with combination formulae used to split intake where appropriate.

Dietary data in TwinsUK was collected through a 131-item FFQ comprising the food and drink items originally included in the EPIC Norfolk study [24]. The processing of this data was first described elsewhere [25]. Here, the daily intake of each item was calculated in g/day using the FETA software [26], and the default nutritional database used was McCance and Widdowson’s The Composition of Foods (5th edition) [27]. Food items were then allocated to food groups following the EPIC-Soft classification, matching items successfully to 32 of 33 food groups.

After regressing food group intake against energy intake, the predicted food group intake was added for the mean energy intake of the study population to the residuals in all three cohorts to improve interpretability. Furthermore, two dietary patterns were calculated in each study: the Alternate Healthy Eating Index 2010 (AHEI 2010) [28] and the Mediterranean Diet Score (MDS) [29]. The AHEI scoring system assesses foods and nutrients predictive of chronic disease risk (e.g. vegetables, nuts, alcohol). A lower score is associated with higher risk of chronic diseases of major importance for public health. Due to a lack of data, trans fats had to be excluded in the calculation of AHEI, resulting in a maximum of 100 points instead of 110. Usual dietary intake was transformed to servings per day with references reported in [28]. A high MDS reflects high adherence to a dietary pattern followed by people living in Mediterranean countries, relative to the sex-specific population median, except for alcohol, where a moderate amount of consumption is ranked highest. The MDS emphasizes the consumption of fish, legumes, fruits and nuts, cereals, and a high ratio of unsaturated to saturated lipids. The modification of the MDS is depicted in the fat ratio as a sum of monounsaturated and polyunsaturated fatty acids divided by saturated fatty acids. The MDS is a population-based dietary score. The definition of food groups was harmonized based on the EPIC-Soft classification that was used to classify each food in all three cohorts, ensuring that individual food items were attributed to the same food (sub-) group. Harmonization was not entirely possible for mushrooms, milk, yogurt, eggs and plant oils, because at least one study did not capture these items.

### DNA methylation data

KORA FF4: Using the EZ-96 DNA Methylation Kit (Zymo Research, Orange, CA, USA) in two separate batches (*N* = 488, *N* = 1440), genomic DNA from white blood cells (750 ng) from 1928 participants of the KORA FF4 study was bisulfite-converted. According to standard protocols provided by Illumina, subsequent methylation analysis was performed on an Illumina (San Diego, CA, USA) iScan platform using the Infinium MethylationEPIC BeadChip. For initial quality control and to generate methylation data export files, GenomeStudio software version 2011.1 with Methylation Module version 1.9.0 was used.

Further preprocessing and quality control of the data were performed in R v3.5.1 [30] with the package minfi v1.28.3 [31] and following primarily the CPACOR pipeline [32]. Raw intensities were read into R (command read.metharray) and background corrected (bgcorrect.illumina). Hereafter probes with detection p values > 0.01 were set to missing.

We removed problematic samples and probes before normalization. Forty samples were removed: 33 had median intensity < 50% of the experiment-wide mean, or < 2000 arbitrary units, 9 (overlap of 4 with previous) had > 5% missing values on the autosomes and 2 showed a mismatch between reported sex and that predicted by minfi. A total of 59,631 probes were removed (some overlapping multiple categories): 5786 with > 5% missing values, cross-reactive probes as given in published lists (*N* = 44,493) [33, 34] and probes with SNPs with minor allele frequency < 5% at the CG position (*N* = 11,370) or the single base extension (*N* = 5597) as given by minfi. Finally, probes from the Y chromosome (*N* = 379) and the X chromosome (*N* = 17,743, following quality control) were excluded from the analysis. A total of 788,106 probes remained.

Quantile normalization was then performed separately on the signal intensities divided into the 6 probe types: type I green unmethylated, type I green methylated, type I red unmethylated, type I red methylated, type II red, type II green [32]. For the X and Y chromosomes, men and women were processed separately; for the autosomes, Quantile normalization was performed for all samples together. Methylation beta values, a measure from 0 to 1 indicating the percentage of cells methylated at a given locus, were generated out of the transformed intensities. The threshold for exclusion of beta-value outliers was set at ± 3* interquartile range.

The Infinium MethylationEPIC Manifest file (available at www.illumina.com via product files) was used to map probes to genes and chromosomes using genome build 37. The Manifest file uses the gene database of the University of California Santa Cruz (UCSC). Informed consent for genetic studies was obtained from all subjects. The protocol for each study was approved by the institutional review board of each cohort.

LLS: Venous blood samples were taken from 732 unrelated individuals aged between 40 and 75 for whole blood DNA methylation profiling. The Zymo EZ DNA methylation kit (Zymo Research, Irvine, CA, USA) was used to bisulfite-convert 500 ng of genomic DNA, and 4 μl of bisulfite-converted DNA was measured on the Illumina HumanMethylation450 array using the manufacturer’s protocol (Illumina, San Diego, CA, USA). Preprocessing and normalization of the data were done as described in the DNAmArray workflow (https://molepi.github.io/DNAmArray_workflow/).

In brief, IDAT files were read using the minfi, while sample-level quality control (QC) was performed using MethylAid. Filtering of individual measurements was based on detection *p* value (*p* < 0.01), number of beads available (≤ 2), or zero values for signal intensity. Normalization was done using functional normalization as implemented in minfi, using five principal components extracted using the control probes for normalization. All samples or probes with more than 5% of their values missing were removed.

TwinsUK: Whole-blood DNA methylation profiles in TwinsUK have previously been described [35]. Briefly, measurement of whole blood DNA methylation was performed using the Infinium HumanMethylation450 BeadChip (Illumina Inc, San Diego, CA) which profiles methylation levels at > 450,000 sites of the human genome. Processing of signals was performed using ENmix [36] for quality control, and minfi [31] to exclude samples with median methylated and unmethylated signals below 10.5. Both tools are available as Bioconductor software packages in R. During ENmix quality control checks, background and dye bias correction were performed as well as quantile normalization of signals. Bad probes and outlier samples were identified using standard parameter values, and signals with detP > 0.000001 and nbead < 3 were excluded. Beta-values were estimated after adjusting for differences in the distribution of type I and type II probe signals with the Regression on Correlated Probes (RCP) method. Beta-values out of the ± 3* interquartile distribution range were further excluded to match KORA FF4 exclusion criteria during association analyses. Maximum probe and sample missingness were set to 5%, and probes that mapped to multiple locations in the genome were removed. Overall, a total 430,768 autosomal probes and 487 individuals were included in our analysis.

Here we present the results of CpG sites that overlap between the Infinium MethylationEPIC and the Infinium HumanMethylation450 BeadChip, leaving a final number of at least 393,223 CpG sites per food group.

### Statistical analysis

The EWAS was carried out using linear regression analysis of the overlap of CpGs that were common in all three cohorts after quality control (*n* = 393,427). We performed a fixed-effect meta-analysis, because the estimated tau is considered imprecise with a small sample of studies [37]. In addition, we did a random-effects meta-analysis as a sensitivity analysis to follow-up on significant signals by evaluating the unadjusted p value. In context of the often high heterogeneity observed, we reported the *I*^2^ confidence interval, which is recommended in a small sample meta-analysis [38]. *N* = 1321 subjects from KORA FF4, *N* = 507 subjects from LLS and *N* = 487 subjects from TUK were included in the analysis, resulting in a sample size of *N* = 2315. The primary outcome of this study was methylation beta values. We tested 37 food groups, nutrients and diet quality scores: potatoes, total vegetables, leafy vegetables, fruit vegetables, root vegetables, cabbage vegetables, onions and garlic, legumes, total fruits, nuts and seeds, milk, yogurt, cheese, cream, grain products, whole grain products, total meat, fresh red meat, processed meat, total fish, eggs, plant oils, butter, margarine, total sweets, cakes, sugar-sweetened beverages, coffee, tea, wine, beer, spirits, AHEI, MDS and folic acid. The residual method was used in each cohort to get intake estimates independent of total energy intake [39]. The *p* values were false-discovery rate (FDR) corrected (*p* < 0.05) using the Benjamini and Hochberg procedure. Methylation as beta values were regarded as the dependent variable. Exposures were food groups (g/day), dietary pattern scores (integer) and additionally folic acid in µg/day. Selected covariates for the model were sex, age (continuous), age squared, BMI (continuous), BMI squared, total caloric intake (continuous), alcohol in g/day (continuous—not applied in the analysis of wine, beer, spirits, AHEI and MDS), measured or estimated cell counts (using the Houseman-method [40]), smoking behavior (regular, former, never) and methylation plate and/or plate position as a technical variable. These were selected based on the literature and our own assessment of confounding with the disjunctive cause criterion [41]. Neutrophile granulocytes were excluded as a covariate due to multicollinearity. Only complete cases for every covariate were included in the analysis. To account for heterogeneity, we inspected and reported the *p* value of the Q-statistic and *I*^2^ for all CpGs that reached statistical significance. All statistical analyses were carried out with R statistical software version 4.0.4 [30]. Meta-analysis was performed with the metagen function of the meta package version 4.17.0 [42]. Figures were created using the ggplot2 package [43]. To evaluate whether CpGs were occuring in differentially methylated regions, DMRfinder [44] was used to test for the occurrence of significant CpGs < 1 kb apart as implemented in DNAmArray.

## Results

**Fig. 2:**
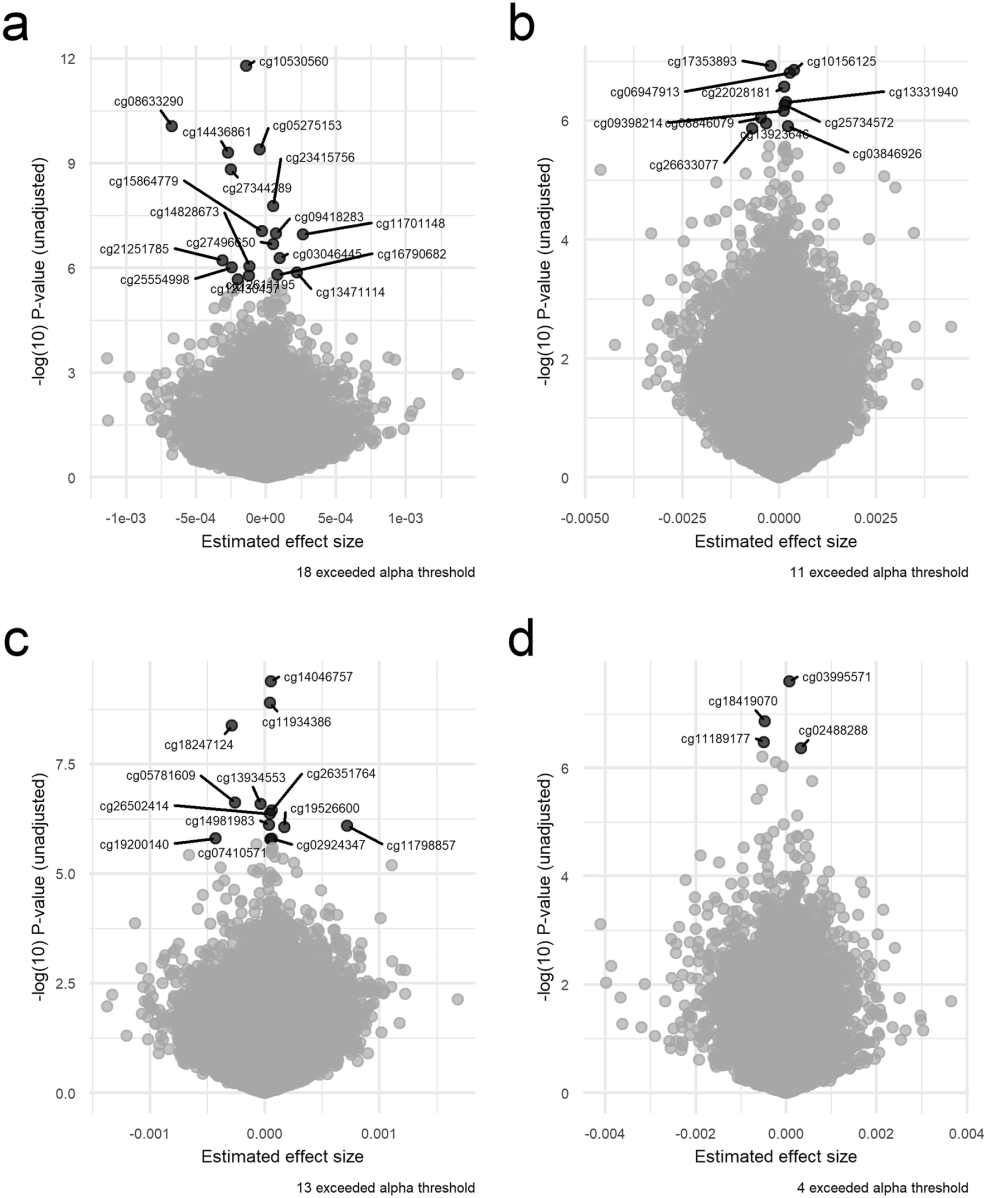
Volcano plots with the unadjusted *p* value on the y-axis. Every significant CpG after FDR adjustment is marked with its probeID. Effect size on the x-axis is %-methylation change per gram residual/day. **a** nuts and seeds, **b** cream, **c** butter, **d** plant oils in g/day residuals

**Fig. 3:**
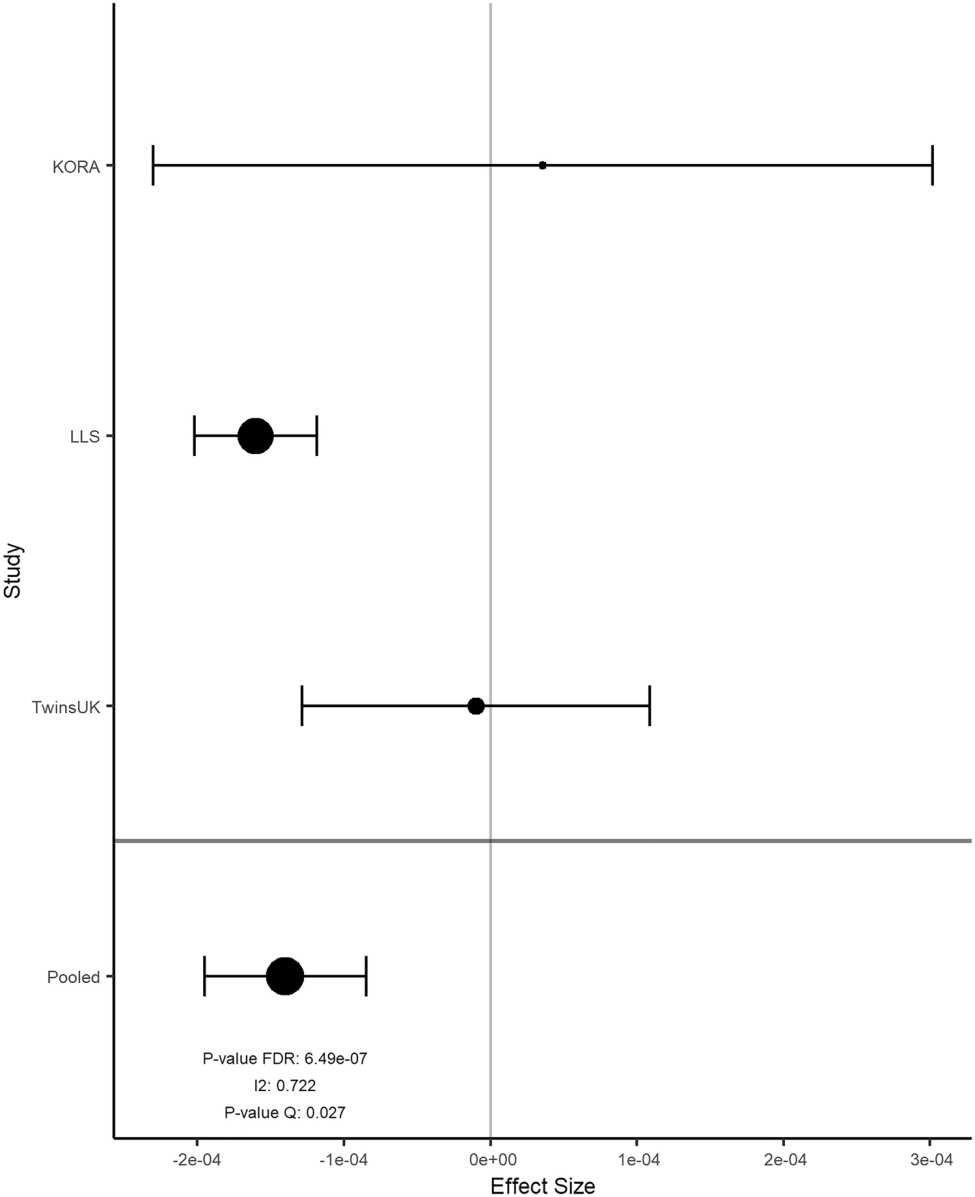
Forest plot for the association between cg10530560 methylation level and nuts and seeds consumption. Effect size on the x-axis is %-methylation change per gram residual/day with a 95% confidence interval

**Fig. 4:**
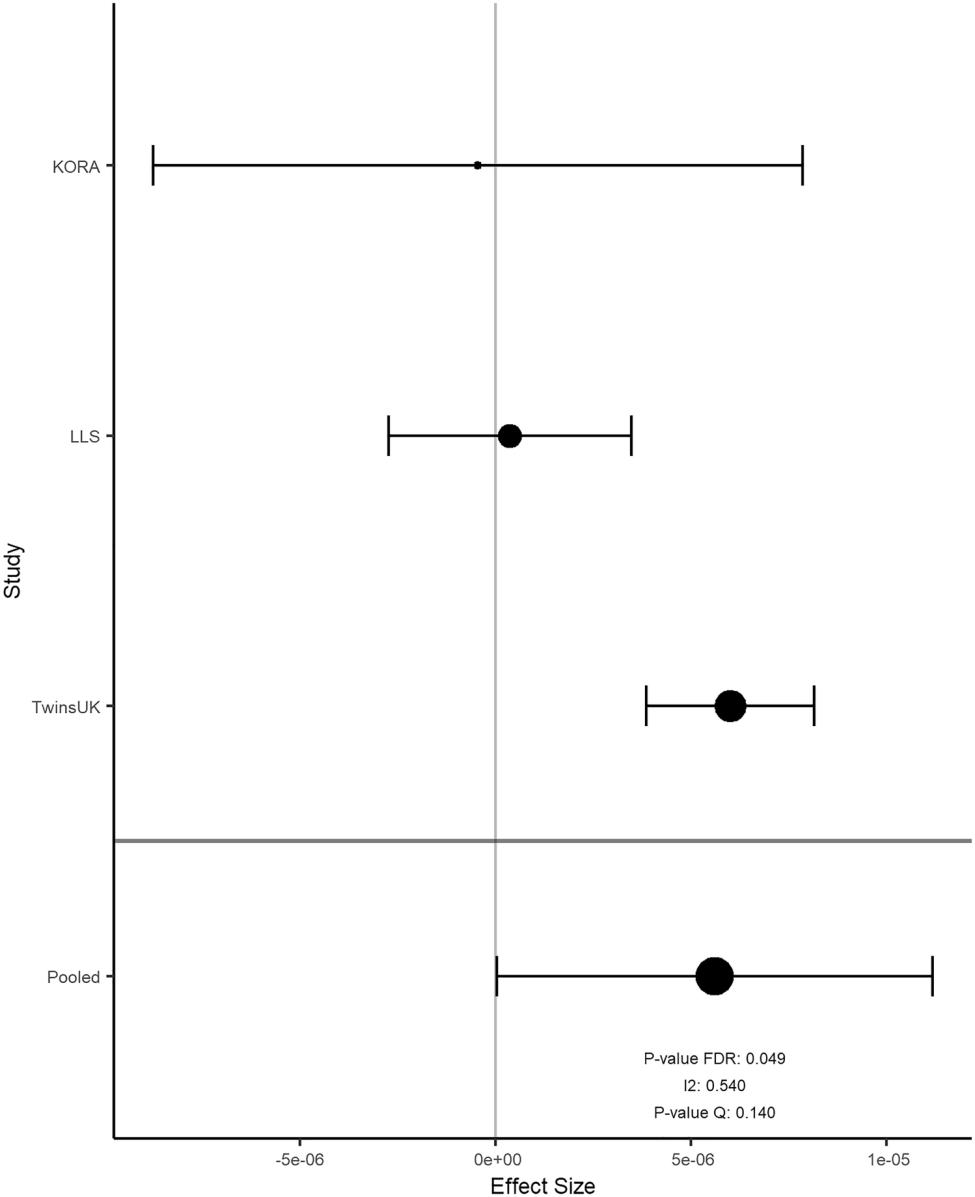
Forest plot for the association between cg14732699 methylation level and milk consumption. Effect size on the x-axis is %-methylation change per gram residual/day with a 95% confidence interval

**Fig. 5:**
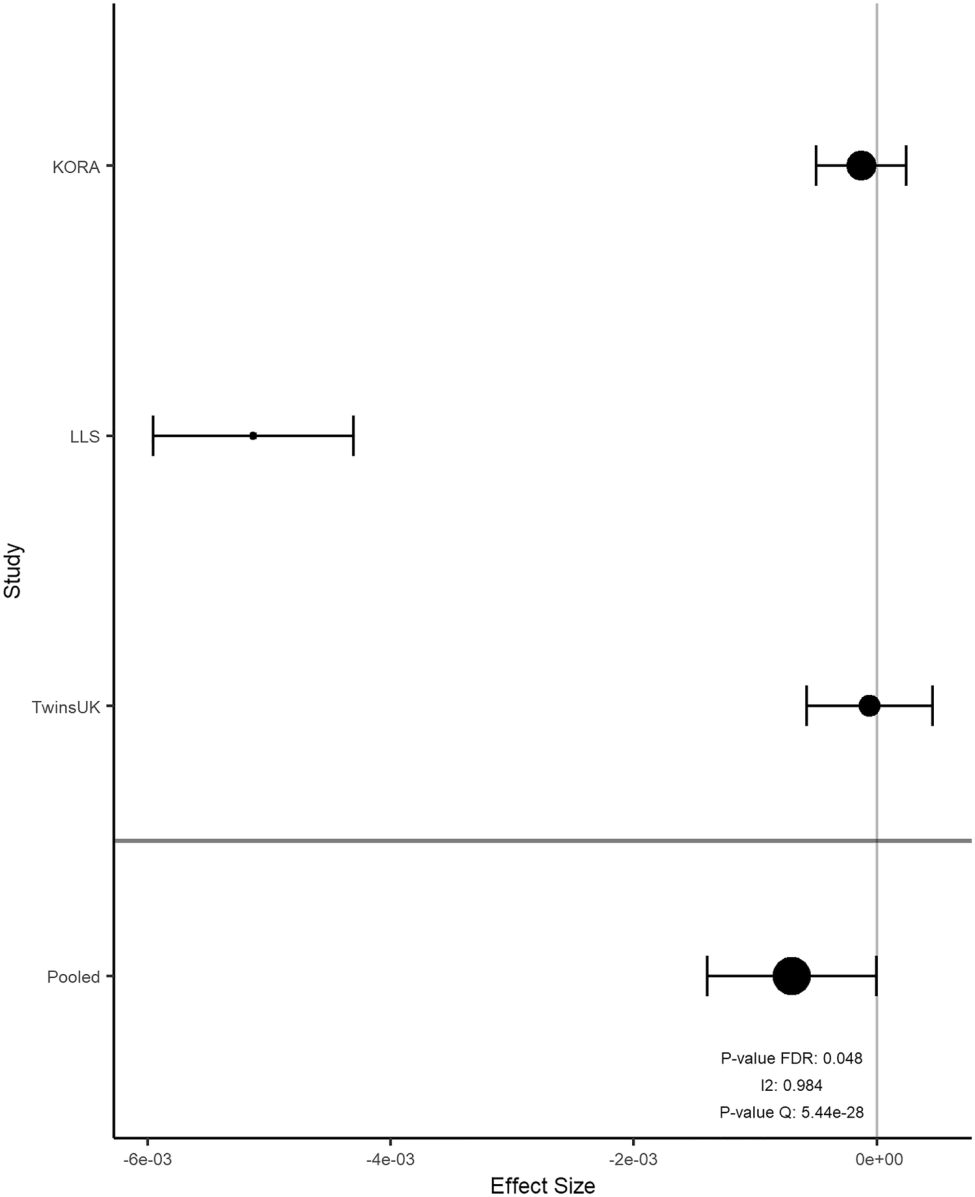
Forest plot for the association between cg26633077 methylation level and cream consumption. Effect size on the x-axis is %-methylation change per gram residual/day with a 95% confidence interval

**Fig. 6:**
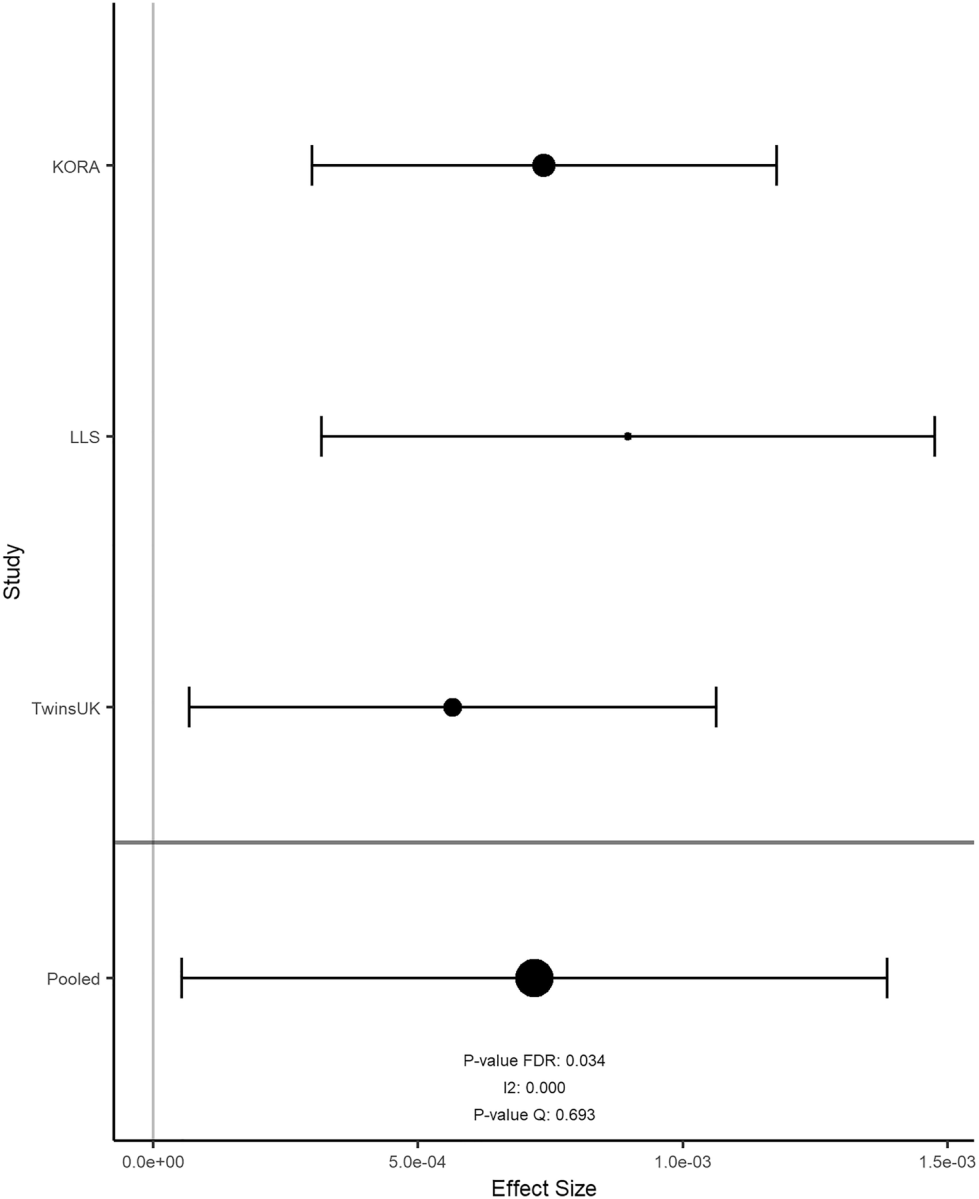
Forest plot for the association between cg11798857 methylation level and butter consumption. Effect size on the x-axis is %-methylation change per gram residual/day with a 95% confidence interval

**Fig. 7:**
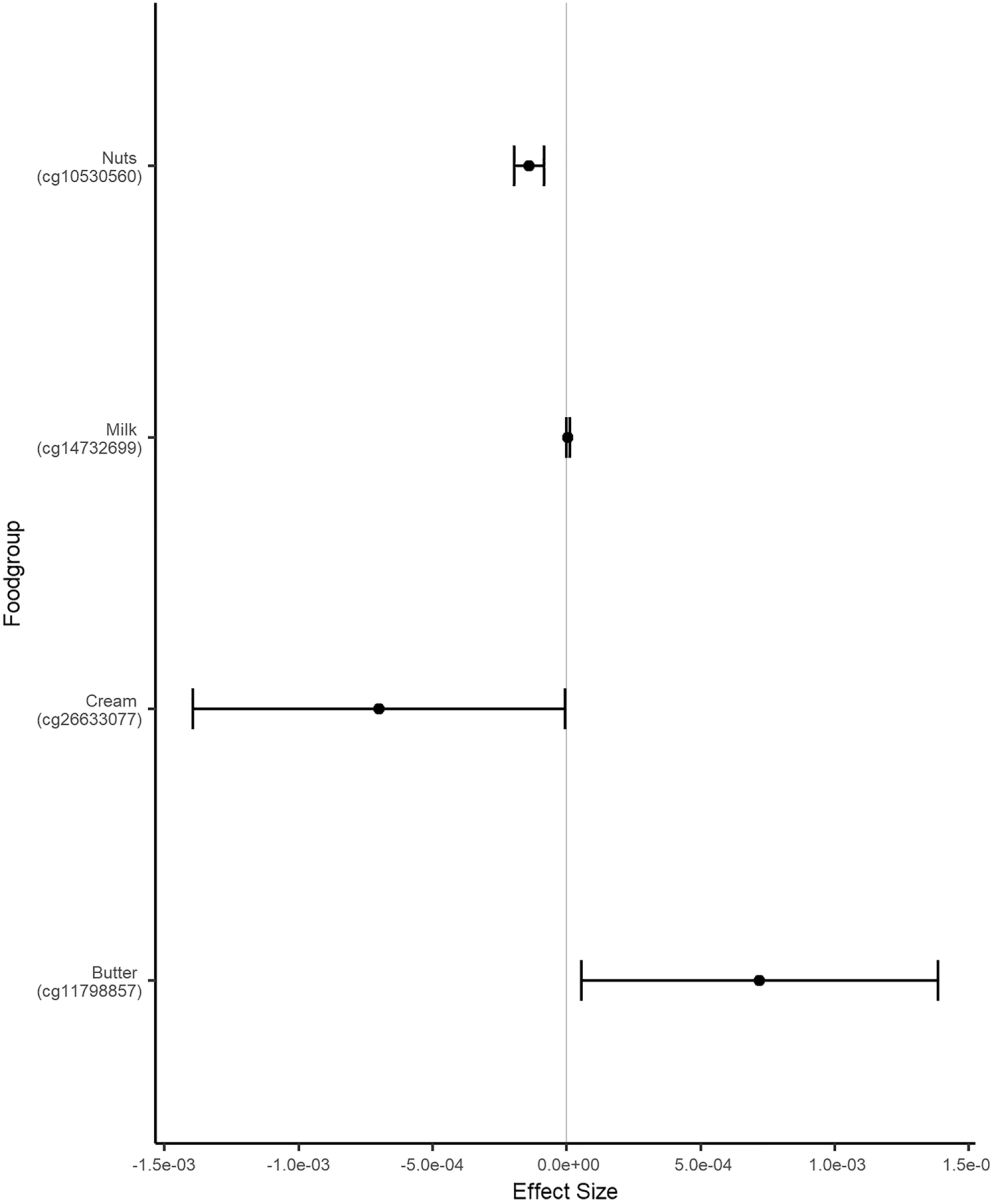
Combined forest plot of pooled estimators. One significant finding in different food groups is shown to get a perspective for the different effect sizes. Effect size on the x-axis is %-methylation change per gram residual/day with a 95% confidence interval

Overall, the results of 2316 participants were included in the meta-analysis. In KORA FF4, LLS and TUK, participants had a median age of 58, 59, and 60 years; a median BMI of 26.8, 25.1, and 25.6 kg/m^2^; and a median total energy intake of 1820, 1883, and 1808 kcal/day, respectively (Table 2). Intake of food groups for all cohorts can be found in Online Resource 1. Following a false-discovery rate adjustment with an alpha threshold at 0.05 (Table 3), we found 2 significant associations for onions and garlic consumption, 18 for nuts and seeds (Figs. 2a and 3), one for milk (Fig. 4), 11 for cream (Figs. 2b and 5), 13 for butter (Figs. 2c and 6), four for plant oils (Fig. 2d), five for wine, 16 for beer and six for spirits (for alcoholic beverages results, see Online Resource 2). We obtained no statistically significant signals for other food groups or dietary patterns. All significant CpGs were located in distinct regions (inter-CpG-distance > 1 kb). Some interesting annotated genes that are linked to metabolism include: *GLI1* (Fig. 3), *ATP5H*, *MYC*, *RPTOR*, *ASAM*, *FOXA2*, and *DIO1.* Cg26633077 lies within the gene body of RPTOR, which could lead to suppressed gene expression with more cream consumption, as indicated by the negative effect size. This gene is involved in a signaling pathway that regulates cell growth in response to nutrient levels. Cg11798857 is positioned at the promoter of the FOXA2 gene. Combined with a positive effect size, this would indicate gene suppression as well. FOXA2 is a transcriptional activator for liver-specific genes. Figure 5 shows the forest plot of the CpG associated with MYC, which is a pro-fibrotic regulator. See Table 3 for information on all annotated genes and locations of the CpGs. Figure 7 displays examples of effect size estimates for the association of different food groups with DNA methylation. Two of the identified CpGs were detected in two distinct food groups, namely wine and beer. The first locus was annotated to the *PHGDH* gene, which is involved in the early steps of L-serine synthesis (cg14476101) and the second to *TRA2B*, which plays a role in mRNA processing (cg12825509).

**Table 2:** Population characteristics stratified by sex and cohort

	KORA			LLS			TUK	
	Male	Female	Overall	Male	Female	Overall	Male	Female
*n*	620	702	1322	240	267	507	NA	487
Age in years (median [IQR])	59.0 [49.0, 67.0]	58.0 [48.0, 65.0]	58.0 [49.0, 66.0]	60.7 [55.9, 64.9]	57.5 [53.0, 61.8]	58.9 [54.5, 63.5]	NA	59.5 [52.2, 65.5]
BMI in kg/m^2^ (median [IQR])	27.4 [25.2, 30.5]	26.1 [23.2, 29.8]	26.8 [24.1, 30.2]	25.3 [23.6, 27.2]	24.6 [22.4, 26.9]	25.1 [23.0, 27.1]	NA	25.6 [23.1, 29.3]
Total energy intake (median [IQR])	2093.8 [1885.9, 2332.7]	1578.9 [1427.9, 1791.8]	1819.8 [1550.8, 2114.8]	2215.3 [1771.9, 2576.9]	1730.5 [1465.0, 2008.3]	1882.5 [1573.9, 2341.5]	NA	1808.1 [1473.1, 2199.3]
Alcohol in g/day (median [IQR])	13.2 [5.1, 24.6]	2.7 [1.7, 5.3]	5.0 [2.4, 13.9]	16.1 [8.4, 28.0]	9.0 [2.9, 19.1]	12.5 [4.5, 89.6]	NA	5.4 [0.9, 12.6]
Smoking behavior (%)								
Regular smoker	96 (15.5)	97 (13.8)	193 (14.6)	41 (11.5)	44 (11.3)	85 (11.6)	NA	60 (12.3)
Former smoker	283 (45.6)	222 (31.6)	505 (38.2)	196 (54.9)	162 (41.8)	358 (48.9)	NA	162 (33.2)
Never smoker	241 (38.9)	383 (54.6)	624 (47.2)	67 (18.8)	132 (34.0)	199 (27.2)	NA	265 (54.4)
Physical activity: active (%)	361 (58.2)	452 (64.4)	813 (61.5)	240 (100)	267 (100)	507 (100)	NA	NA

Values are presented as median [Interquartilrange]

**Table 3:** Significant results of the meta-analyzed EWAS of KORA FF4, TwinsUK and Leiden Longevity Study

ProbeID	Studies*	Effect- size**	*p* value FDR	*p* value Q-statistic	*I*^2^***	Foodgroup	Chr	RefGene name	RefGene group	Relation to CpG Island
cg06618277	K–L–T	− 1.39e-04	0.050	0.215	0.349 [0.000;0.789]	Onions-garlic	13	N/A	N/A	N/A
cg13970894	K–L–T	− 4.16e-04	0.050	0.706	0.000 [0.000;0.702]	Onions-garlic	10	N/A	N/A	N_Shore
cg03046445	K–L–T	9.94e-05	0.019	0.012	0.774 [0.266;0.930]	Nuts-seeds	12	BHLHE41*	1stExon*	N_Shore
cg05275153	K–L–T	− 4.39e-05	4.96e-05	3.14e-06	0.921 [0.801;0.969]	Nuts-seeds	4	RGS12*	Body*	N/A
cg08633290	K–L–T	− 6.72e-04	1.70e-05	2.79e-05	0.905 [0.748;0.964]	Nuts-seeds	19	N/A	N/A	N_Shore
cg09418283	K–L–T	7.31e-05	0.005	0.013	0.770 [0.252;0.929]	Nuts-seeds	12	PAWR*	1stExon*	Island
cg10530560	K–L–T	− 1.40e-04	6.49e-07	0.027	0.722 [0.060;0.918]	Nuts-seeds	12	GLI1*	5'UTR*	S_Shelf
cg11701148	K–L–T	2.64e-04	0.005	0.134	0.503 [0.000;0.856]	Nuts-seeds	8	MYOM2	TSS200	N/A
cg12430457	K–L–T	− 2.00e-04	0.047	4.47e-04	0.870 [0.630;0.955]	Nuts-seeds	12	SYT1*	5'UTR*	N/A
cg12611195	K–L–T	− 1.22e-04	0.038	9.64e-11	0.957 [0.905;0.980]	Nuts-seeds	6	PPP1R14C	Body	N/A
cg13471114	K–L–T	2.21e-04	0.036	0.190	0.397 [0.000;0.814]	Nuts-seeds	2	OTX1	TSS1500	Island
cg14436861	K–L–T	− 2.70e-04	4.96e-05	2.89e-07	0.934 [0.840;0.972]	Nuts-seeds	11	WEE1*	3'UTR*	N/A
cg14828673	K–L–T	− 1.17e-04	0.027	0.022	0.739 [0.126;0.922]	Nuts-seeds	8	TOP1MT	Body	N/A
cg15864779	K–L–T	− 2.77e-05	0.005	0.995	0.000 [0.000;0.000]	Nuts-seeds	17	ATP5H*	TSS200*	Island
cg16790682	K–L–T	8.13e-05	0.038	0.007	0.798 [0.360;0.936]	Nuts-seeds	12	PIP4K2C*	TSS200*	Island
cg21251785	K–L–T	− 3.12e-04	0.020	2.08e-10	0.955 [0.901;0.980]	Nuts-seeds	9	TRPM3*	TSS1500*	N/A
cg23415756	K–L–T	5.25e-05	0.001	0.273	0.229 [0.000;0.920]	Nuts-seeds	17	NTN1	Body	Island
cg25554998	K–L–T	− 2.43e-04	0.027	0.005	0.811 [0.411;0.940]	Nuts-seeds	11	N/A	N/A	N_Shore
cg27344289	K–L–T	− 2.49e-04	1.19e-04	0.002	0.834 [0.497;0.945]	Nuts-seeds	5	FLJ41603	Body	N/A
cg27496650	K–L–T	5.30e-05	0.008	0.363	0.012 [0.000;0.897]	Nuts-seeds	8	TOX	TSS1500	Island
cg14732699	K–T	5.60e-06	0.049	0.140	0.540 [0.000;0.887]	Milk	8	MYC	Body	Island
cg03846926	K–L–T	2.26e-04	0.048	0.007	0.799 [0.363;0.936]	Cream	10	C10orf140	5'UTR	S_Shore
cg06947913	K–L–T	2.82e-04	0.020	3.95e-14	0.968 [0.934;0.984]	Cream	12	FAIM2	TSS200	Island
cg08846079	K–L–T	− 4.73e-04	0.044	0.518	0.000 [0.000;0.842]	Cream	1	N/A	N/A	N/A
cg09398214	K–L–T	1.21e-04	0.038	5.70e-08	0.940 [0.859;0.975]	Cream	17	MARCH10*	Body*	Island
cg10156125	K–L–T	3.86e-04	0.020	5.53e-11	0.958 [0.908;0.981]	Cream	4	UGT8*	5'UTR*	Island
cg13331940	K–L–T	1.82e-04	0.036	7.79e-16	0.971 [0.943;0.986]	Cream	15	MYO1E	Body	Island
cg13923646	K–L–T	− 3.30e-04	0.048	0.234	0.311 [0.000;0.928]	Cream	1	N/A	N/A	N/A
cg17353893	K–L–T	− 2.15e-04	0.020	2.07e-31	0.986 [0.975;0.992]	Cream	7	CLIP2*	Body*	Island
cg22028181	K–L–T	1.31e-04	0.026	7.67e-21	0.978 [0.959;0.989]	Cream	15	CYFIP1	TSS200	Island
cg25734572	K–L–T	1.46e-04	0.036	9.21e-05	0.892 [0.707;0.960]	Cream	19	N/A	N/A	Island
cg26633077	K–L–T	− 6.99e-04	0.048	5.44e-28	0.984 [0.972;0.991]	Cream	17	RPTOR*	Body*	S_Shelf
cg02488288	K–L	3.29e-04	0.044	0.006	0.868 [0.481;0.967]	Plant-oils	5	SPRY4*	TSS1500*	N_Shore
cg03995571	K–L	7.05e-05	0.010	0.015	0.833 [0.303;0.960]	Plant-oils	8	FAM49B	TSS1500	Island
cg11189177	K–L	− 4.92e-04	0.044	3.95e-04	0.920 [0.726;0.977]	Plant-oils	17	ABR*	Body*	S_Shore
cg18419070	K–L	− 4.78e-04	0.028	0.018	0.820 [0.239;0.957]	Plant-oils	3	SEMA5B	Body	N/A
cg02924347	K–L–T	6.31e-05	0.049	0.875	0.000 [0.000;0.220]	Butter	16	ABCA17P*	TSS1500*	Island
cg05781609	K–L–T	− 2.57e-04	0.020	2.48e-14	0.968 [0.935;0.984]	Butter	1	COL24A1	Body	N/A
cg07410571	K–L–T	5.11e-05	0.049	0.718	0.000 [0.000;0.685]	Butter	20	DDRGK1	TSS200	Island
cg11798857	K-L–T	7.20e-04	0.034	0.693	0.000 [0.000;0.716]	Butter	20	FOXA2*	TSS1500*	N_Shore
cg11934386	K–L–T	4.75e-05	2.49e-04	5.55e-10	0.953 [0.896;0.979]	Butter	7	C7orf41*	1stExon*	Island
cg13934553	K–L–T	− 3.64e-05	0.020	5.21e-04	0.868 [0.621;0.954]	Butter	1	PLEKHG5*	Body*	Island
cg14046757	K–L–T	5.29e-05	1.63e-04	1.65e-20	0.978 [0.959;0.988]	Butter	14	ZC3H14*	TSS200*	Island
cg14981983	K–L–T	3.95e-05	0.034	1.03e-12	0.964 [0.924;0.983]	Butter	19	ZNF527	TSS200	Island
cg18247124	K–L–T	− 2.85e-04	5.45e-04	5.87e-12	0.961 [0.918;0.982]	Butter	11	ASAM	3'UTR	N/A
cg19200140	K–L–T	− 4.27e-04	0.049	0.188	0.402 [0.000;0.816]	Butter	20	N/A	N/A	N_Shelf
cg19526600	K–L–T	1.70e-04	0.034	0.825	0.000 [0.000;0.461]	Butter	1	DIO1*	1stExon*	N/A
cg26351764	K–L–T	6.36e-05	0.024	1.03e-12	0.964 [0.924;0.983]	Butter	20	FAM83D	TSS200	Island
cg26502414	K–L–T	4.70e-05	0.025	0.798	0.000 [0.000;0.538]	Butter	17	METT10D	5'UTR	N_Shore

*UCSC RefGene Name* Target gene names from the UCSC database, *UCSC RefGene Group* Describing CpG position. TSS1500 = 200–1500 bases upstream of the Transcription start site (TSS), *5-UTR* Within the 5' untranslated region, between the TSS and the ATG start site; Body = Between the ATG and stop codon, irrespective of the presence of introns, exons, TSS or promoters, *3'UTR* Between the stop codon and the poly A signal, *Relation to UCSC CpG Island* The location of the CpG relative to the CpG Island. Shore = 0–2 kb from Island; Shelf = 2–4 kb from Island, *N *Upstream (5') of CpG Island, *S* Downstream (3') of CpG Island [66]

*K is short for KORA FF4 inclusion; L is short for Leiden longevity study inclusion; T is short for TwinsUK inclusion

**Effect sizes are %-methylation change per g/day residual intake

***I^2^ is reported with 95%-confidence interval in brackets as calculated by the metagen function of the meta package

*After a gene name indicates available splice variants

Many of the food groups for which we observed significant associations are high in fat content. However, in contrast to this statement, we found no significant signals in case of cheese, eggs or margarine consumption. We explored whether significant CpGs identified in one food group may also be associated with another (high-fat) food group. We chose the example of the findings for nuts and seeds, and Table 4 displays the results. In total for all explored food groups, 10 signals from the food group nuts and seeds showed an unadjusted *p* value < 0.05 in other high-fat food groups, and only two of them had the same direction of effect [cg09418283, cg10530560]. We did not observe any significant association for the consumption of food groups that are well known for their specific phytochemical content, such as leafy vegetables, cabbage vegetables and fruits, or coffee and tea. We also did not observe any DNA methylation association with AHEI or MDS.

**Table 4:** *p* values for high-fat food groups for loci with significant associations with the food group nuts and seeds

CpG	Nuts-seeds	Butter	Cheese	Cream	Eggs	Margarine	Plant-oils	Processed-meat
cg03046445	5.27e–07*	0.217	0.413	0.328	0.549	0.102	0.901	0.387
cg05275153	4.10e–10*	0.725	0.760	0.189	0.766	0.377	0.170	0.600
cg08633290	8.66e–11*	0.118	0.118	0.853	0.489	0.170	0.712	0.915
cg09418283	1.04e–07*	0.427	0.942	0.185	0.021*	0.758	0.452	0.082
cg10530560	1.65e–12*	0.821	0.046*	0.929	0.043*	0.720	0.171	0.088
cg11701148	1.11e–07*	0.251	0.230	0.574	0.672	0.785	0.299	0.764
cg12430457	2.15e–06*	0.636	0.986	0.343	0.790	0.311	0.304	0.423
cg12611195	1.66e–06*	0.223	0.033*	0.748	0.479	0.013*	0.443	0.051
cg13471114	1.37e–06*	0.870	0.979	0.608	0.240	0.372	0.452	0.085
cg14436861	5.04e–10*	0.865	0.598	0.246	0.968	0.013*	0.843	0.053
cg14828673	8.97e–07*	0.610	0.751	0.942	0.627	0.993	0.946	0.589
cg15864779	8.95e–08*	0.232	0.773	0.210	0.810	0.819	0.898	0.082
cg16790682	1.57e–06*	0.526	0.469	0.929	0.744	0.021*	0.472	0.169
cg21251785	6.06e–07*	0.518	0.547	0.321	0.427	0.665	0.254	0.890
cg23415756	1.68e–08*	0.045*	0.999	0.745	0.812	0.956	0.606	0.317
cg25554998	9.77e–07*	0.088	0.876	0.409	0.551	0.401	0.688	0.502
cg27344289	1.51e–09*	0.155	0.470	0.921	0.396	0.730	0.921	0.148
cg27496650	2.11e–07*	0.812	0.394	0.080	0.002*	0.461	0.173	0.284

Underlining indicates same direction of effect

*Indicates *p* value < 0.05

In many cases, heterogeneity between studies was high, with *I*^2^ > 0.8 (Table 3). Reasons could be differences in dietary assessment methods across studies or differences between populations. To explore this further, we also performed a random-effects meta-analysis, which reproduced 2 of 2 signals in onions and garlic [cg06618277; cg13970894], 7 out of 18 in nuts and seeds [cg03046445; cg11701148; cg13471114; cg15864779; cg23415756; cg27344289; cg27496650], 0 of 1 in milk, 3 of 11 in cream [cg03846926; cg08846079; cg13923646], 6 of 13 in butter [cg02924347; cg07410571; cg11798857; cg19200140; cg19526600; cg26502414], 2 of 4 in plant oils [cg02488288; cg18419070], 5 of 5 in wine [cg06690548; cg07856667; cg08033640; cg12825509; cg14476101], 10 of 16 in beer [cg01794805; cg03044533; cg03725309; cg06469895; cg07714319; cg08984272; cg10797552; cg11100157; cg11376147; cg15821562], and 1 of 6 in spirits [cg09307985]. Detailed results are listed in Online Resource 3. For further information regarding heterogeneity and effect size distribution, see Online Resource 4, where the p value distribution, *I*^2^ distribution and estimated tau distribution for every analyzed food group with significant signals are displayed. Online Resource 5 presents volcano plots for every analyzed food group.

## Discussion

This work explored many food groups that have not been studied in context of human DNA methylation, e.g., nuts and seeds, or added fats and oils. Our main finding is that the majority of analyzed food groups did not show significant associations with blood DNA methylation, and that significant associations with methylation levels were observed primarily for food groups high in fat content.

We evaluated whether the CpGs we found to be associated with food groups in this analysis had been previously identified in EWAS for other traits using the EWAS catalog [45]. Many significant associations (cg12825509, cg14476101, cg06690548, cg11376147, cg14476101, cg06469895, cg12825509, cg18120259, cg03725309, cg07714319, cg16246545, cg15821562, cg03044533, cg26282731, cg11100157, cg01794805) observed in our analysis on alcoholic beverages could be attributed to their ethanol content, and are already reported in the EWAS catalog for their association with alcohol consumption. Loci cg12430457 (nuts and seeds), cg06947913 (cream) and cg14046757, cg13934553, cg26502414, cg07410571 (butter) were all reported to be associated with rheumatoid arthritis [45]. One signal in nuts and seeds, cg14828673, was previously reported to be associated with waist-to-hip-ratio [45]. Surprisingly, cg13331940, which was significantly associated with cream, was previously reported to be associated with alcohol consumption per day [45]. None of our remaining significant signals were associated with metabolic traits, metabolic diseases or dietary exposures in past EWAS.

We found several interesting signals in the food group nuts and seeds for which there is a reported connection in the literature. Cg10530560 maps to the gene *GLI1* and showed a significant association with the food group nuts and seeds. *GLI1* is a transcription factor which gets activated by and is a marker of the sonic hedgehog pathway [46]. A negative effect size and the location in the gene body could be interpreted as a downregulation in gene expression, which would fit the downregulation of genes in the hedgehog pathway triggered by a diet high in either saturated or unsaturated fatty acids as reported by Mehmood et al. [46]. Deactivation of the hedgehog pathway is suggested to be associated with fat accumulation [47]. Another significant signal (cg15864779, located within the ATP5H gene) could possibly be explained by the high-methionine content in nuts. A high-methionine diet alters the ATP5H expression dependent on the paraoxonase genotype. Paraoxonase-positive mice have downregulated ATP5H, whereas paraoxonase-negative mice had upregulated ATP5H. This interaction is tightly linked to energy generation in the hyperhomocysteinemic liver [48].

The one CpG linked to milk consumption, cg14732699, is associated with *MYC,* a pro-fibrotic regulator. Butyric acid as a component in bovine milk triglycerides [49] could have affected the methylation of this *MYC* CpG site. One study identified butyrate as a protective agent for diet-induced non-alcoholic hepatic steatosis and liver fibrosis by downregulating, among other, *MYC* [50]. Another study observed an association between oleic acid, the main monounsaturated fatty acid in bovine milk, and the gene *MYC.* It showed that oleic acid promotes colorectal cancer development by upregulation of *MYC*, among others [51].

We also observed significant associations with cream consumption, another high-fat food group. *CLIP2* associated with cg17353893 is reported to be downregulated under a high-fat diet regimen [52]. This downregulation also fits our results, where cg17353893 has a negative effect size and is located within the gene body [53]. The *CYFIP1* (cg22028181) gene is a homolog of *CYFIP2,* which was described as a genetic factor underlying compulsive-like binge eating in mice [54]. *CYFIP1* haploinsufficiency shows similar properties by increasing compulsive-like behavior and modulation of palatable food intake in mice [55]. Cream is a food with very high energy density; thus, dependent on the direction of the relationship, gene methylation could be either the cause or effect of cream consumption. Calorie intake impacts the gene associated with cg26633077, *RPTOR*, as shown in the stabilization of the MTOR-RPTOR association by nutrient deprivation, leading to inhibition of MTOR activity [56]. Despite the inhibition of the anabolic regulator MTOR, one study found that *RPTOR* null mice gained less weight, most likely due to reduced food intake in a high-fat diet, when compared to wild type mice [57]. It is worth noting that there was very high heterogeneity observed for cg26633077.

More insight into the association between CpG methylation and adiposity can be given by significant associations with butter intake. Cg18247124 is located in adipocyte adhesion molecule (*ASAM)*, which was found to be correlated with BMI in human subcutaneous adipose tissue, and ASAM mRNA is increased during adipocyte differentiation in mice and humans [58]. Also, cg11798857 in the transcription start site of *FOXA2* was a significant finding in our analysis. *FOXA2* mRNA, related to fatty acid oxidation in the liver, was increased in mice fed with pre- and probiotics, along with improved insulin sensitivity and reduced adipocyte size [59]. *DIO1* (cg19526600) encodes for type I iodothyronine deiodinase and can affect lipid metabolism through its effects on thyroid hormones. Xia et al. [60] reported that mice with an obese phenotype experienced ameliorated hepatic steatosis if the intervention was exercise, low-fat, quercetin or calorie restriction, possibly by affecting miRNAs, e.g. miR-383 and miR-146b to elevate *DIO1* expression.

Comparing all of our results to previous EWAS is quite difficult because of the lack of EWAS analyzing food groups. Karabegovic et al. performed an EWAS in four European cohorts analyzing tea and coffee consumption. We tried to replicate the findings of Karabegovicet al. [61] for coffee with a Bonferroni adjusted alpha (0.05) solely in the KORA FF4 study, but failed, except for cg25648203, for which we could confirm the direction of effect. We did not observe significant signals in our meta-analysis of coffee and DNA methylation. There are obvious differences that could explain the failed replication. The study from Karabegovic et al. has ten times the sample size that our study has, which greatly increases the power to detect such signals. Also, while Karabegovic et al. used their coffee intake in cups per day, ours is measured as usual dietary intake in g/day and used as residuals in the linear regression.

Several pathways could assist in explaining the associations between food groups and methylation changes. One of our hypotheses was that the link between diet and inflammation could influence DNA methylation levels. Nuts are known for their high unsaturated and low saturated fatty acid content, which can affect homeostasis of inflammation and therefore impact DNA methylation patterns [3]. However, this argument has to be evaluated for every food group separately. Nuts, butter, plant oils and cream have a high-fat content in common, which could also either trigger or reduce inflammation in mice [62], but not in obese humans without metabolic disturbances [63]. Other food groups like red meat or cabbage that were associated with inflammatory processes in the past have not yielded any signals. Further studies are needed to confirm our results that the association of, for example, red meat and cabbage with inflammation are independent of DNA methylation.

Although our results hint at a pattern suggesting that the high-fat content of the food groups seems to be a major determinant in the modification of methylation patterns, the results as described in Table 4 do not confirm this regarding the significant signals found for the food group nuts and seeds. Additionally, we observed only a few or no significant signals in other high-fat content food groups like fish, processed meat and cheese.

Despite the focus on food groups, we also analyzed folic acid intake in this meta-analysis. We found no significant association here, which supports the theory that nutrients involved in the pathway that leads to the main methyl donor S-adenosylmethionine have at most a weak isolated impact on DNA methylation, as already demonstrated by Mandaviya et al. [4] and Dugué et al. [5].

Our study has several strengths. It is the first study which examined in three independent studies the intake of many food groups and subgroups for their association with DNA methylation. We harmonized the dietary intake data of KORA, LLS and TUK to ensure that same food classification scheme was applied. Residual confounding by energy intake was best considered by calculating food group residuals and using these in our models.

The analytic method to estimate the methylation level was similar across studies; the larger set of CpG sites measured in KORA was not considered here since the analyses were based on overlapping CpG sites across all studies. Our study also has limitations. We did not perform a food substitution model. Thus, we could not exclude the possibility that another food can act as a compensating mechanism. Also, since we have no gene expression data, conclusions about the effect of methylation change have to be confirmed in mechanistic studies. Additionally, we only had access to whole blood cells; therefore, we cannot draw any tissue-specific conclusions. Finally, there could be limited correlation of the same CpGs in the Illumina 450 k Chip used by TwinsUK and LLS and in the EPIC 850 k Chip used by KORA [64]. These results need replication to further clarify the association of food groups with white blood cell DNA methylation. As a fixed-effect model was chosen, extrapolating conclusions to different populations has to be done carefully. Although the random-effects meta-analysis more closely resembles the data reality than a fixed-effects analysis, because of the assumption of underlying distinct true means, the results should not be valued over the fixed-effects analysis, since an imprecise tau is included in our random-effects model [37]. We are aware of the debate around the focus on *p* values [65], but since we needed a threshold to decide if a CpG in this explorative study represents a meaningful finding, we deemed this the best fit. Due to the design of this study, we cannot draw conclusions regarding causality. Lastly, since dietary intake was assessed by FFQ’s (TUK, LLS) or a blended approach using repeated 24 h food list and an FFQ, exposure data may suffer from differential bias(including self-reporting bias).

## Conclusions

This study analyzed a broad range of different food groups and subgroups from three cohorts for their association with CpG methylation level. There were no significant associations for almost all vegetable or fruit food (sub-) groups. Rather, we observed interesting signals in food groups rich in fat, such as nuts and seeds, cream, butter, and plant oils. Some of the annotated genes seem to support the frequently observed effects of high-fat diets on DNA methylation in experimental studies. However, the results need replication in other cohorts with appropriate sample sizes to overcome some of the limitations present in this study.

## Supplementary Information

Below is the link to the electronic supplementary material.

Supplementary file1 (XLSX 16 KB)

Supplementary file2 (XLSX 13 KB)

Supplementary file3 (XLSX 19 KB)

Supplementary file4 (PDF 1073 KB)

Supplementary file5 (PDF 3114 KB)

## Data Availability

The informed consents given by KORA study participants do not cover data provision in public databases. However, data are available upon request from KORA-gen (http://www.helmholtz-muenchen.de/kora-gen). Data requests can be submitted online and are subject to approval by the KORA Board. LLS DNA methylation data are available upon request via the BIOS consortium (https://www.bbmri.nl/acquisition-use-analyze/bios). FFQ data is available upon request. Many of the data analyzed in TwinsUK is available through GEO GSE62992 and GSE121633. Additional individual-level data are not permitted to be shared or deposited due to the original consent given at the time of data collection. However, access to these data can be applied for through the TwinsUK data access committee. For information on access and how to apply http://twinsuk.ac.uk/resources-for-researchers/access-our-data/.

## Abbreviations

AHEI 2010: Alternate Healthy Eating Index 2010
BMI: Body-Mass-Index
DNMT: DNA methyltransferase
EWAS: Epigenome-wide association study
FFQ: Food frequency questionnaire
KORA: KORA FF4
LLS: Leiden Longevity Study
MDS: Mediterranean Diet Score
SI: Supplementary Information
TUK: TwinsUK
