# Supplementary material for: Pooled analysis of epigenome-wide association studies of food consumption in KORA, TwinsUK and LLS
Source: Eur J Nutr. 2022 Dec 26;62(3):1357–75. doi: 10.1007/s00394-022-03074-9 (PMC10030421; doi:10.1007/s00394-022-03074-9)
Supplement: Supplementary file 4 — Supplementary file4 (PDF 1073 KB) [file 394_2022_3074_MOESM4_ESM.pdf]

Article title: Pooled analysis of epigenome-wide association studies of food consumption in KORA, TwinsUK and LLS

Journal name: European Journal of Nutrition

Author names: Fabian Hellbach, Lucy Sinke, Ricardo Costeira, Sebastian-Edgar Baumeister, Marian Beekman, Panayiotis Louca, Emily R Leeming, Olatz Mompeo, Sarah Berry, Rory Wilson, Nina Wawro, Dennis Freuer, Hans Hauner, Annette Peters, Juliane Winkelmann, Wolfgang Koenig, Christa Meisinger, Melanie Waldenberger, Bas Heijmans, Eline Slagboom, Jordana T Bell, Jakob Linseisen

Corresponding Author: Fabian Hellbach[1,2]

Email: fabian.hellbach@med.uni-augsburg.de

[1]Institute for Medical Information Processing, Biometry, and Epidemiology; Medical Faculty; Ludwig-Maximilian University Munich; Marchioninistr. 15, 81377 Munich, Germany

[2]Epidemiology; Faculty of Medicine; University of Augsburg; University Hospital Augsburg; Stenglinstraße 2, 86156 Augsburg; Germany

Caption: Heterogeneity statistics for every food group analyzed. Shown are P-value distribution of the Q-Heterogeneity measure (left), I^2 values (right) and estimated tau-values (bottom)

Fig 77

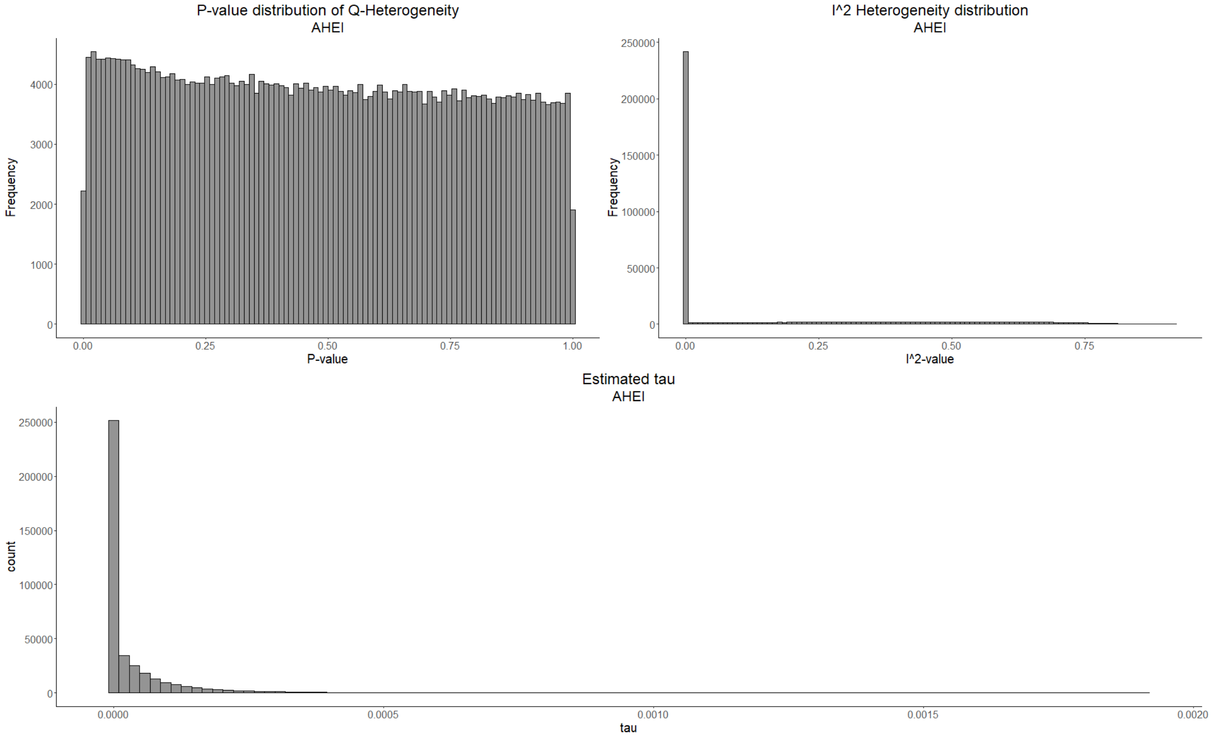
P-value distribution of Q-Heterogeneity
AHEI
Frequency
P-value
I^2 Heterogeneity distribution
AHEI
Frequency
I^2-value
Estimated tau
AHEI
count
tau

Fig 78

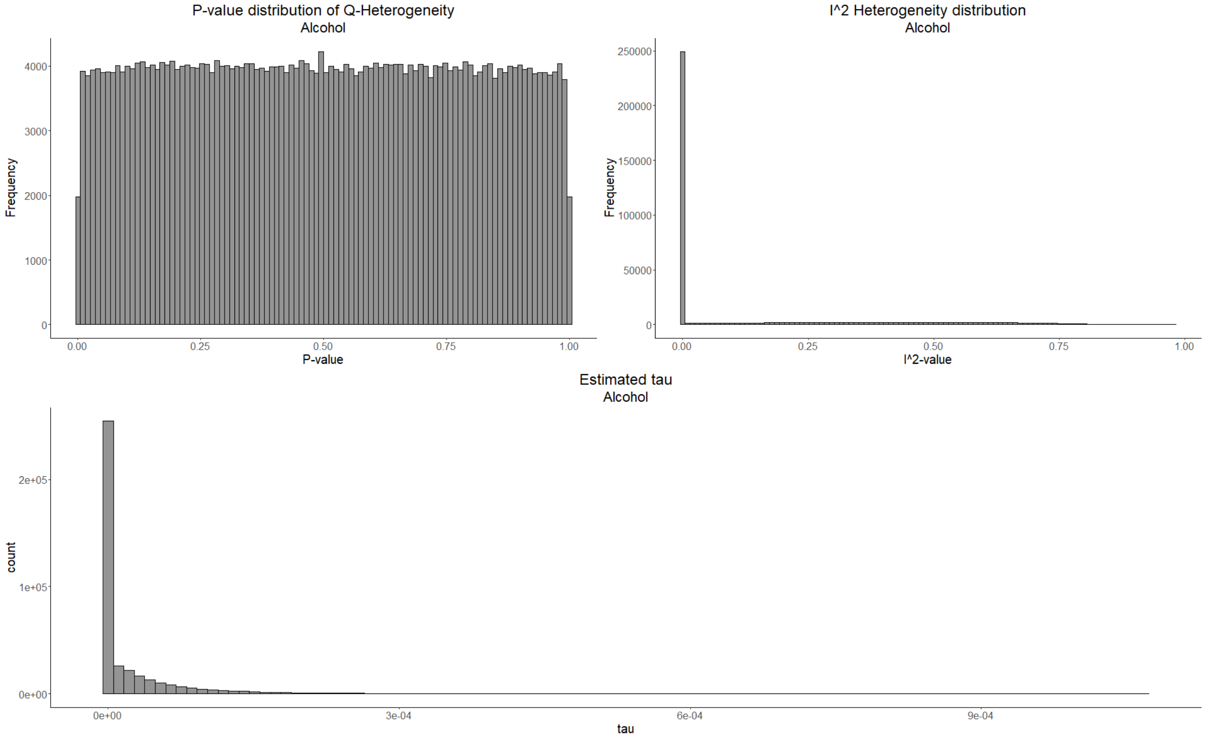
P-value distribution of Q-Heterogeneity
Alcohol
Frequency
P-value
I^2 Heterogeneity distribution
Alcohol
Frequency
I^2-value
Estimated tau
Alcohol
count
tau

Fig 79

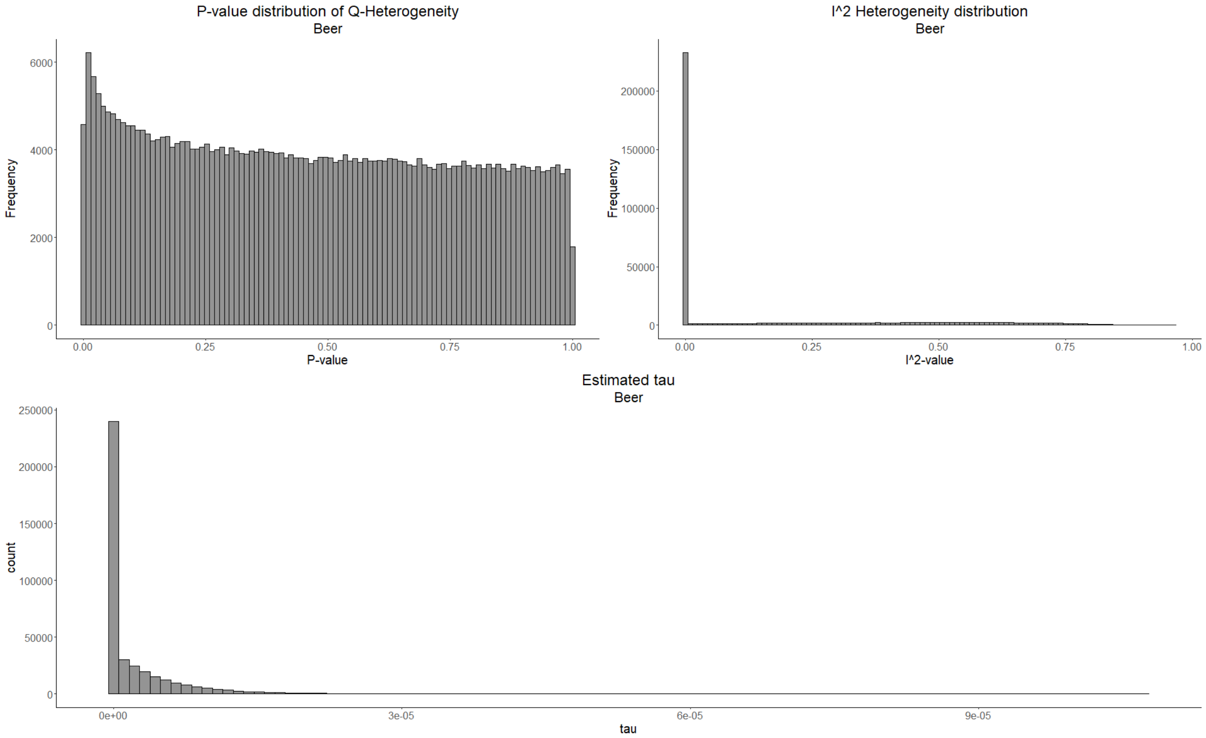

P-value distribution of Q-Heterogeneity
Beer
Frequency
P-value
I^2 Heterogeneity distribution
Beer
Frequency
I^2-value
Estimated tau
Beer
count
tau

Fig 80

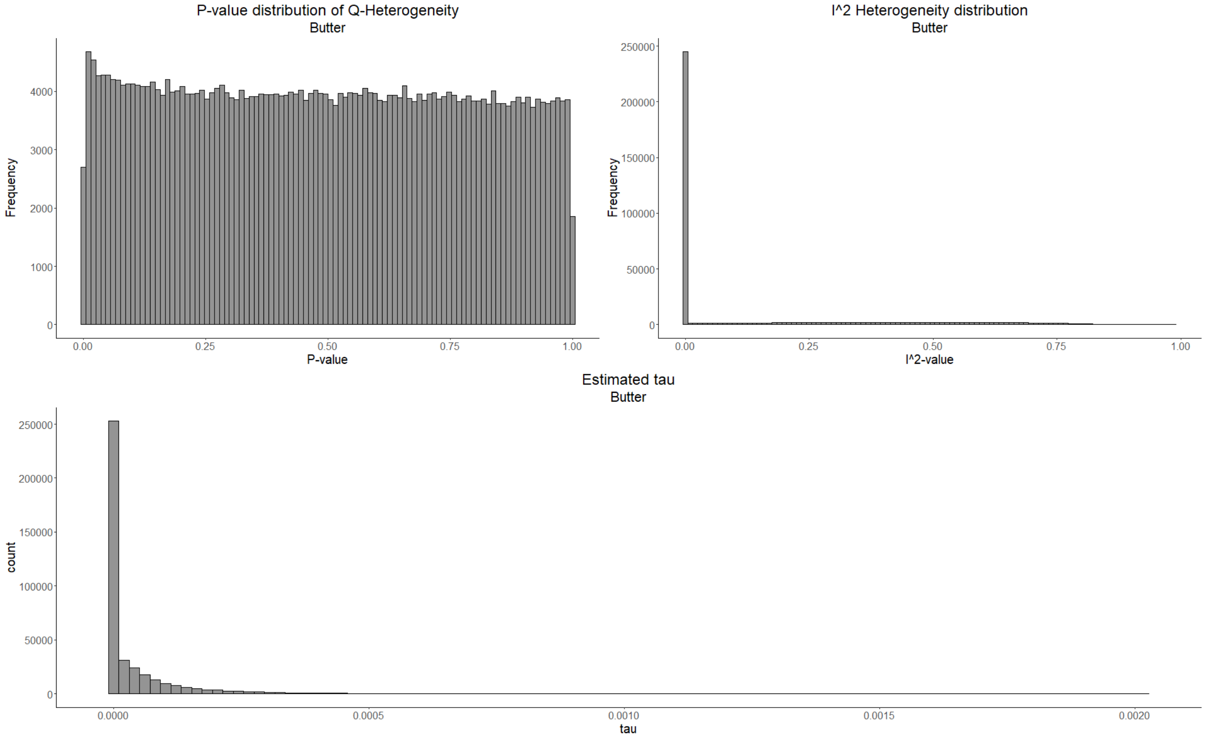

P-value distribution of Q-Heterogeneity
Butter
Frequency
P-value
I^2 Heterogeneity distribution
Butter
Frequency
I^2-value
Estimated tau
Butter
count
tau

Fig 81

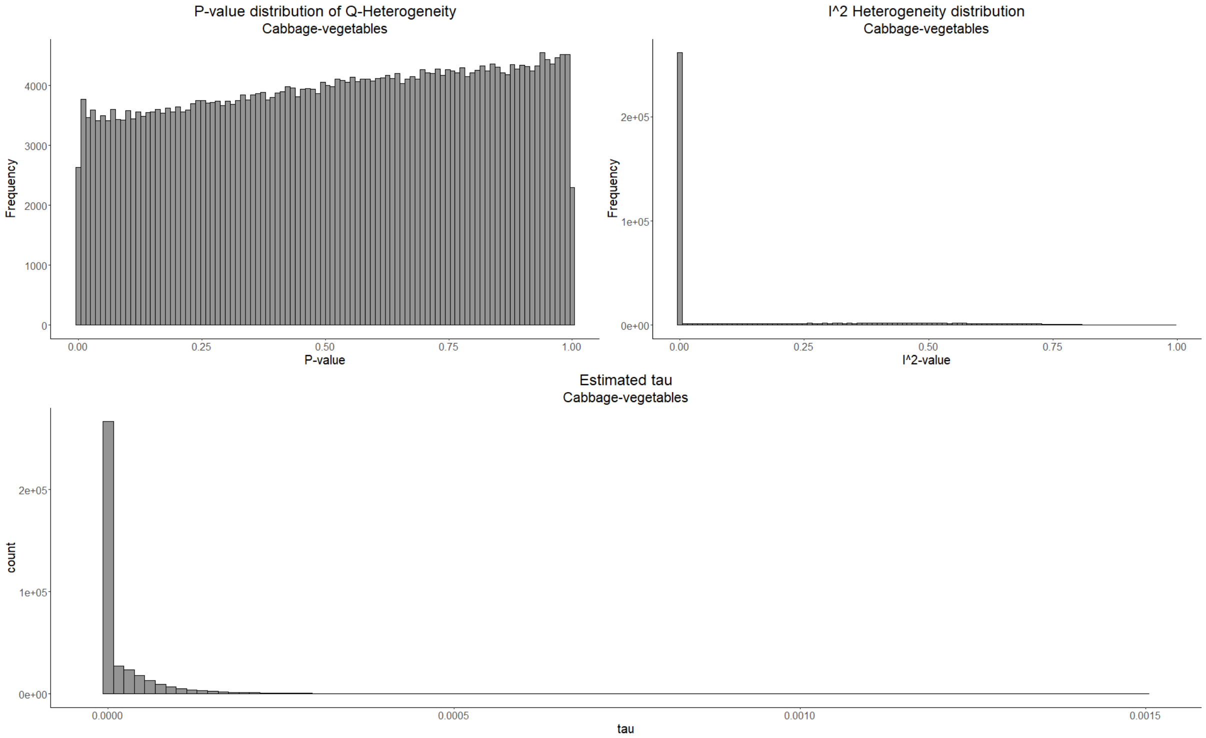
P-value distribution of Q-Heterogeneity
Cabbage-vegetables
Frequency
P-value
I^2 Heterogeneity distribution
Cabbage-vegetables
Frequency
I^2-value
Estimated tau
Cabbage-vegetables
count
tau

Fig 82

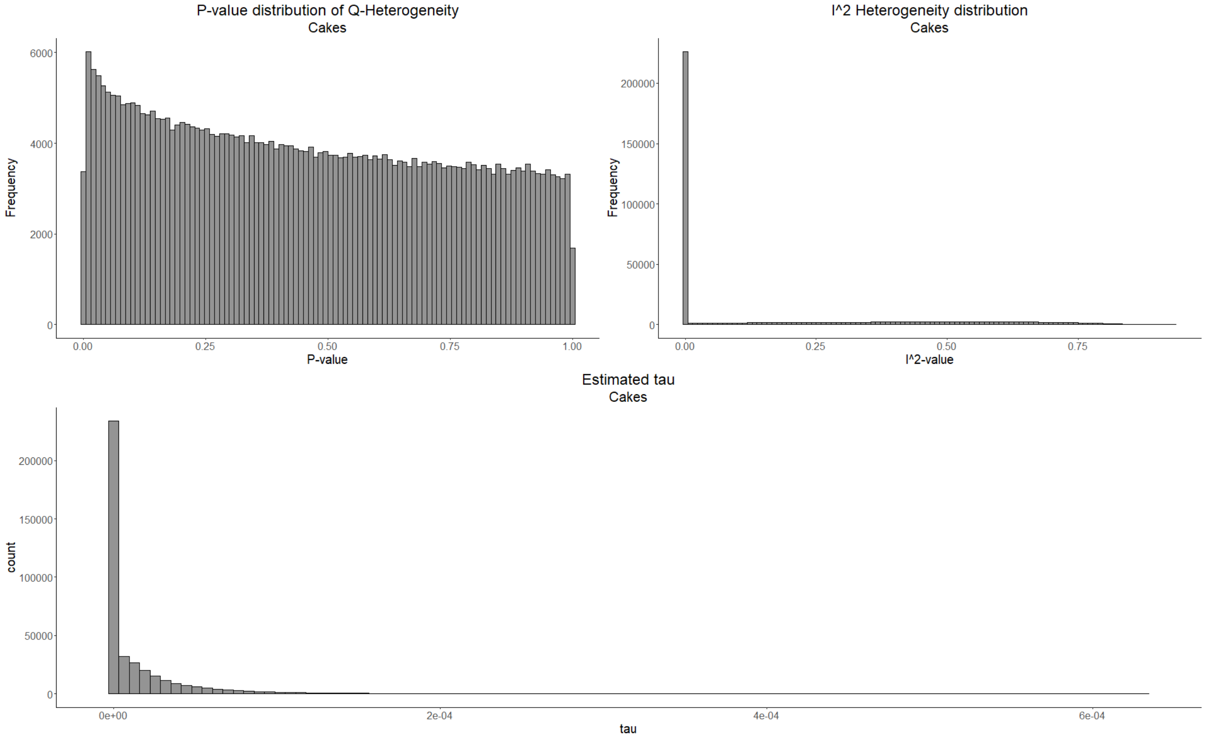
P-value distribution of Q-Heterogeneity
Cakes
Frequency
P-value
I^2 Heterogeneity distribution
Cakes
Frequency
I^2-value
Estimated tau
Cakes
count
tau

Fig 83

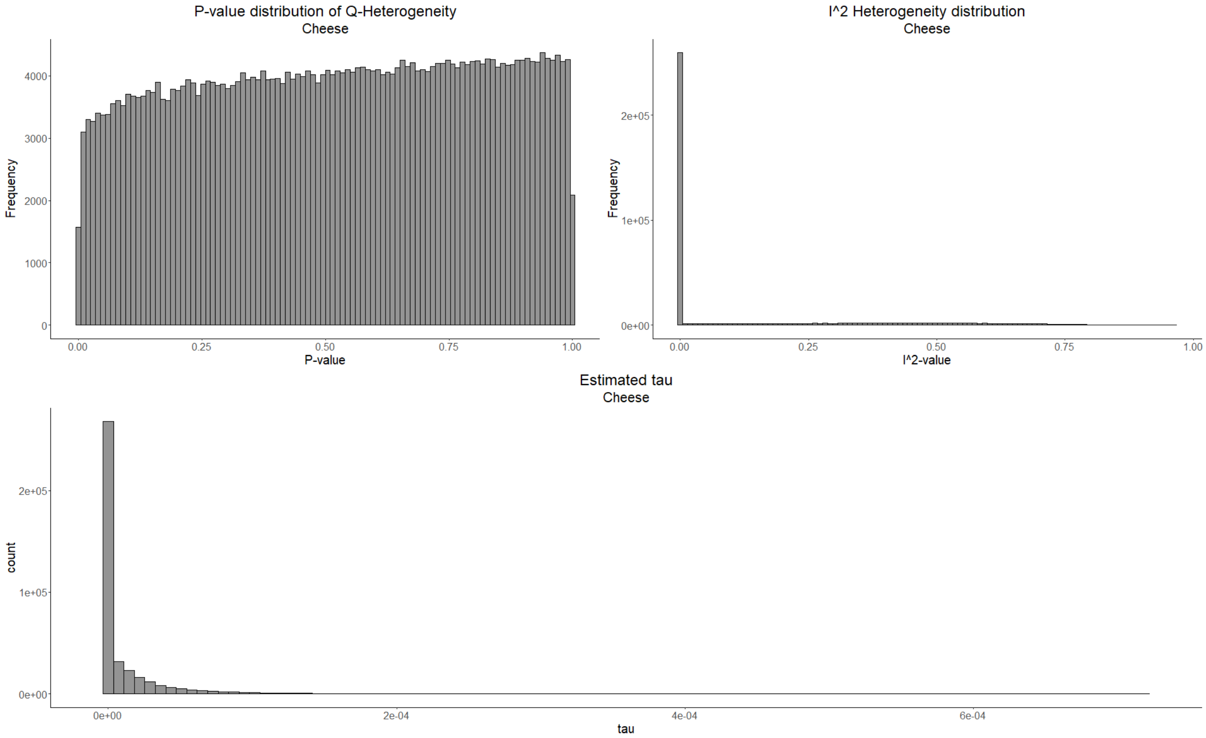
P-value distribution of Q-Heterogeneity
Cheese
Frequency
P-value
I^2 Heterogeneity distribution
Cheese
Frequency
I^2-value
Estimated tau
Cheese
count
tau

Fig 84

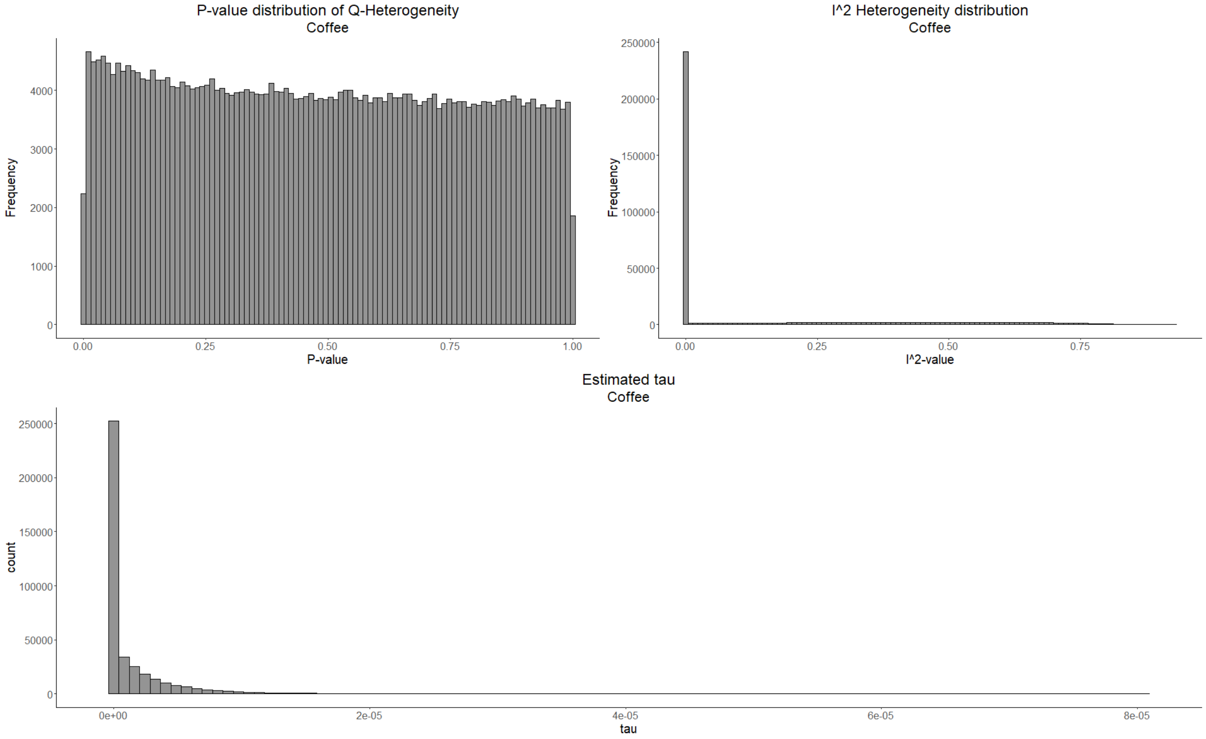
P-value distribution of Q-Heterogeneity
Coffee
Frequency
P-value
I^2 Heterogeneity distribution
Coffee
Frequency
I^2-value
Estimated tau
Coffee
count
tau

Fig 85

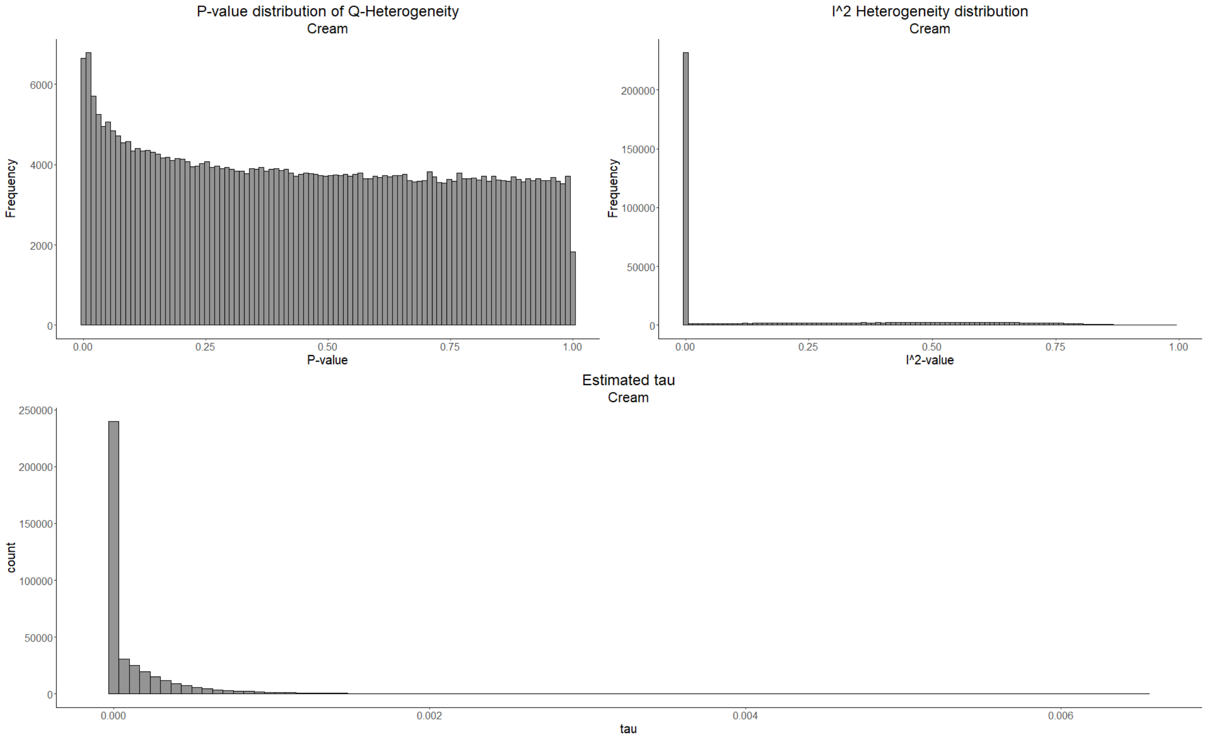
P-value distribution of Q-Heterogeneity
Cream
Frequency
P-value
I^2 Heterogeneity distribution
Cream
Frequency
I^2-value
Estimated tau
Cream
count
tau

Fig 86

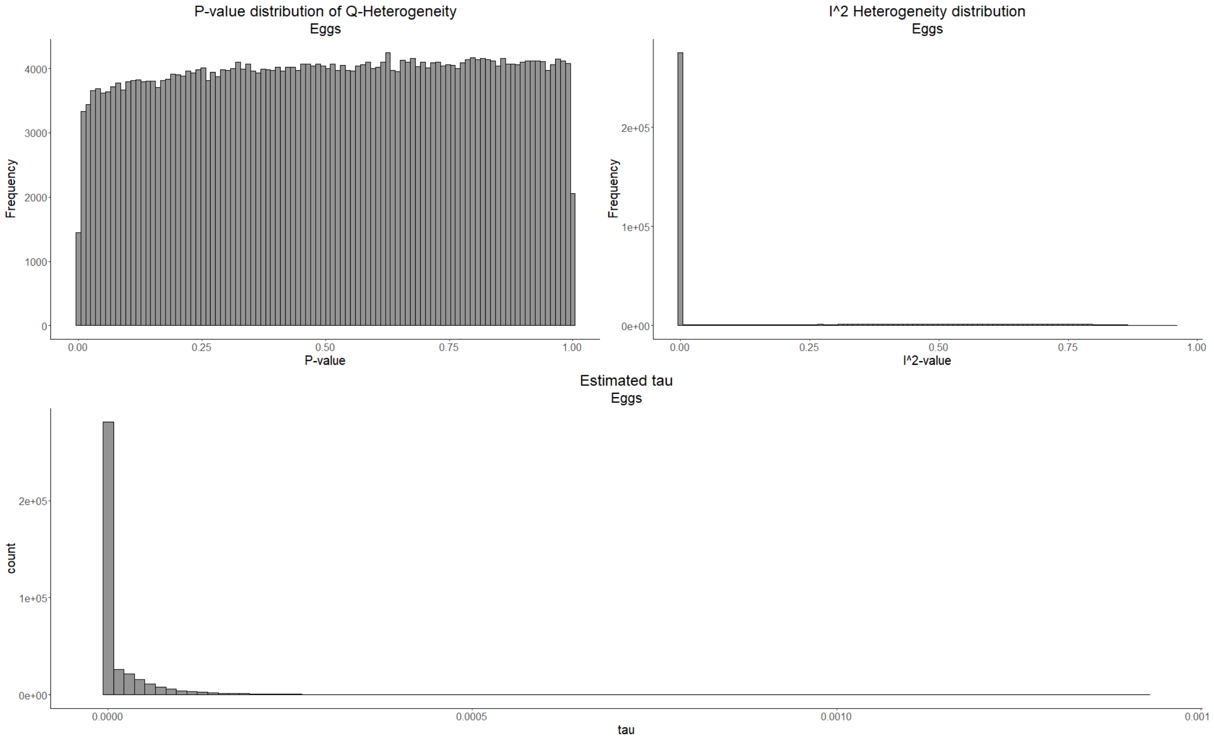
P-value distribution of Q-Heterogeneity
Eggs
Frequency
P-value
I^2 Heterogeneity distribution
Eggs
Frequency
I^2-value
Estimated tau
Eggs
count
tau

Fig 87

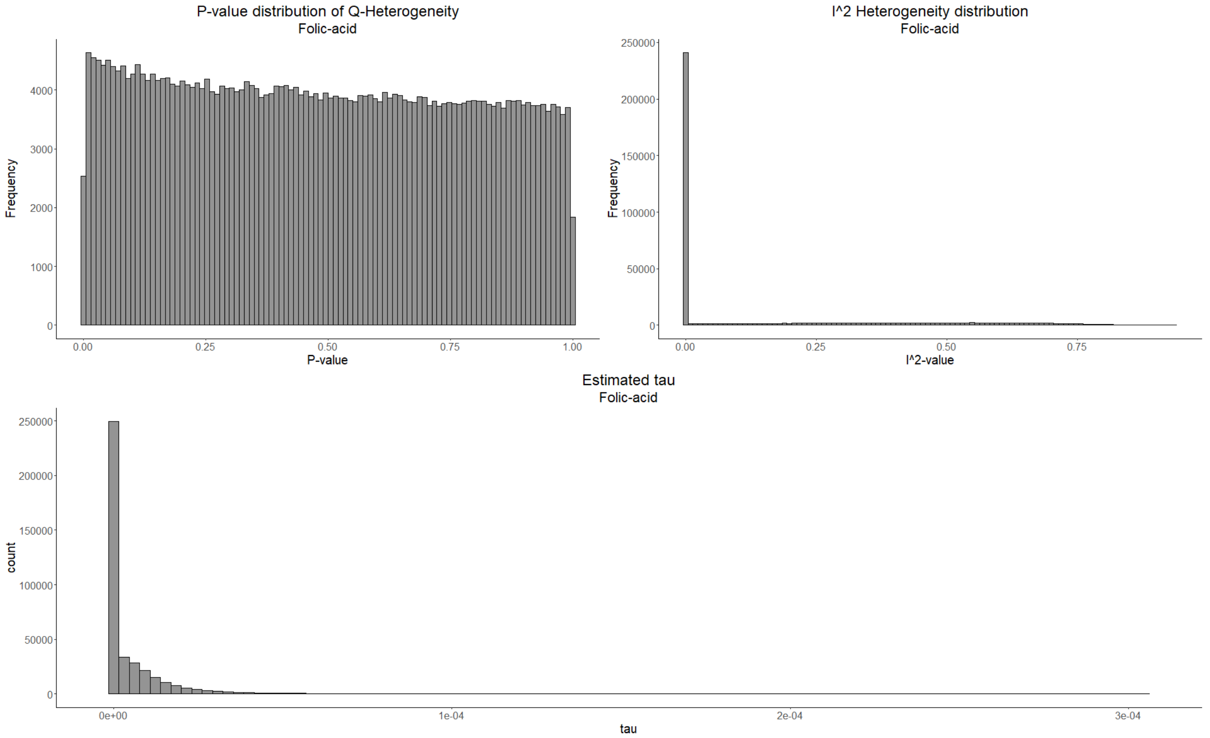

P-value distribution of Q-Heterogeneity
Folic-acid
Frequency
P-value
I^2 Heterogeneity distribution
Folic-acid
Frequency
I^2-value
Estimated tau
Folic-acid
count
tau

Fig 88

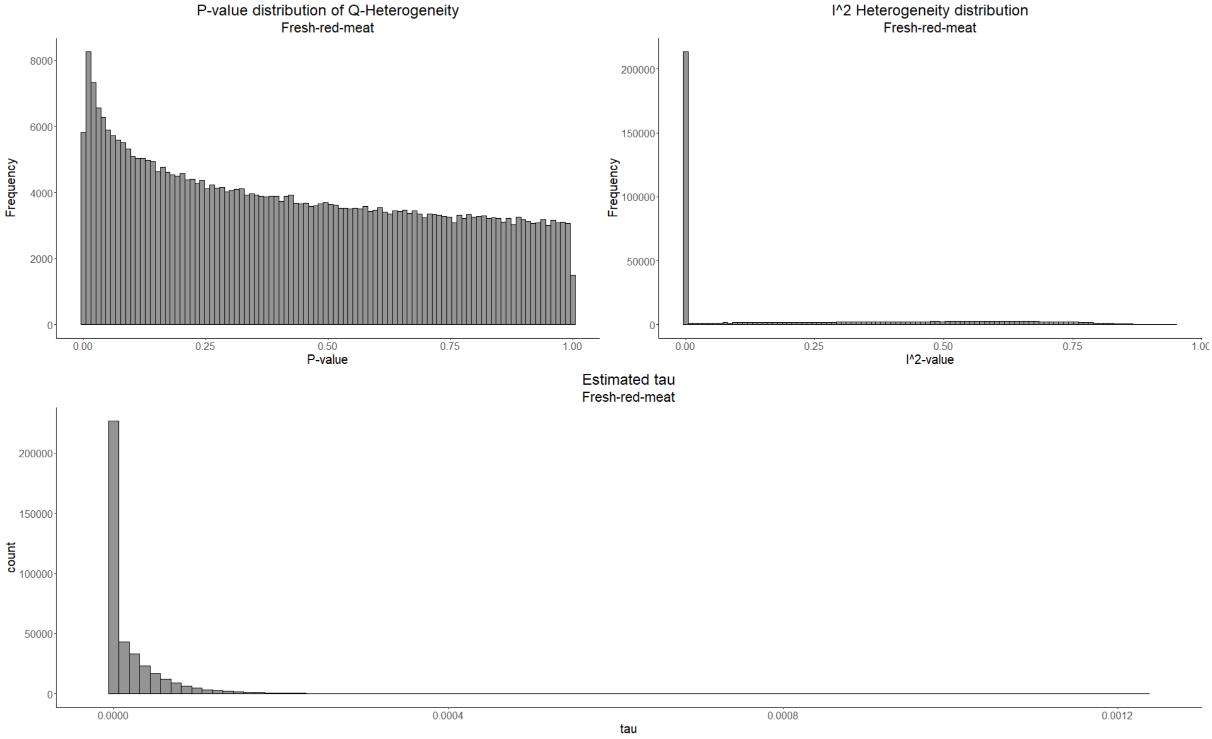

P-value distribution of Q-Heterogeneity
Fresh-red-meat
Frequency
P-value
I^2 Heterogeneity distribution
Fresh-red-meat
Frequency
I^2-value
Estimated tau
Fresh-red-meat
count
tau

Fig 89

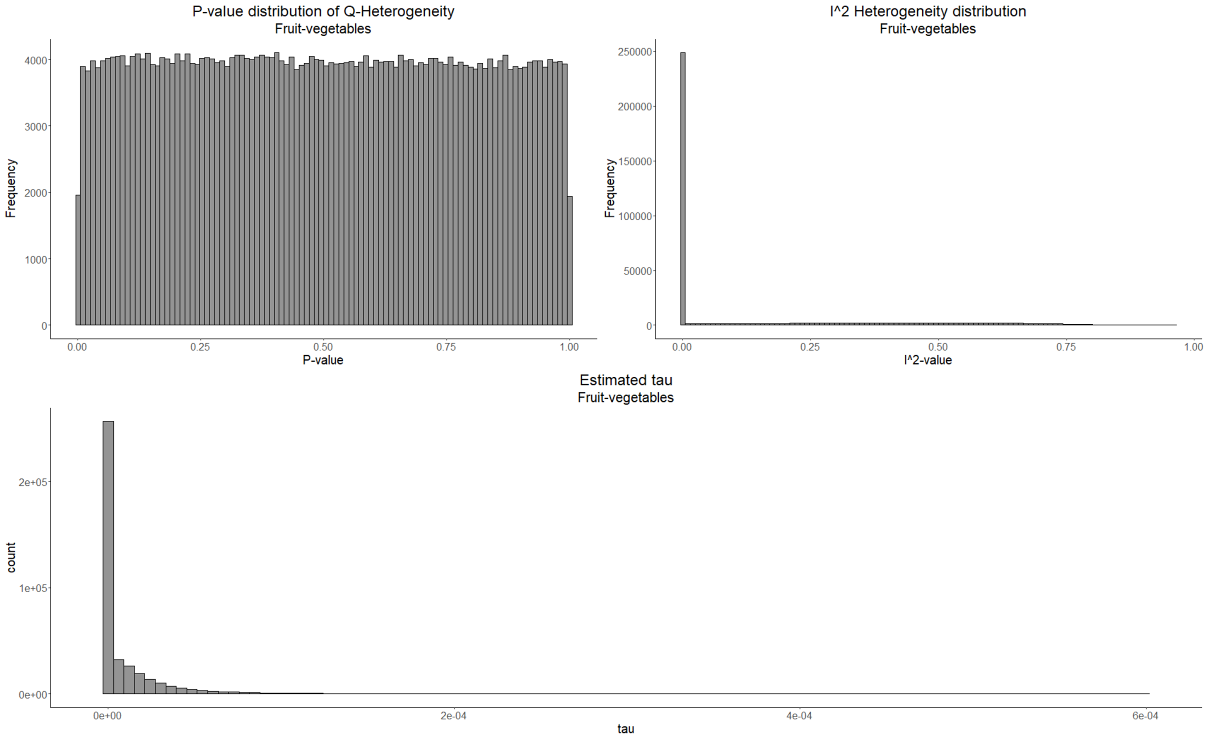
P-value distribution of Q-Heterogeneity
Fruit-vegetables
Frequency
P-value
0.00
0.25
0.50
0.75
1.00
0
1000
2000
3000
4000
I^2 Heterogeneity distribution
Fruit-vegetables
Frequency
I^2-value
0
50000
100000
150000
200000
250000
Estimated tau
Fruit-vegetables
count
tau
0e+00
1e+05
2e+05
2e-04
4e-04
6e-04

Fig 90

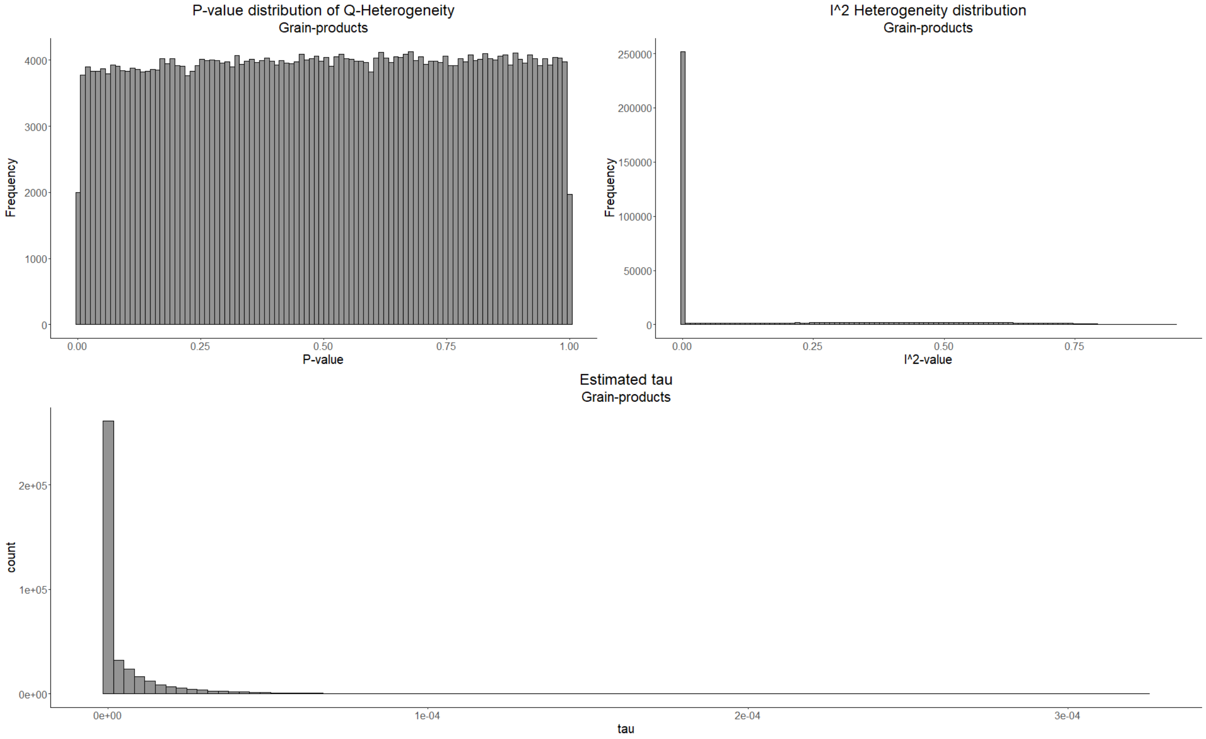
P-value distribution of Q-Heterogeneity
Grain-products
Frequency
P-value
0.00
0.25
0.50
0.75
1.00
0
1000
2000
3000
4000
I^2 Heterogeneity distribution
Grain-products
Frequency
I^2-value
0
50000
100000
150000
200000
250000
Estimated tau
Grain-products
count
tau
0e+00
1e+05
2e+05
1e-04
2e-04
3e-04

Fig 91

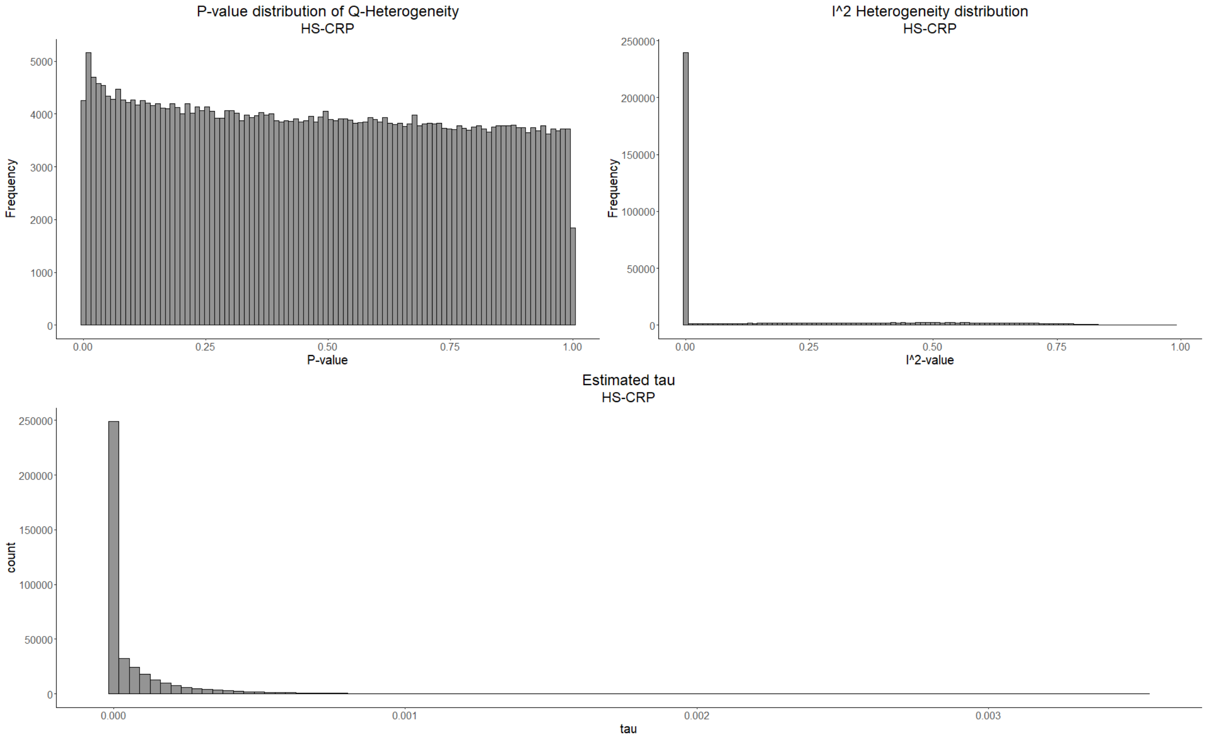
P-value distribution of Q-Heterogeneity
HS-CRP
Frequency
P-value
I^2 Heterogeneity distribution
HS-CRP
Frequency
I^2-value
Estimated tau
HS-CRP
count
tau

Fig 92

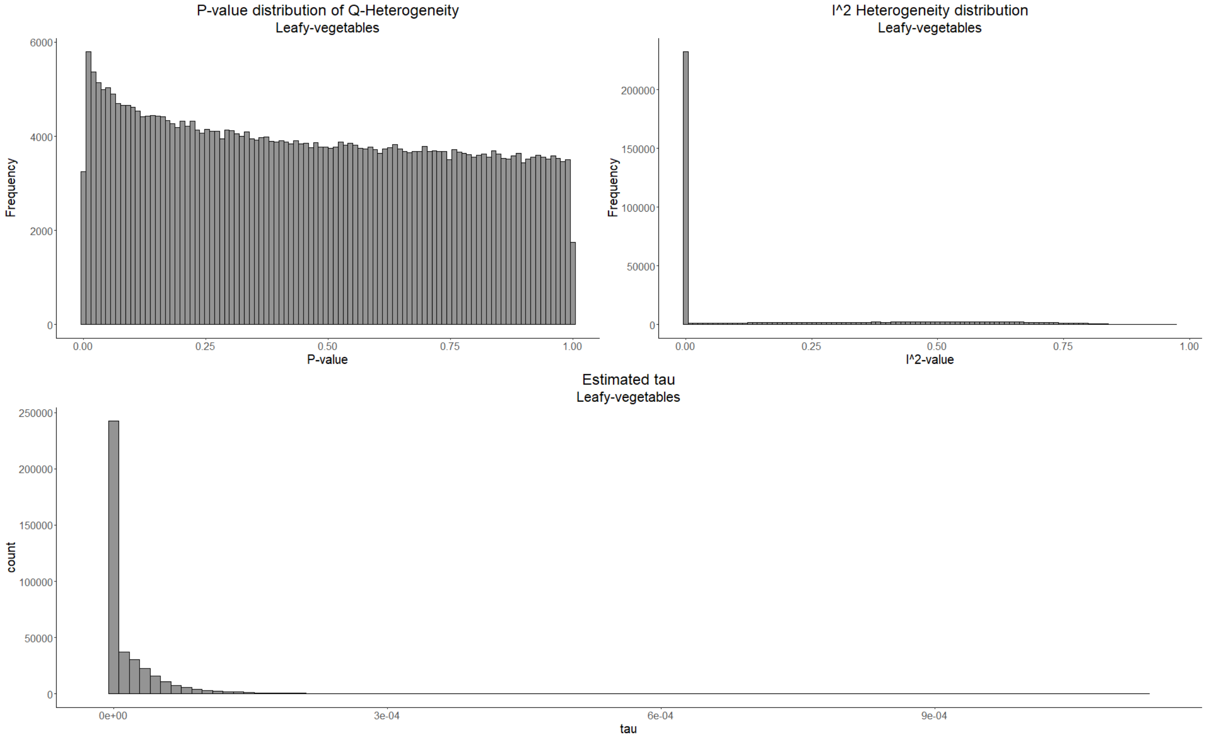
P-value distribution of Q-Heterogeneity
Leafy-vegetables
Frequency
P-value
I^2 Heterogeneity distribution
Leafy-vegetables
Frequency
I^2-value
Estimated tau
Leafy-vegetables
count
tau

Fig 93

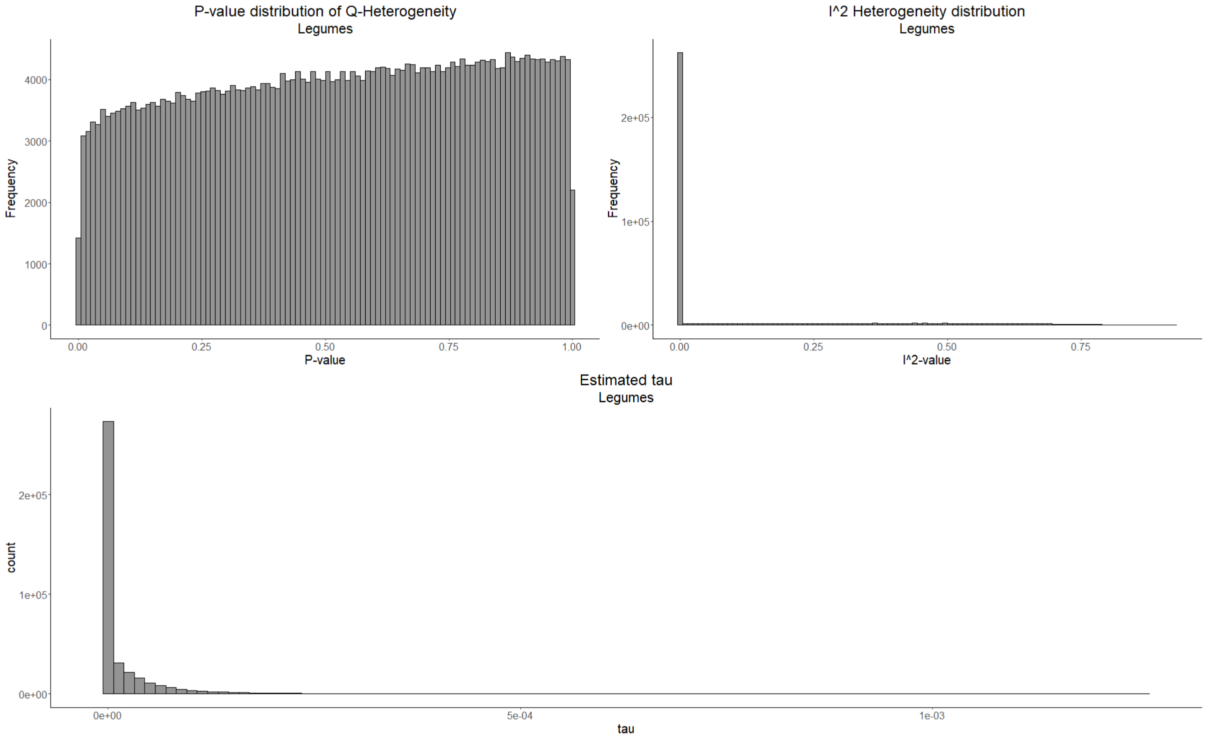
P-value distribution of Q-Heterogeneity
Legumes
Frequency
0
1000
2000
3000
4000
0.00
0.25
0.50
0.75
1.00
P-value
I^2 Heterogeneity distribution
Legumes
Frequency
0e+00
1e+05
2e+05
0.00
0.25
0.50
0.75
I^2-value
Estimated tau
Legumes
count
0e+00
1e+05
2e+05
0e+00
5e-04
1e-03
tau

Fig 94

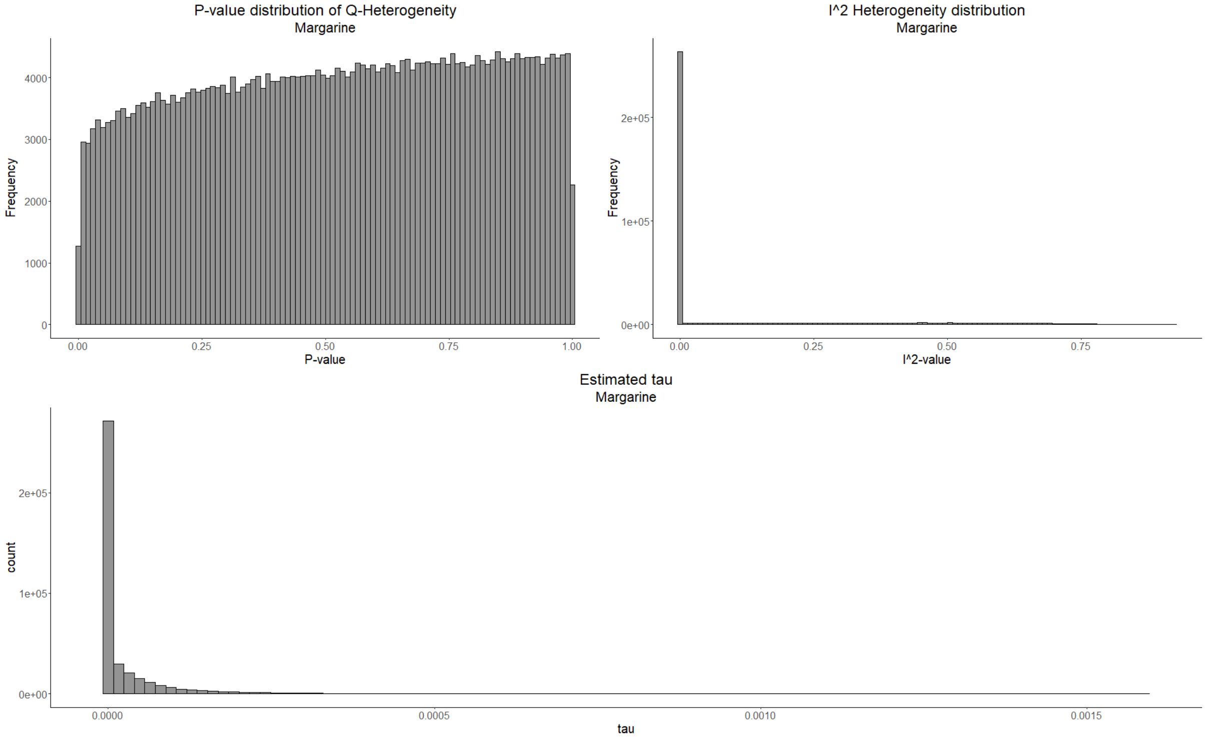
P-value distribution of Q-Heterogeneity
Margarine
Frequency
0
1000
2000
3000
4000
0.00
0.25
0.50
0.75
1.00
P-value
I^2 Heterogeneity distribution
Margarine
Frequency
0e+00
1e+05
2e+05
0.00
0.25
0.50
0.75
I^2-value
Estimated tau
Margarine
count
0e+00
1e+05
2e+05
0.0000
0.0005
0.0010
0.0015
tau

Fig 95

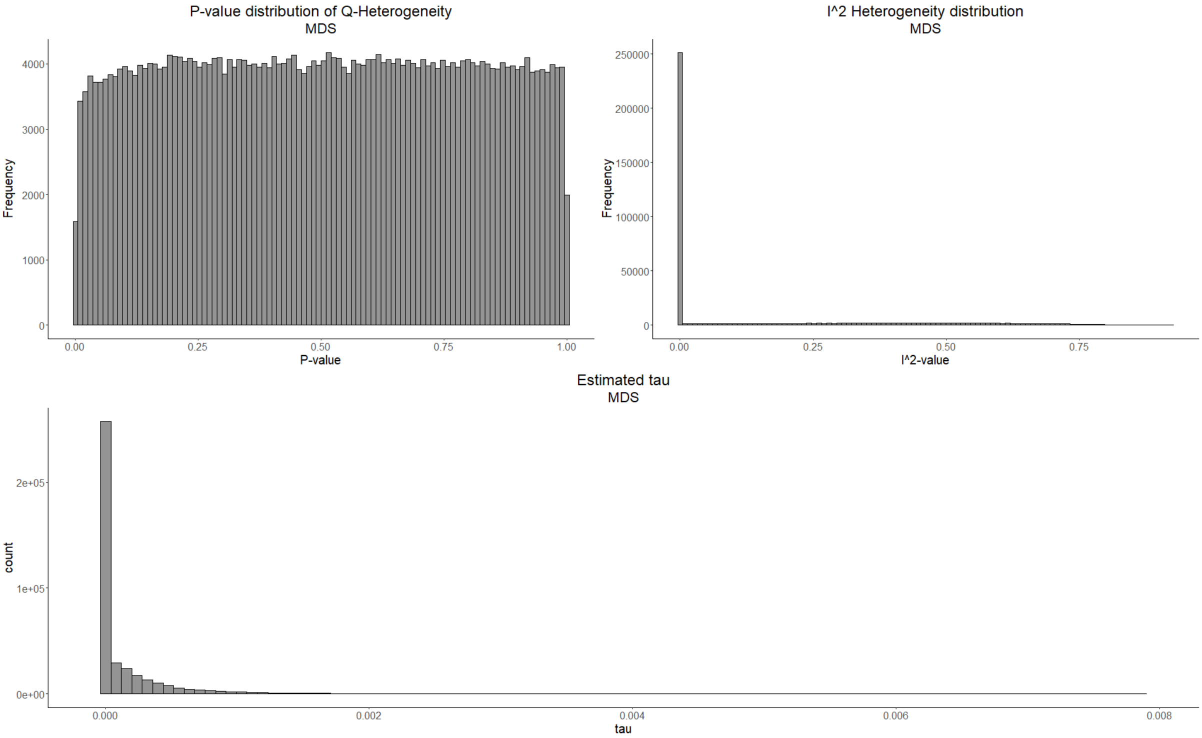

P-value distribution of Q-Heterogeneity
MDS
Frequency
P-value
I^2 Heterogeneity distribution
MDS
Frequency
I^2-value
Estimated tau
MDS
count
tau

Fig 96

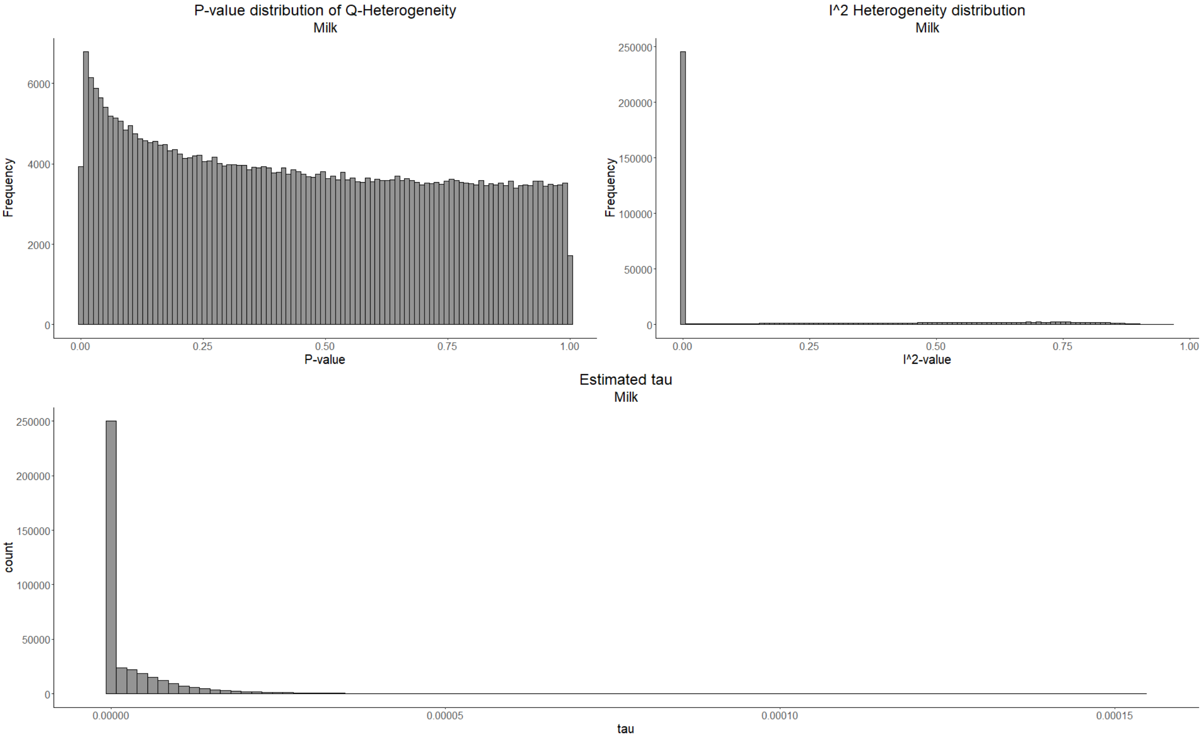

P-value distribution of Q-Heterogeneity
Milk
Frequency
P-value
I^2 Heterogeneity distribution
Milk
Frequency
I^2-value
Estimated tau
Milk
count
tau

Fig 97

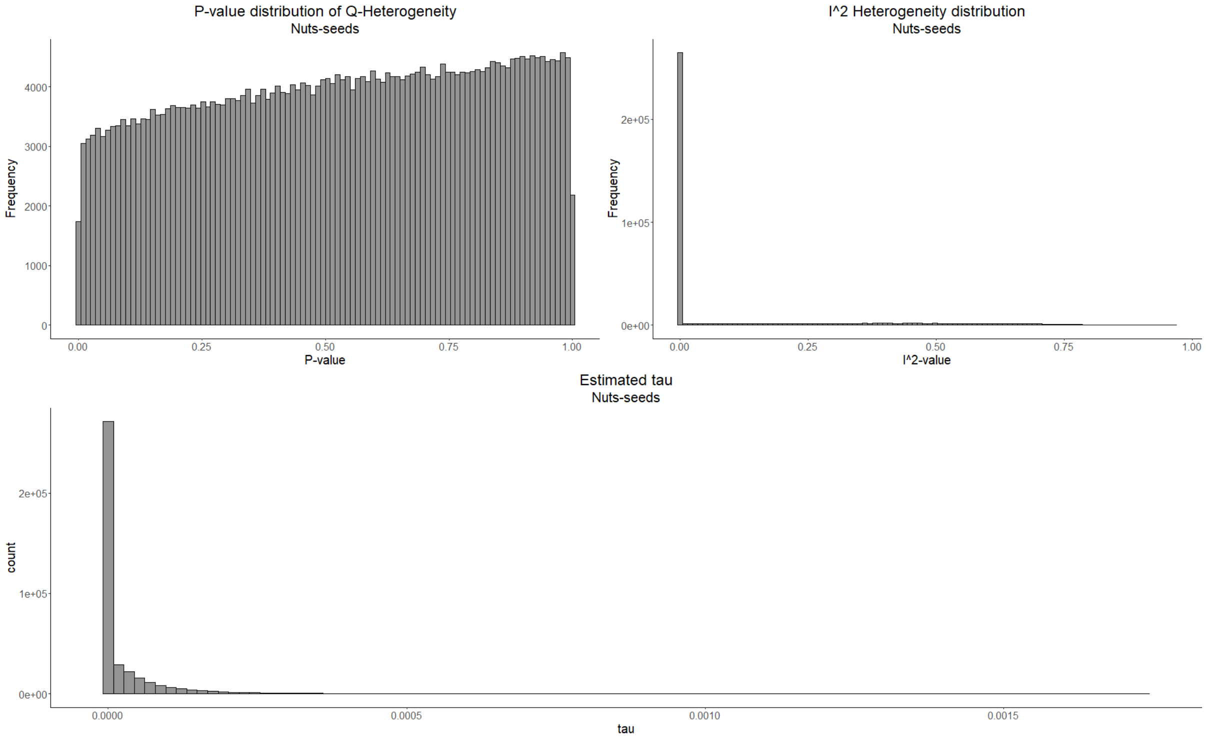
P-value distribution of Q-Heterogeneity
Nuts-seeds
Frequency
P-value
I^2 Heterogeneity distribution
Nuts-seeds
Frequency
I^2-value
Estimated tau
Nuts-seeds
count
tau

Fig 98

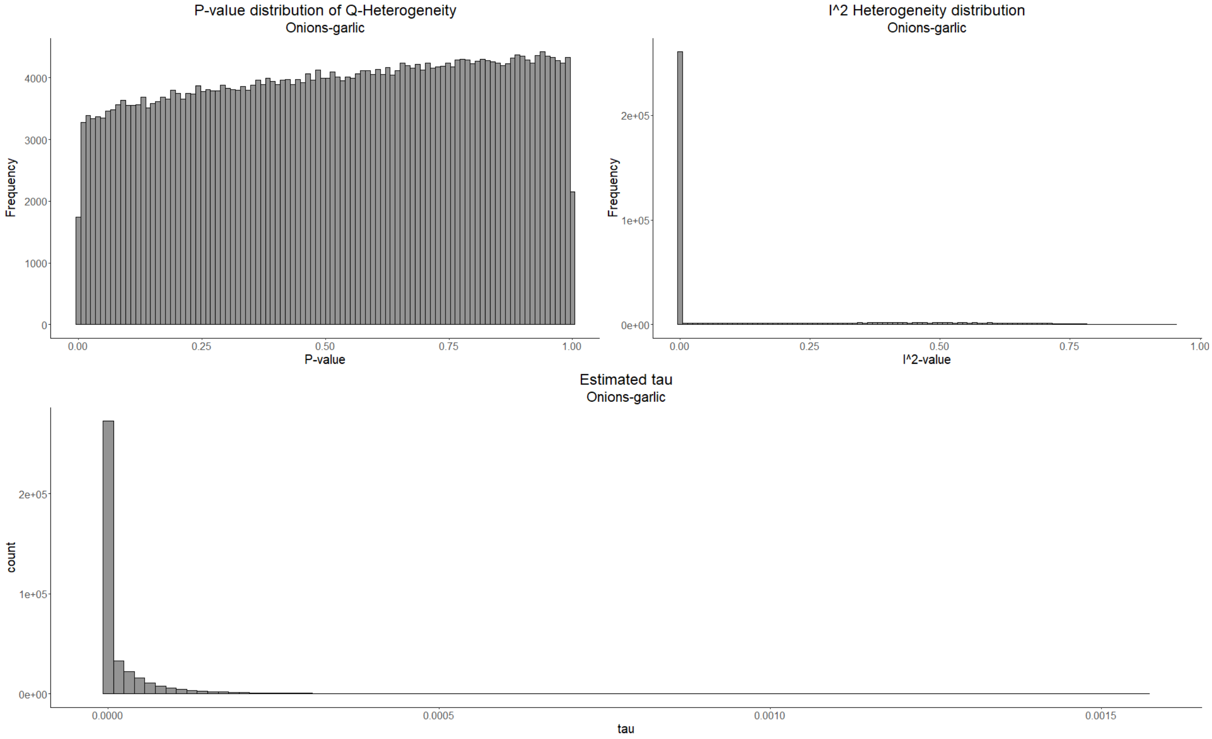
P-value distribution of Q-Heterogeneity
Onions-garlic
Frequency
P-value
I^2 Heterogeneity distribution
Onions-garlic
Frequency
I^2-value
Estimated tau
Onions-garlic
count
tau

Fig 99

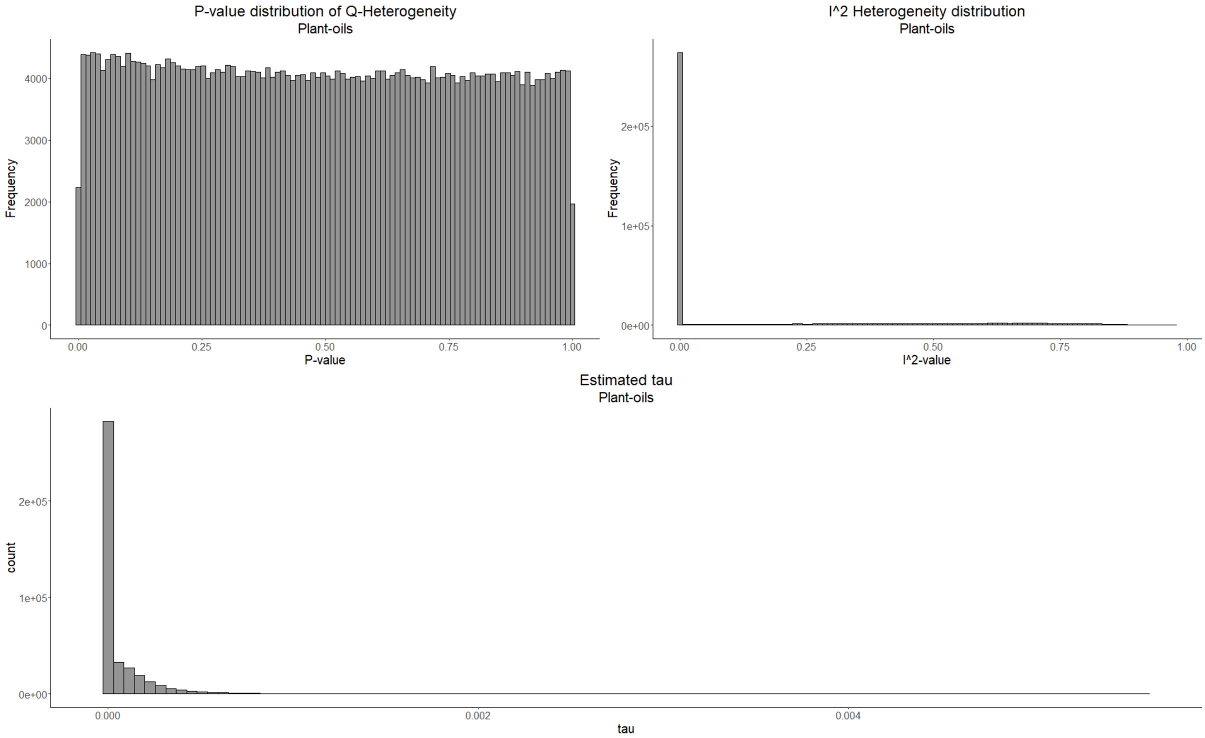

Fig 100

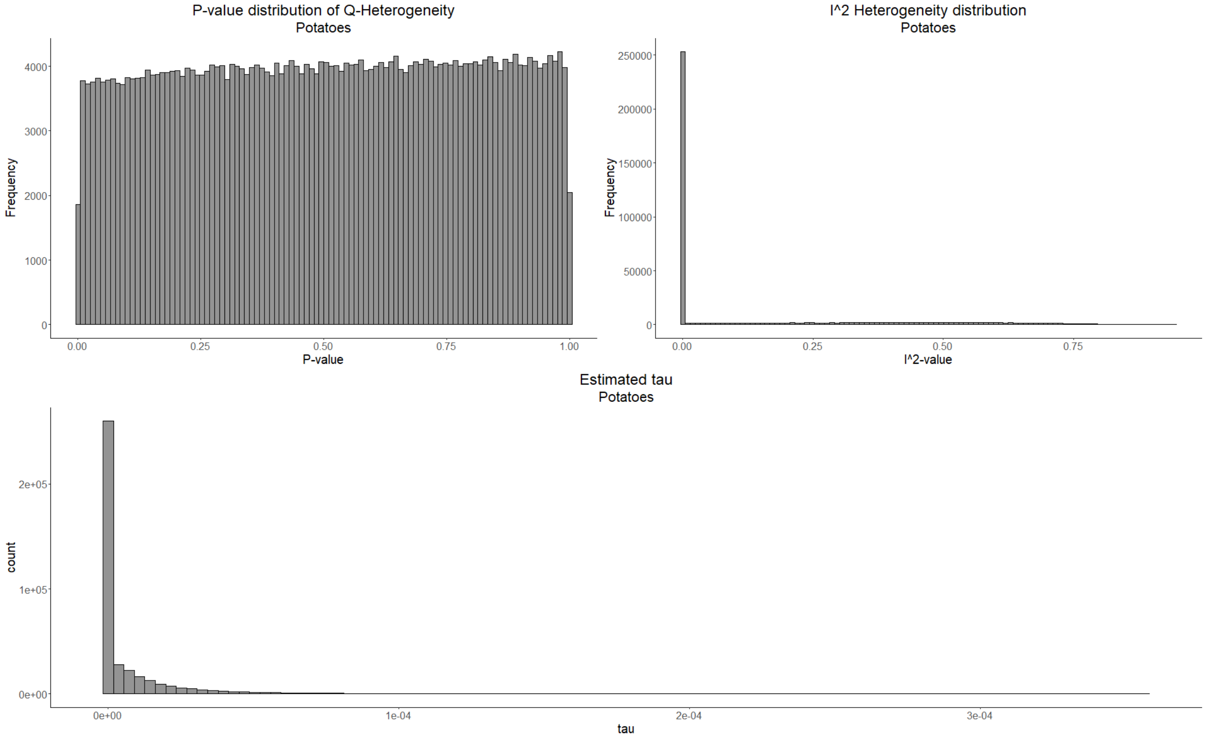

Fig 101

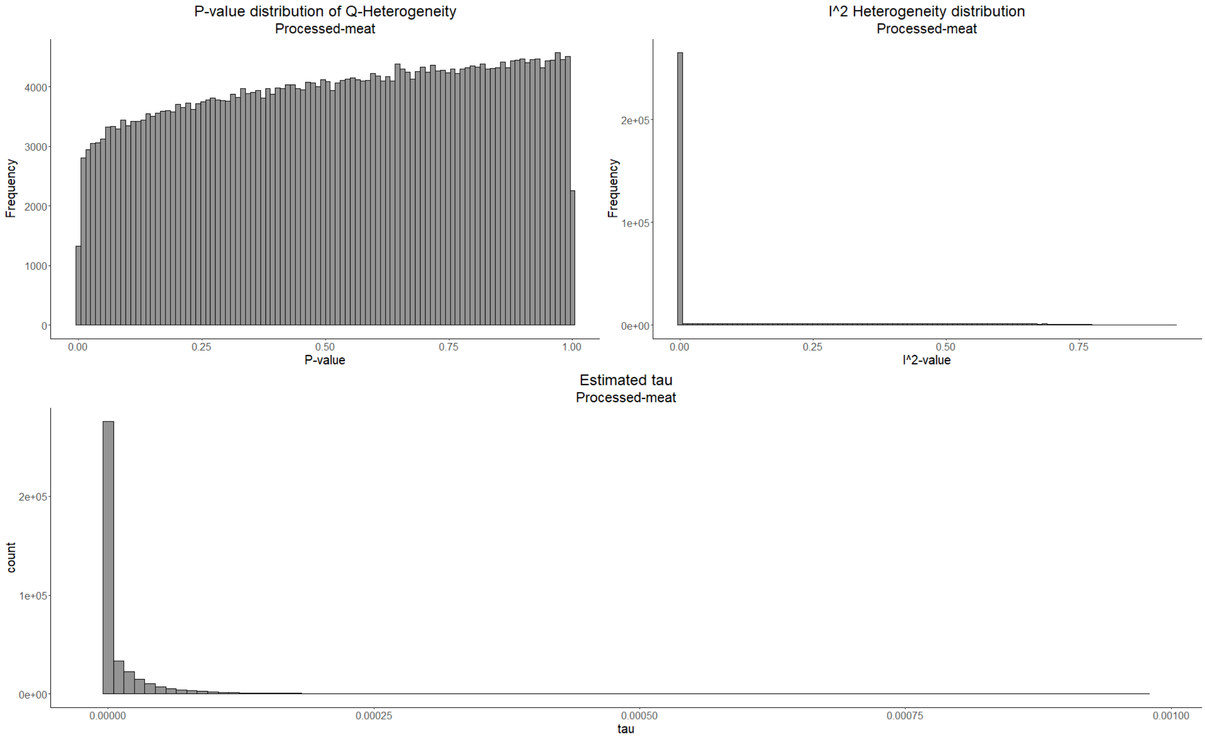

Fig 102

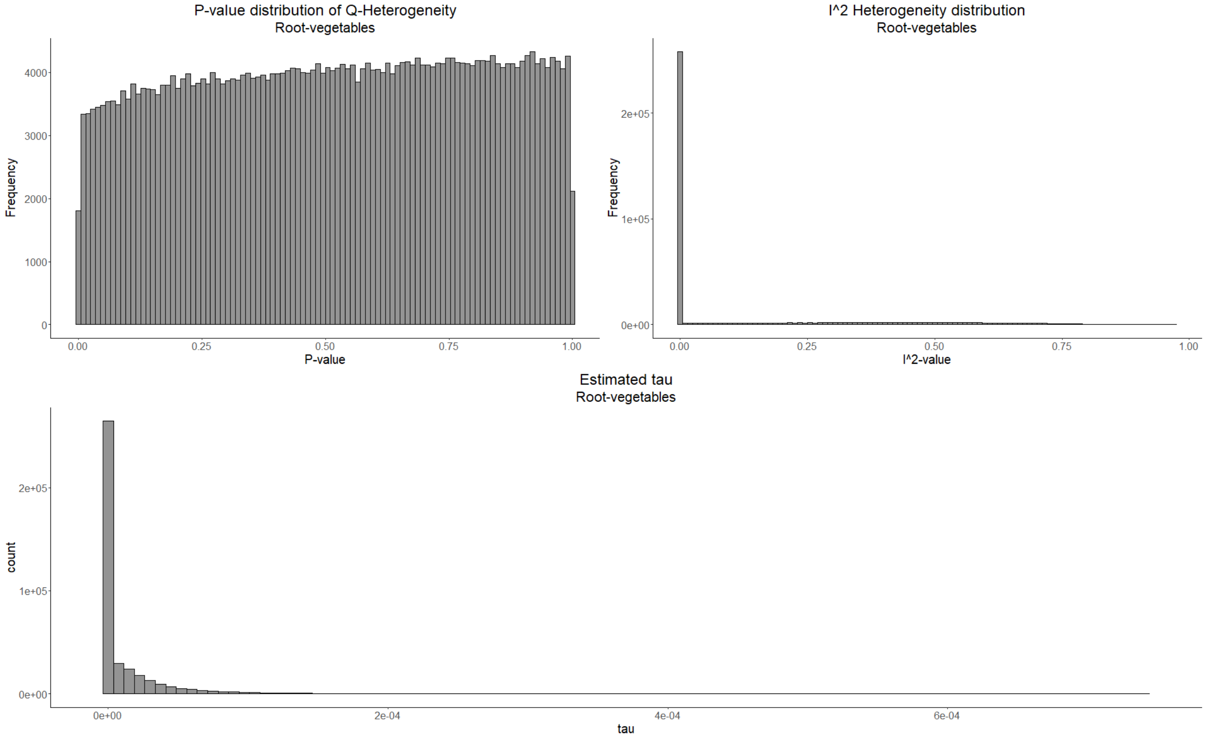

Fig 103

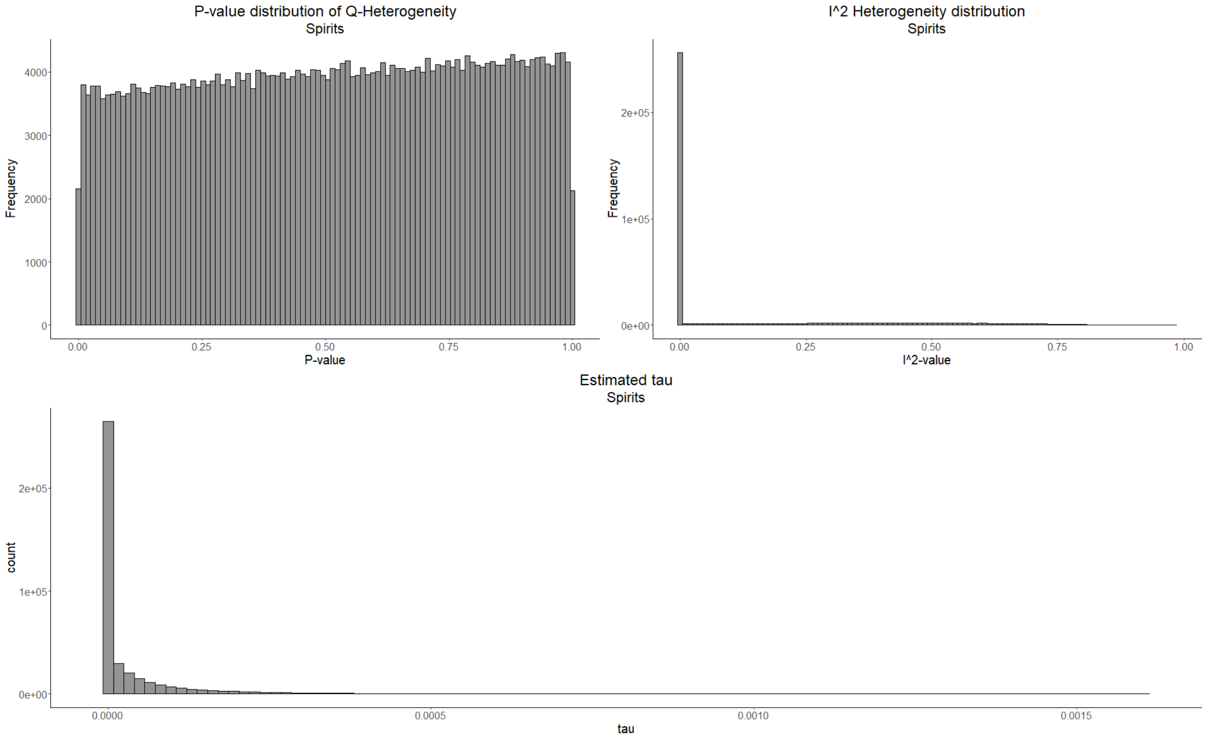

Fig 104

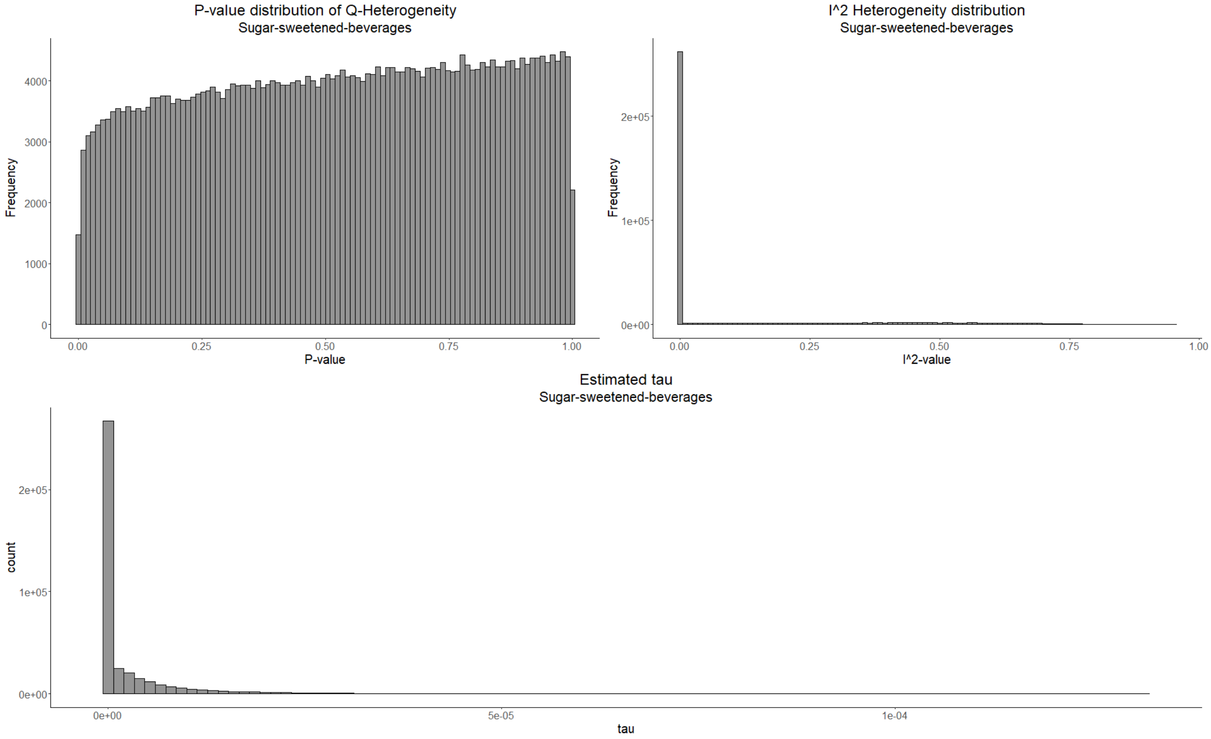

Fig 105

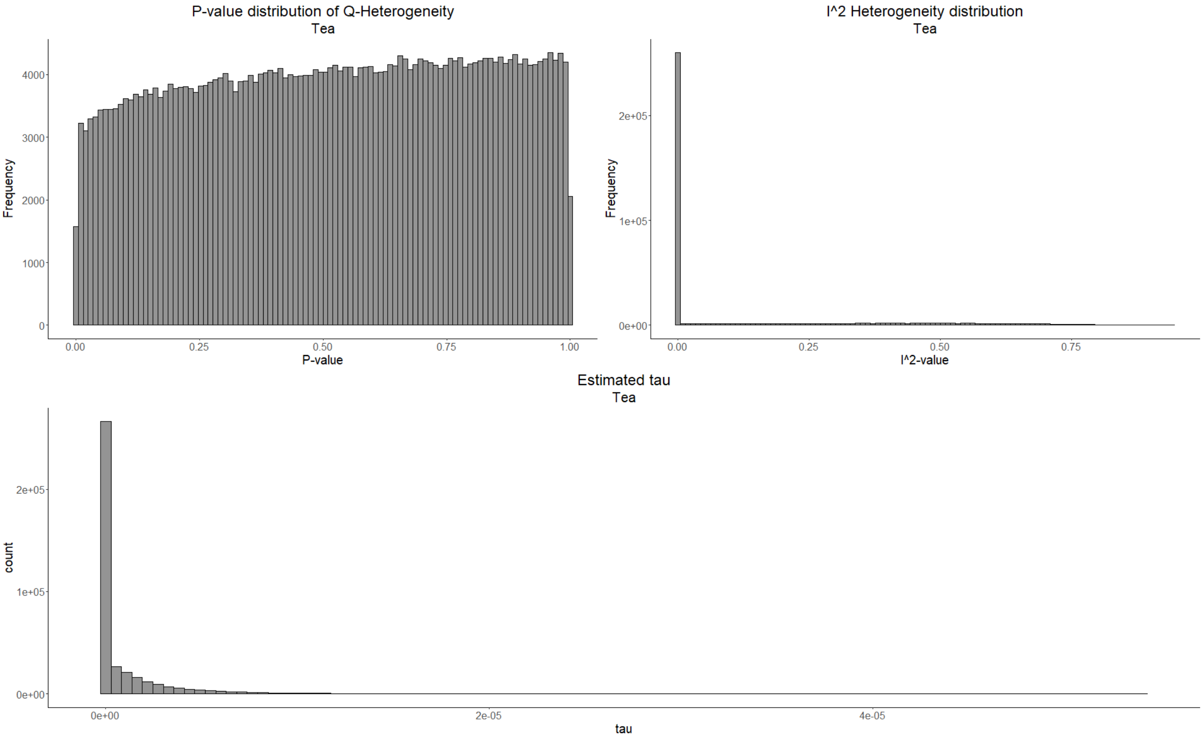

Fig 106

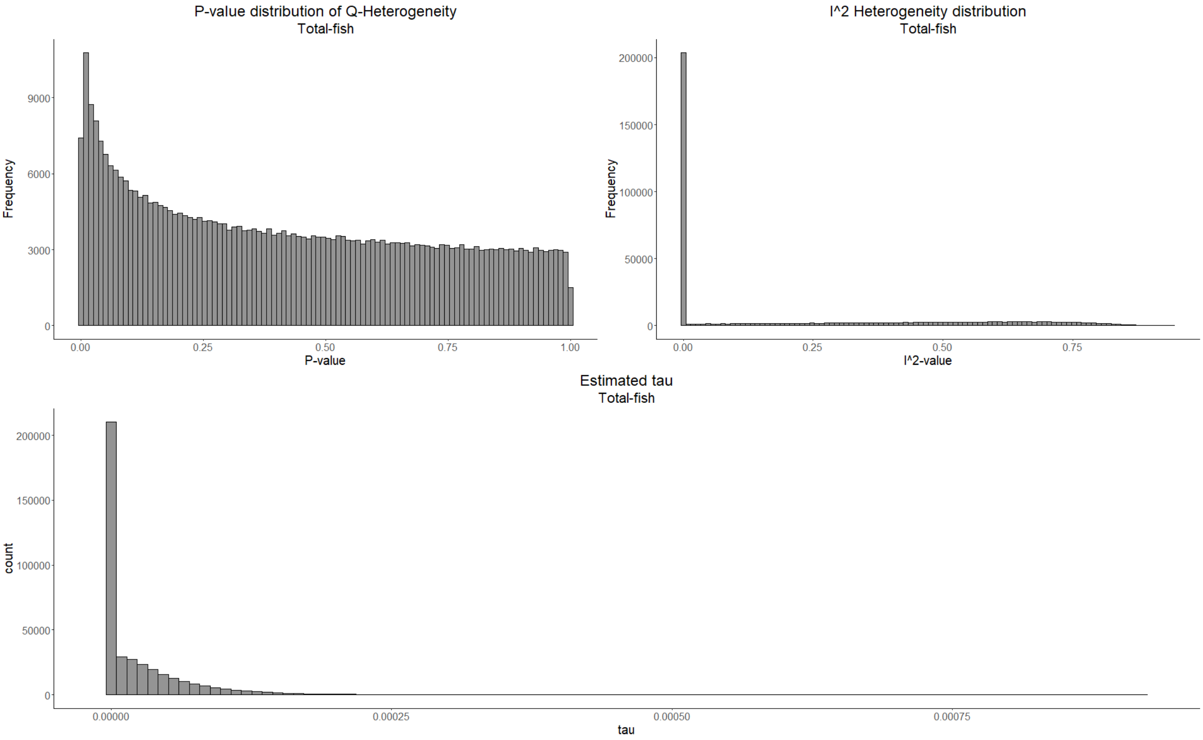

Fig 107

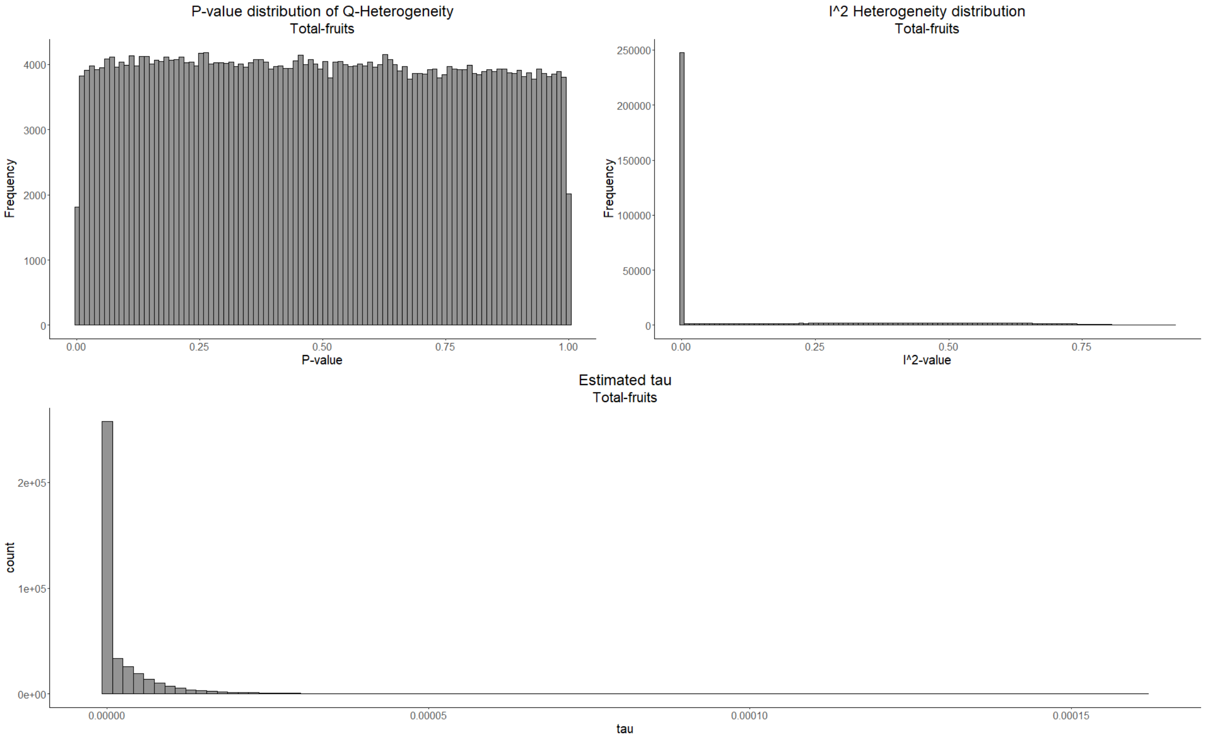

Fig 108

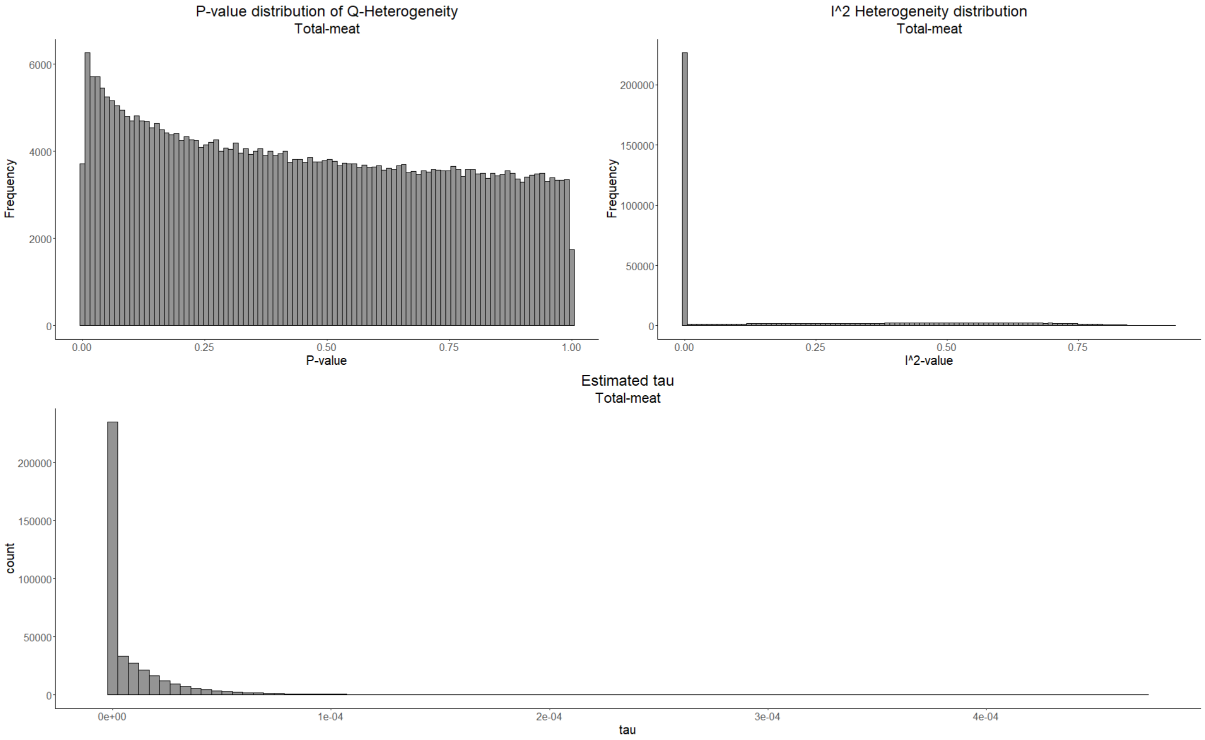

Fig 109

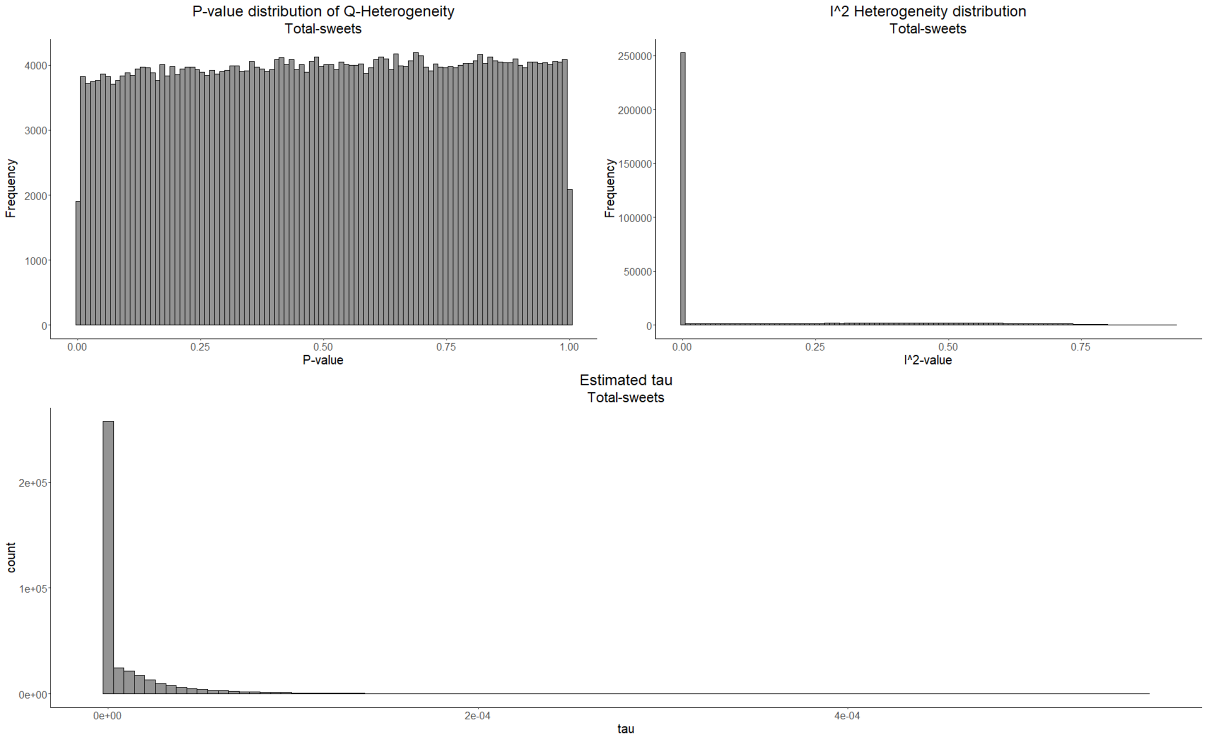

Fig 110

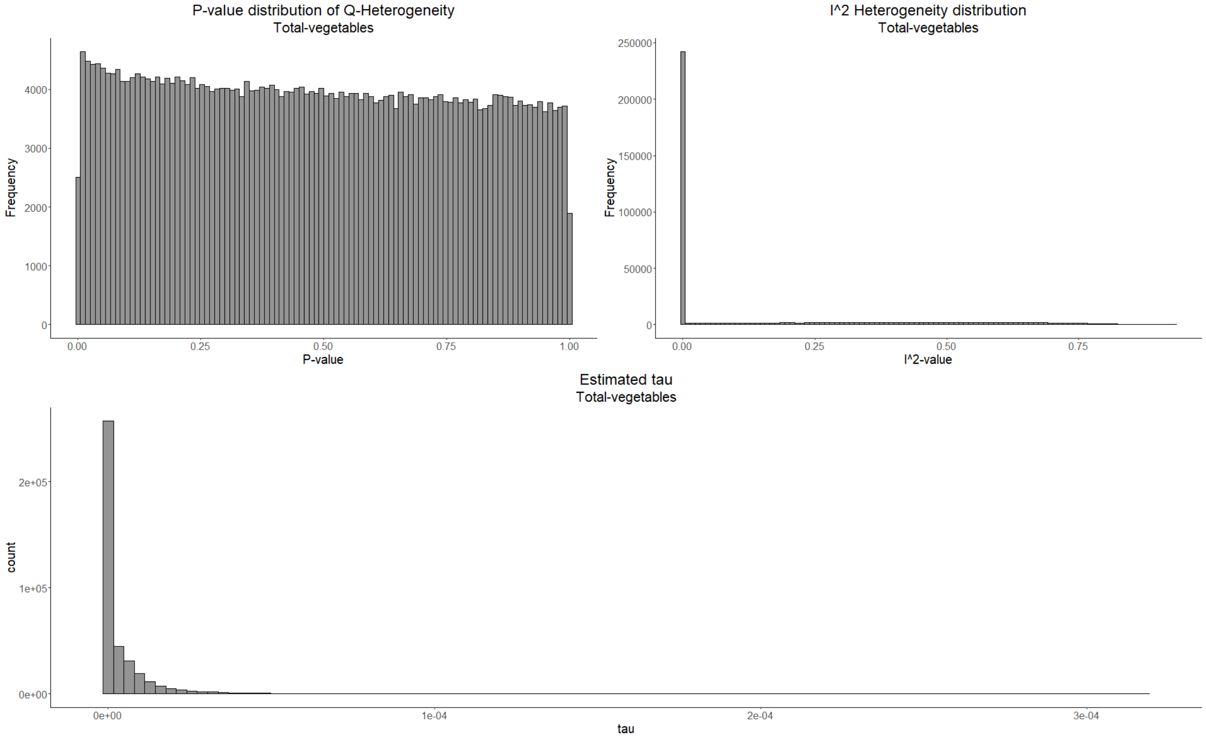

Fig 111

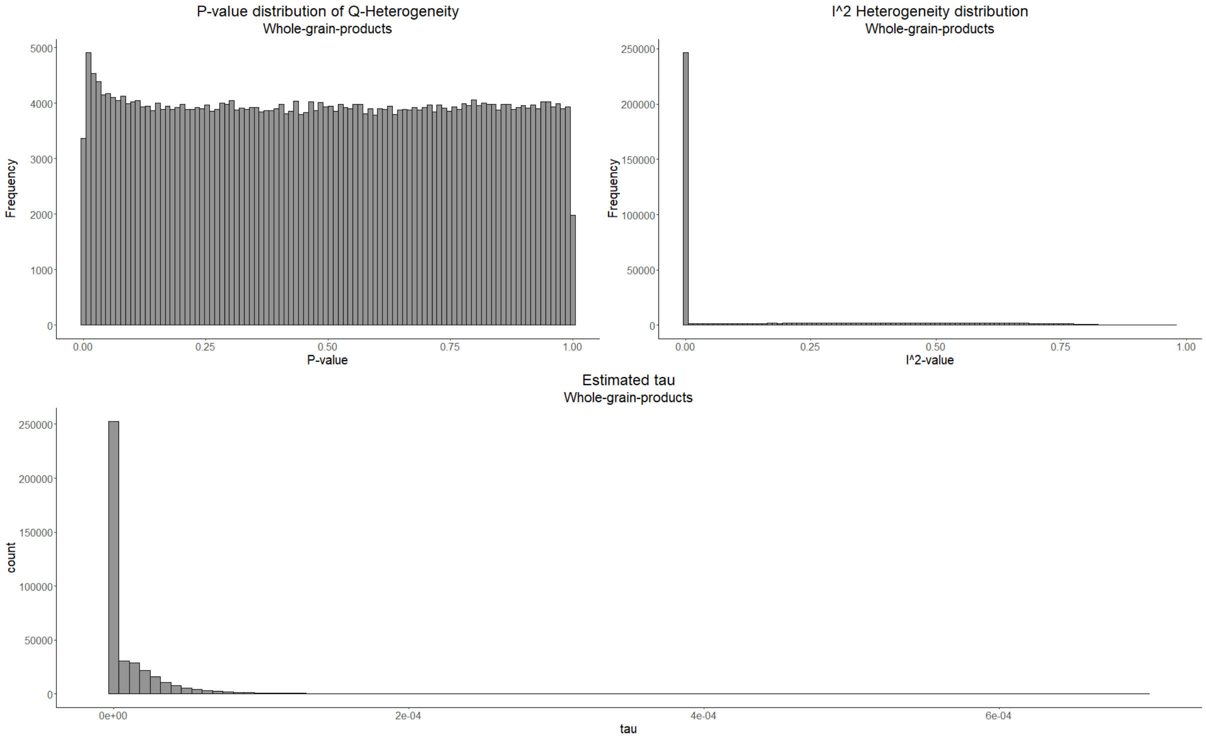

Fig 112

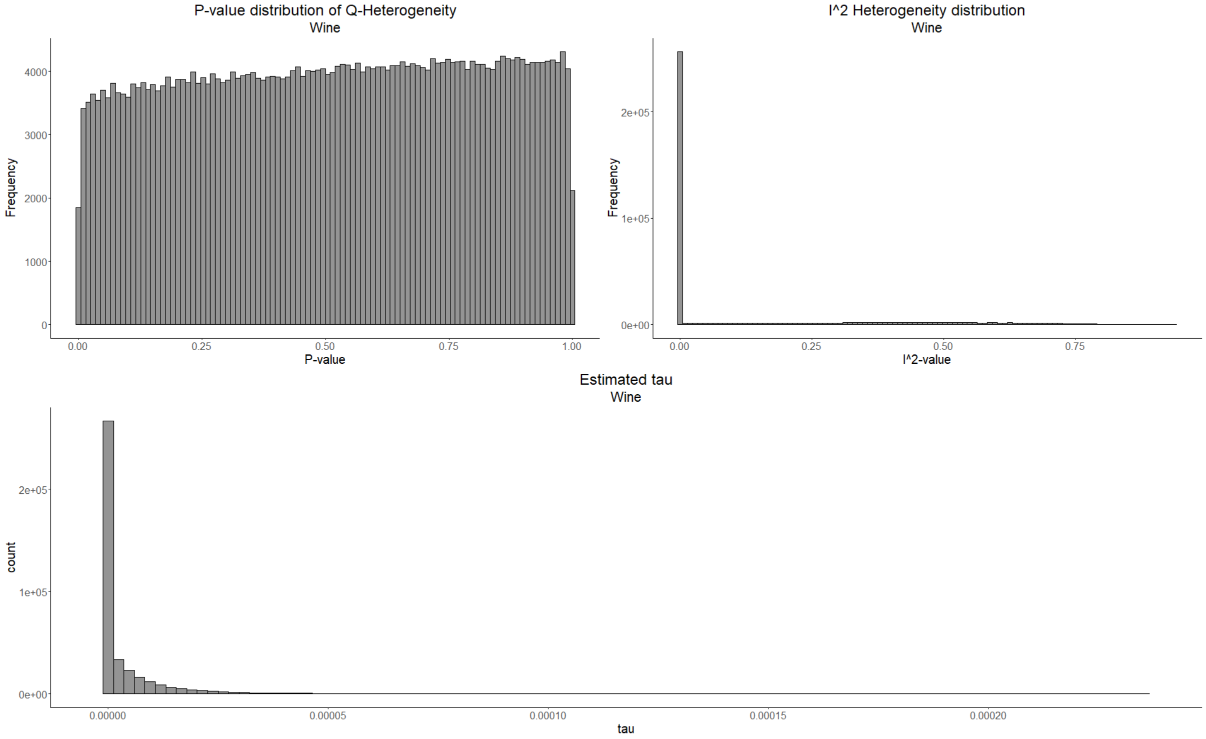

Fig 113

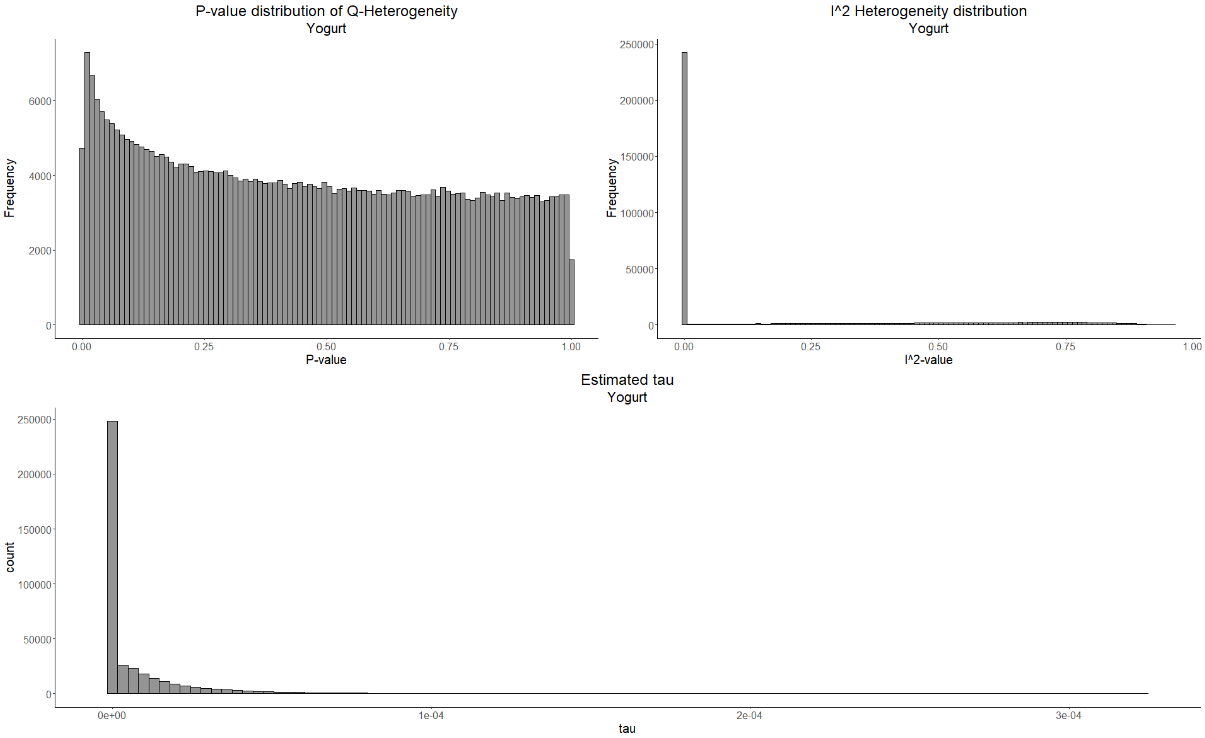
P-value distribution of Q-Heterogeneity
Yogurt
Frequency
0
2000
4000
6000
0.00
0.25
0.50
0.75
1.00
P-value
I^2 Heterogeneity distribution
Yogurt
Frequency
0
50000
100000
150000
200000
250000
0.00
0.25
0.50
0.75
1.00
I^2-value
Estimated tau
Yogurt
count
0
50000
100000
150000
200000
250000
0e+00
1e-04
2e-04
3e-04
tau
