# Supplementary material for: Pooled analysis of epigenome-wide association studies of food consumption in KORA, TwinsUK and LLS
Source: Eur J Nutr. 2022 Dec 26;62(3):1357–75. doi: 10.1007/s00394-022-03074-9 (PMC10030421; doi:10.1007/s00394-022-03074-9)
Supplement: Supplementary file 5 — Supplementary file5 (PDF 3114 KB) [file 394_2022_3074_MOESM5_ESM.pdf]

Article title: Pooled-analysis of epigenome-wide association studies of food consumption in KORA, TwinsUK and LLS

Journal name: European Journal of Nutrition

Author names: Fabian Hellbach, Lucy Sinke, Ricardo Costeira, Sebastian-Edgar Baumeister, Marian Beekman, Panayiotis Louca, Emily R Leeming, Olatz Mompeo, Sarah Berry, Rory Wilson, Nina Wawro, Dennis Freuer, Hans Hauner, Annette Peters, Juliane Winkelmann, Wolfgang Koenig, Christa Meisinger, Melanie Waldenberger, Bas Heijmans, Eline Slagboom, Jordana T Bell, Jakob Linseisen

Corresponding Author: Fabian Hellbach[1,2]

Email: fabian.hellbach@med.uni-augsburg.de

[1]Institute for Medical Information Processing, Biometry, and Epidemiology; Medical Faculty; Ludwig-Maximilian University Munich; Marchioninistr. 15, 81377 Munich, Germany

[2]Epidemiology; Faculty of Medicine; University of Augsburg; University Hospital Augsburg; Stenglinstraße 2, 86156 Augsburg; Germany

Caption: Volcano plots for every food group analyzed. Effect size on the x-axis is %-methylation change per gram residual/day. Turquoise dots were significant after false-discovery rate correction.

Fig 114

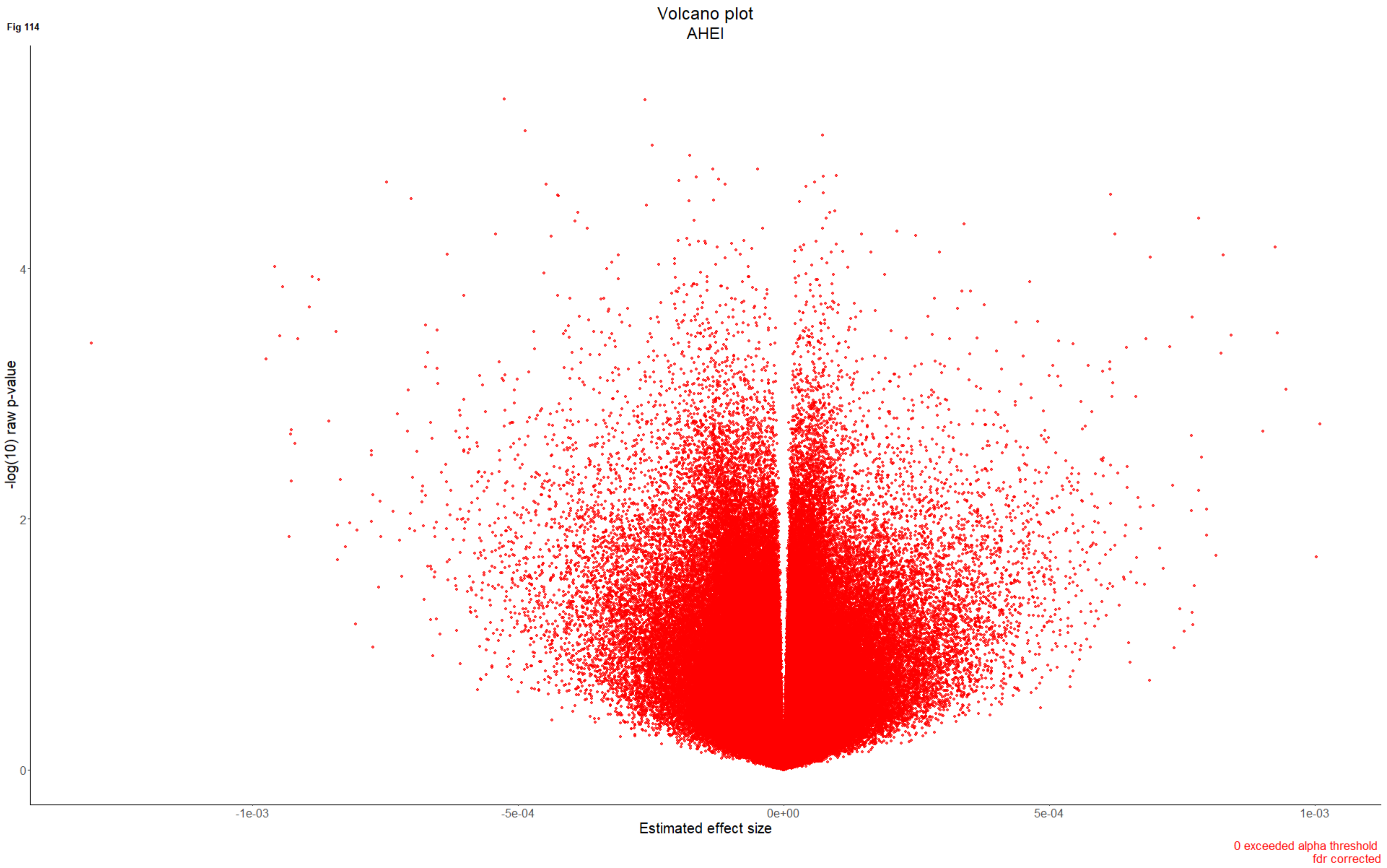
Volcano plot
AHEI
-log(10) raw p-value
0
2
4
-1e-03
-5e-04
0e+00
5e-04
1e-03
Estimated effect size
0 exceeded alpha threshold
fdr corrected

Fig 115

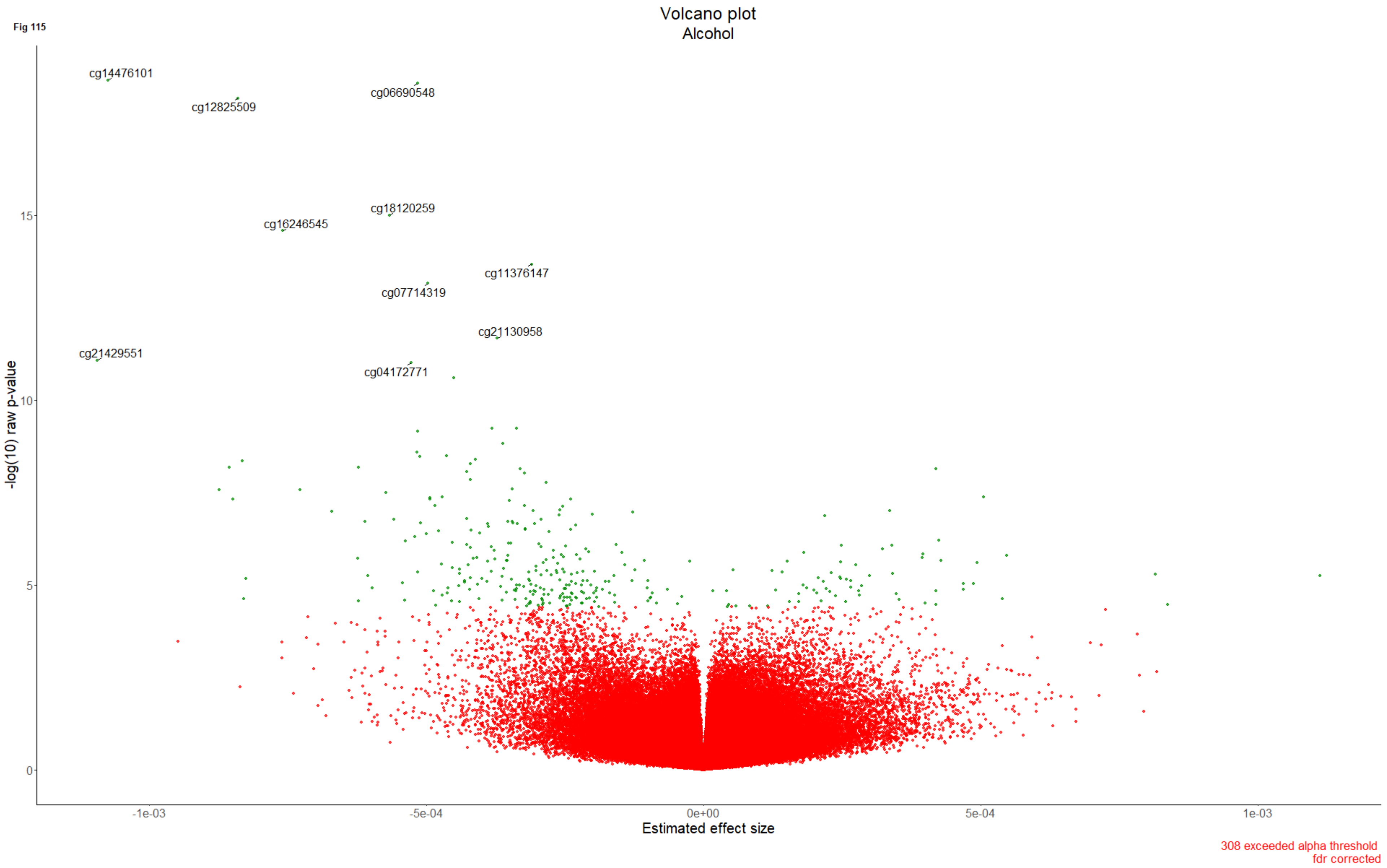
Volcano plot
Alcohol
cg14476101
cg06690548
cg12825509
cg18120259
cg16246545
cg11376147
cg07714319
cg21130958
cg21429551
cg04172771
-log(10) raw p-value
15
10
5
0
-1e-03
-5e-04
0e+00
5e-04
1e-03
Estimated effect size
308 exceeded alpha threshold
fdr corrected

Fig 116

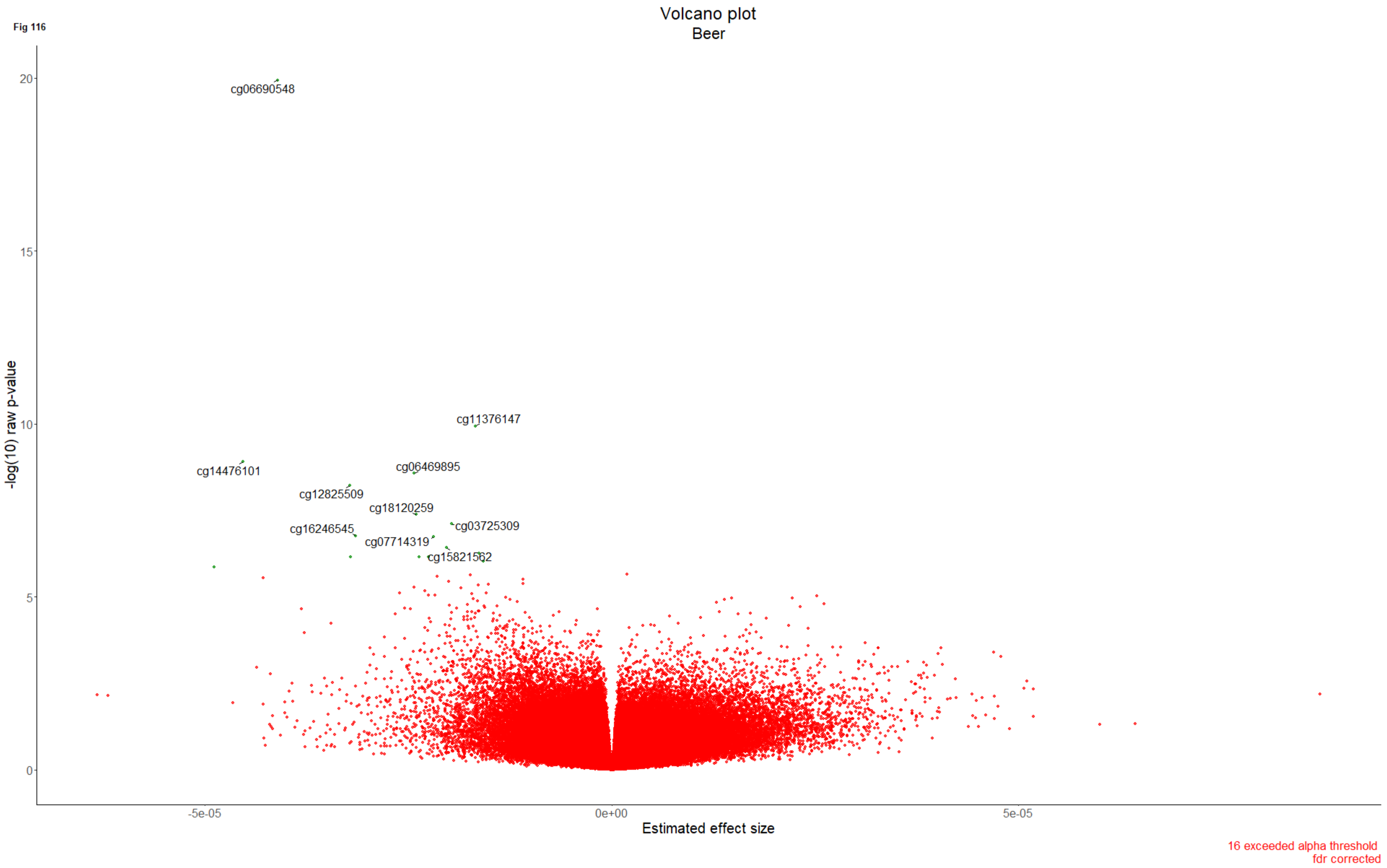

Volcano plot
Beer
-log(10) raw p-value
Estimated effect size
cg06690548
cg11376147
cg14476101
cg06469895
cg12825509
cg18120259
cg16246545
cg03725309
cg07714319
cg15821562
20
15
10
5
0
-5e-05
0e+00
5e-05
16 exceeded alpha threshold
fdr corrected

Fig 117

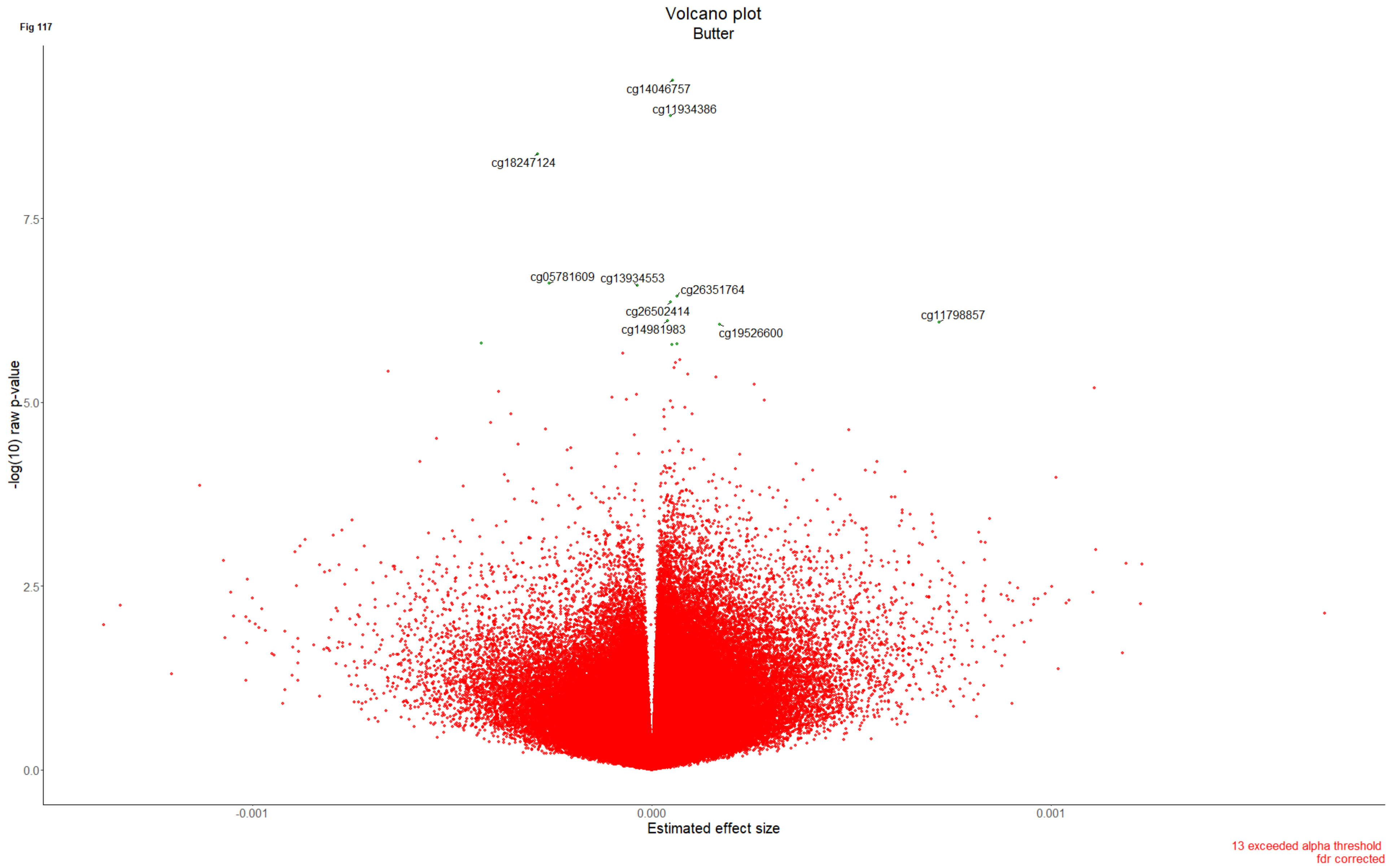
Volcano plot
Butter
cg14046757
cg11934386
cg18247124
cg05781609
cg13934553
cg26351764
cg26502414
cg14981983
cg19526600
cg11798857
7.5
5.0
2.5
0.0
-log(10) raw p-value
-0.001
0.000
0.001
Estimated effect size
13 exceeded alpha threshold
fdr corrected

Fig 118

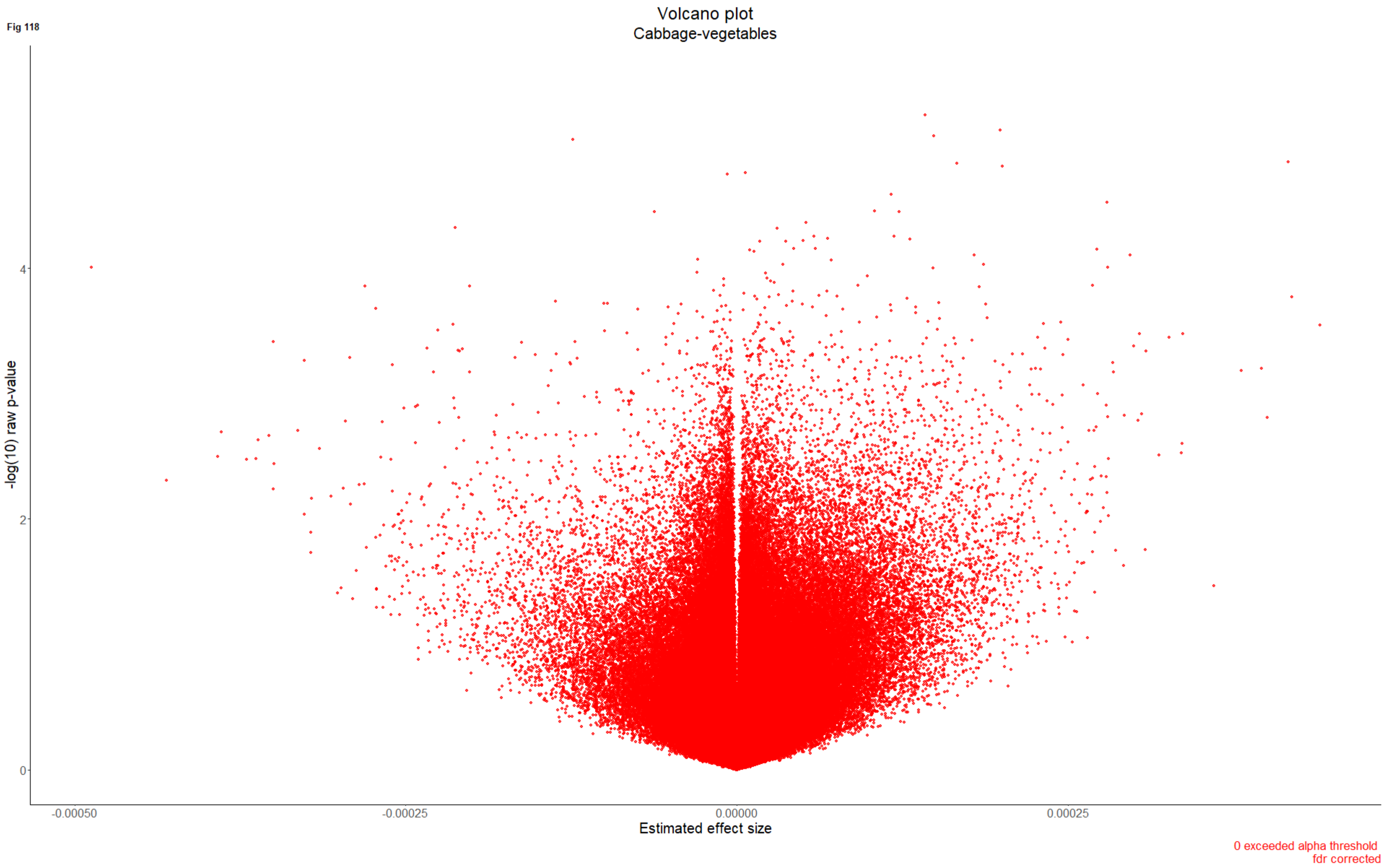
Volcano plot
Cabbage-vegetables
-log(10) raw p-value
4
2
0
-0.00050
-0.00025
0.00000
0.00025
Estimated effect size
0 exceeded alpha threshold
fdr corrected

Fig 119

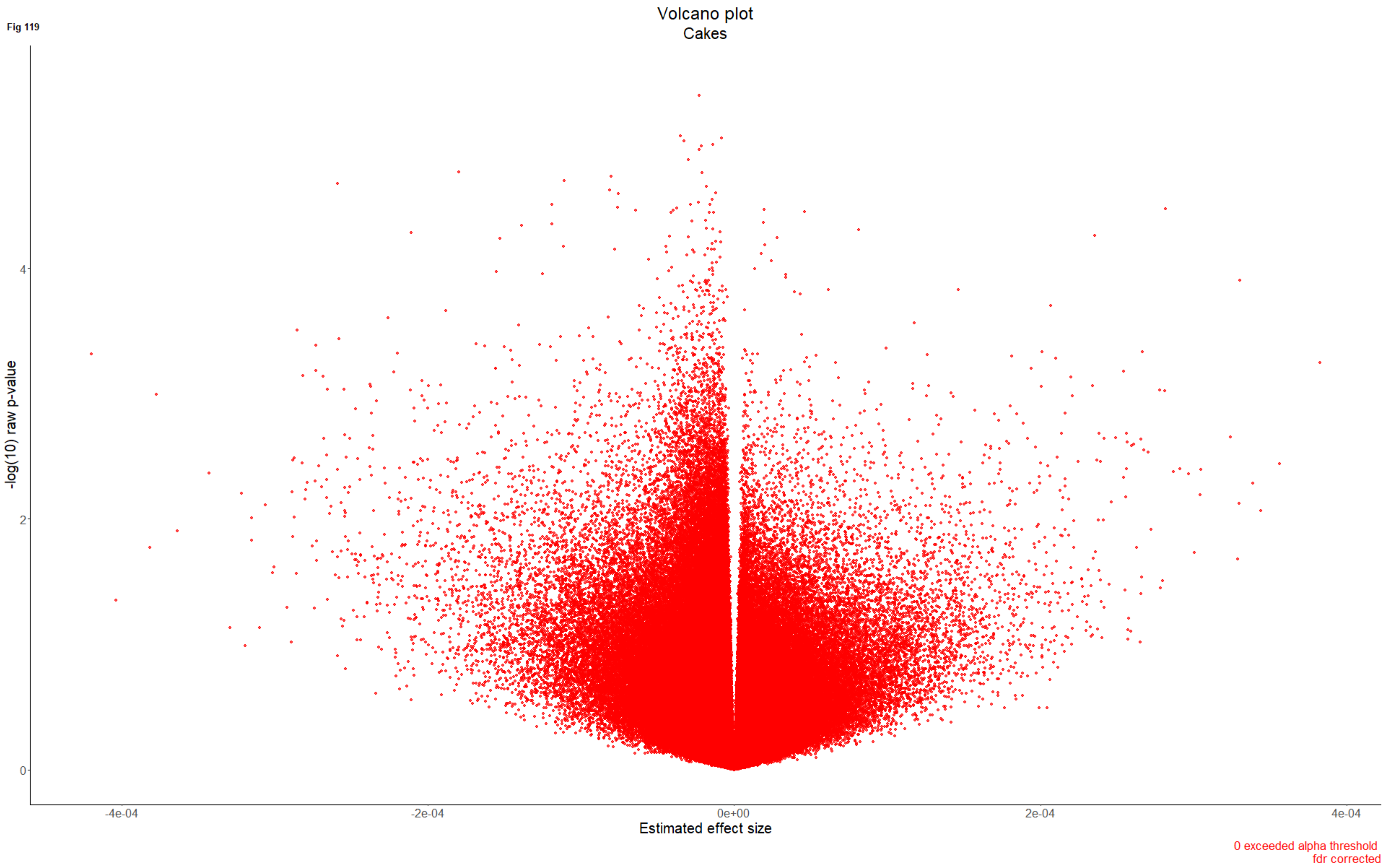
Volcano plot
Cakes
-log(10) raw p-value
0
2
4
-4e-04
-2e-04
0e+00
2e-04
4e-04
Estimated effect size
0 exceeded alpha threshold
fdr corrected

Fig 120

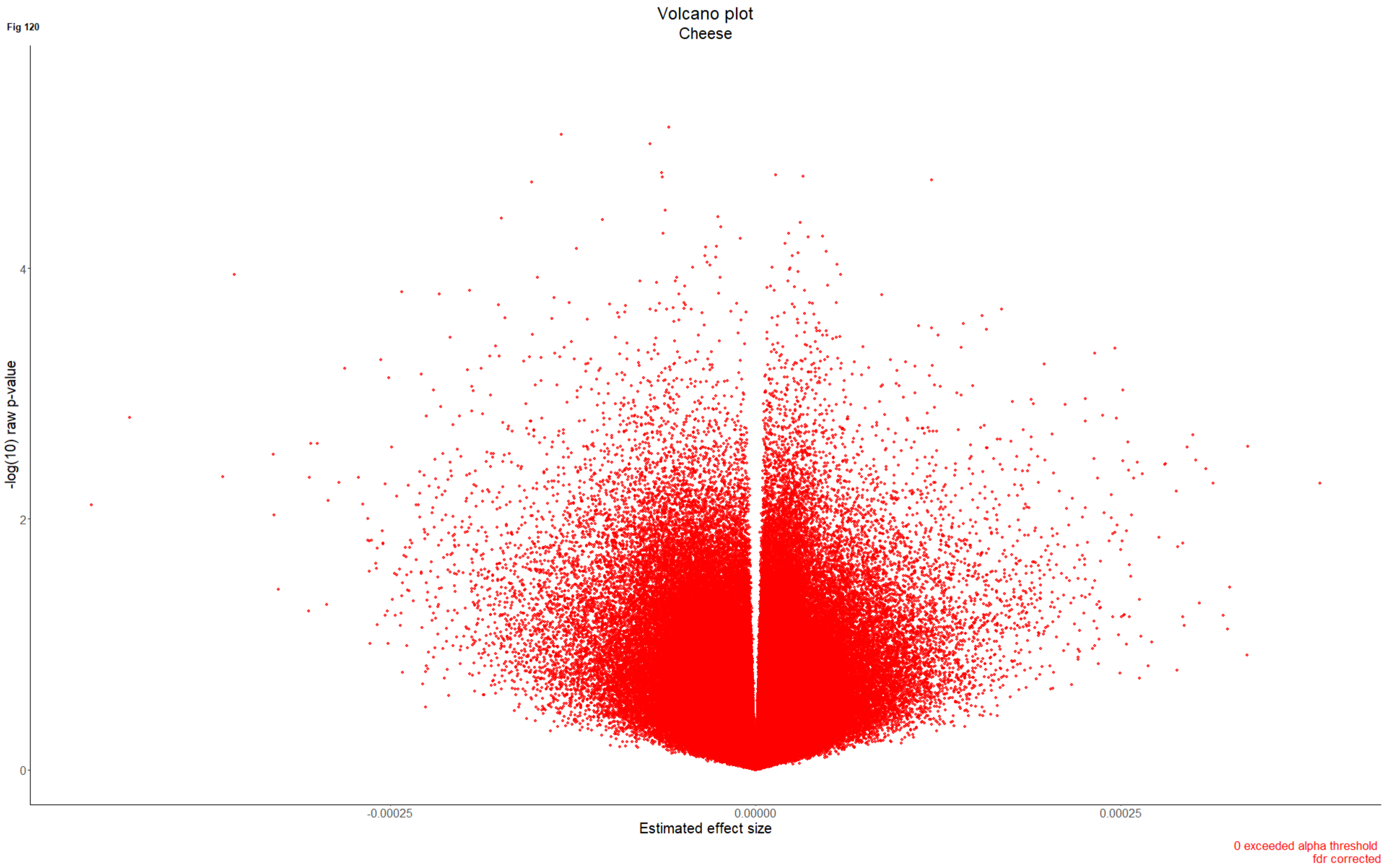

Volcano plot
Cheese
-log(10) raw p-value
4
2
0
-0.00025
0.00000
0.00025
Estimated effect size
0 exceeded alpha threshold
fdr corrected

Fig 121

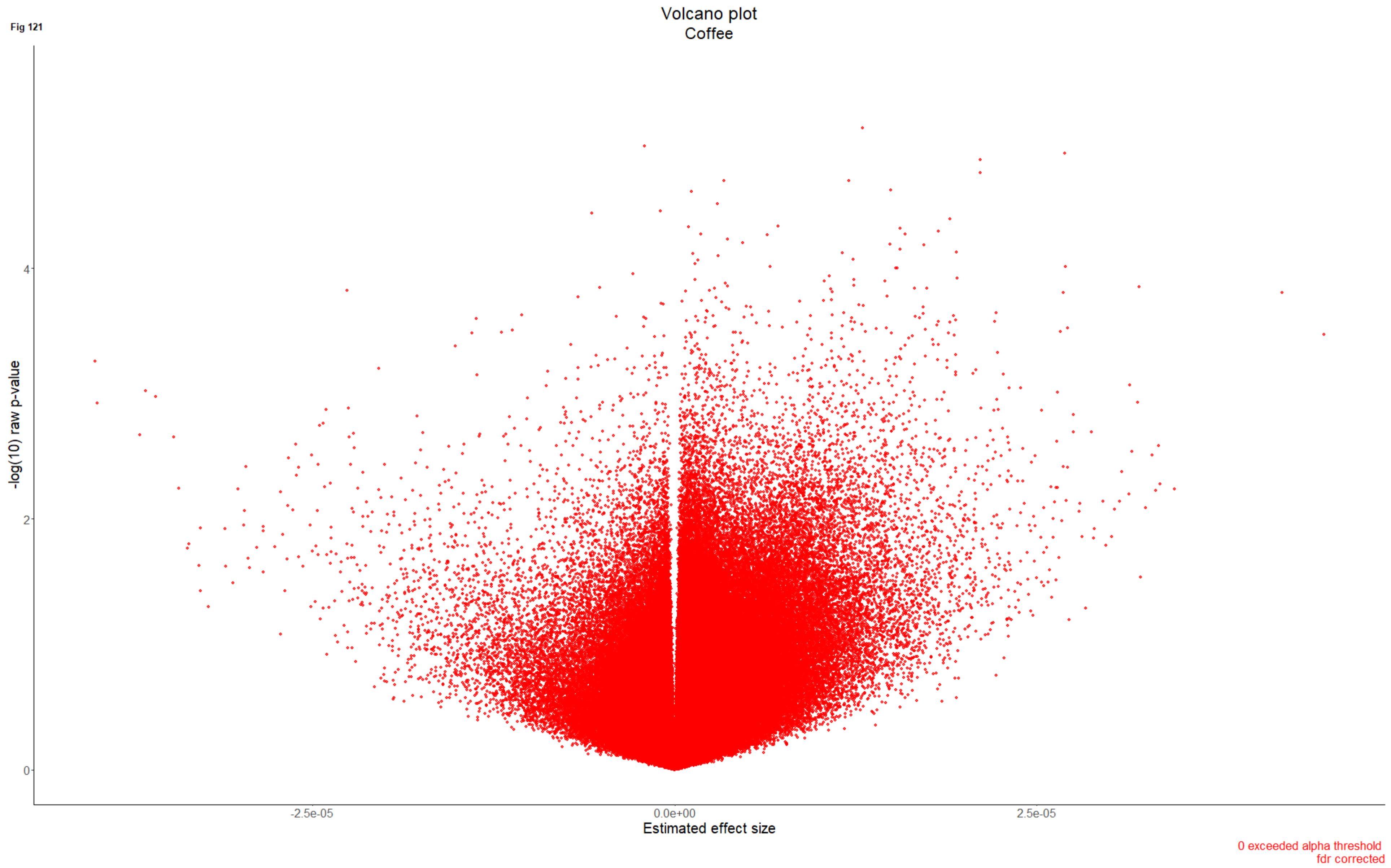
Volcano plot
Coffee
-log(10) raw p-value
4
2
0
-2.5e-05
0.0e+00
2.5e-05
Estimated effect size
0 exceeded alpha threshold
fdr corrected

Fig 122

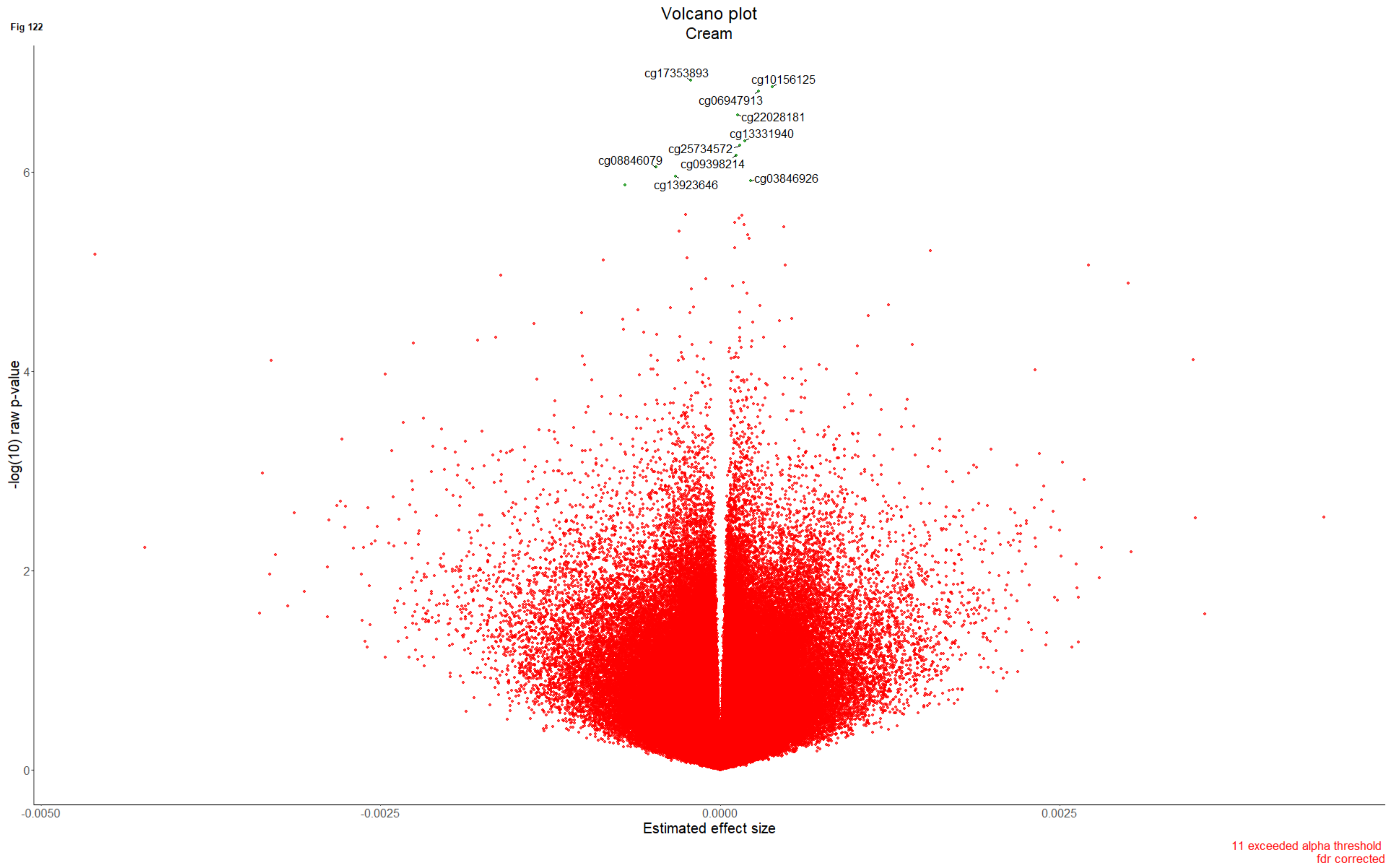
Volcano plot
Cream
cg17353893
cg10156125
cg06947913
cg22028181
cg13331940
cg25734572
cg08846079
cg09398214
cg03846926
cg13923646
-log(10) raw p-value
6
4
2
0
-0.0050
-0.0025
0.0000
0.0025
Estimated effect size
11 exceeded alpha threshold
fdr corrected

Fig 123

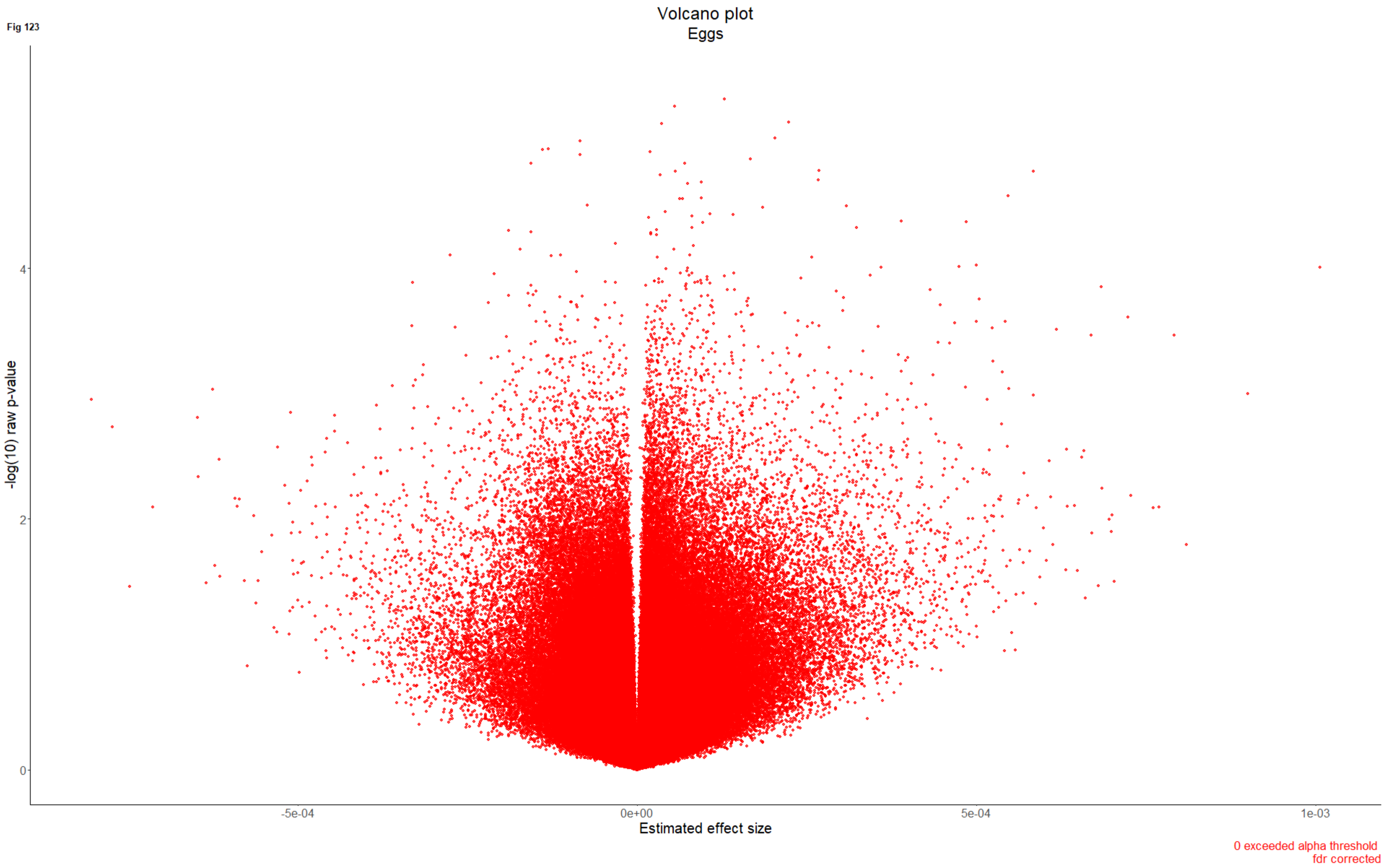
Volcano plot
Eggs
-log(10) raw p-value
4
2
0
-5e-04
0e+00
5e-04
1e-03
Estimated effect size
0 exceeded alpha threshold
fdr corrected

Fig 124

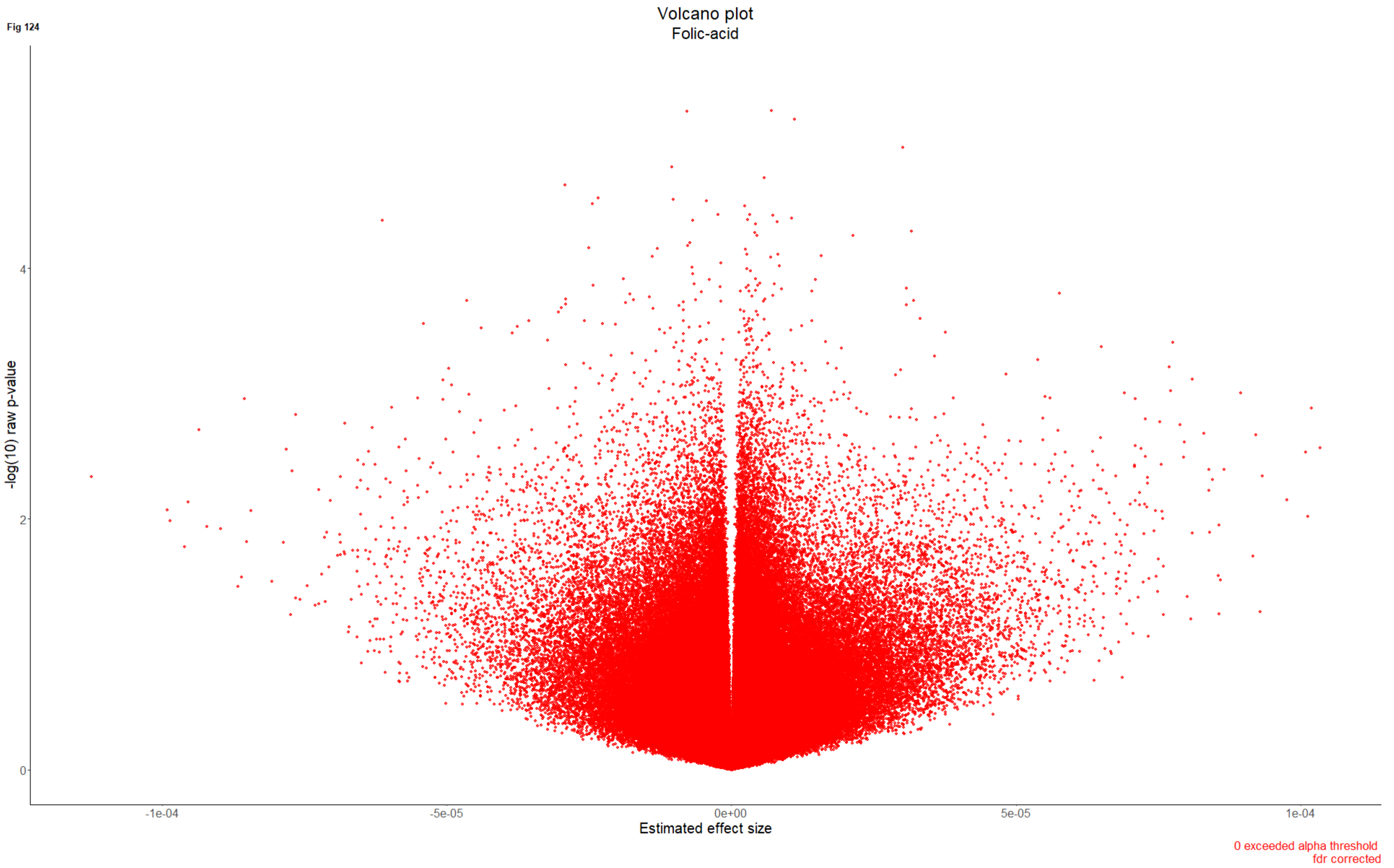
Volcano plot
Folic-acid
-log(10) raw p-value
0
2
4
-1e-04
-5e-05
0e+00
5e-05
1e-04
Estimated effect size
0 exceeded alpha threshold
fdr corrected

Fig 125

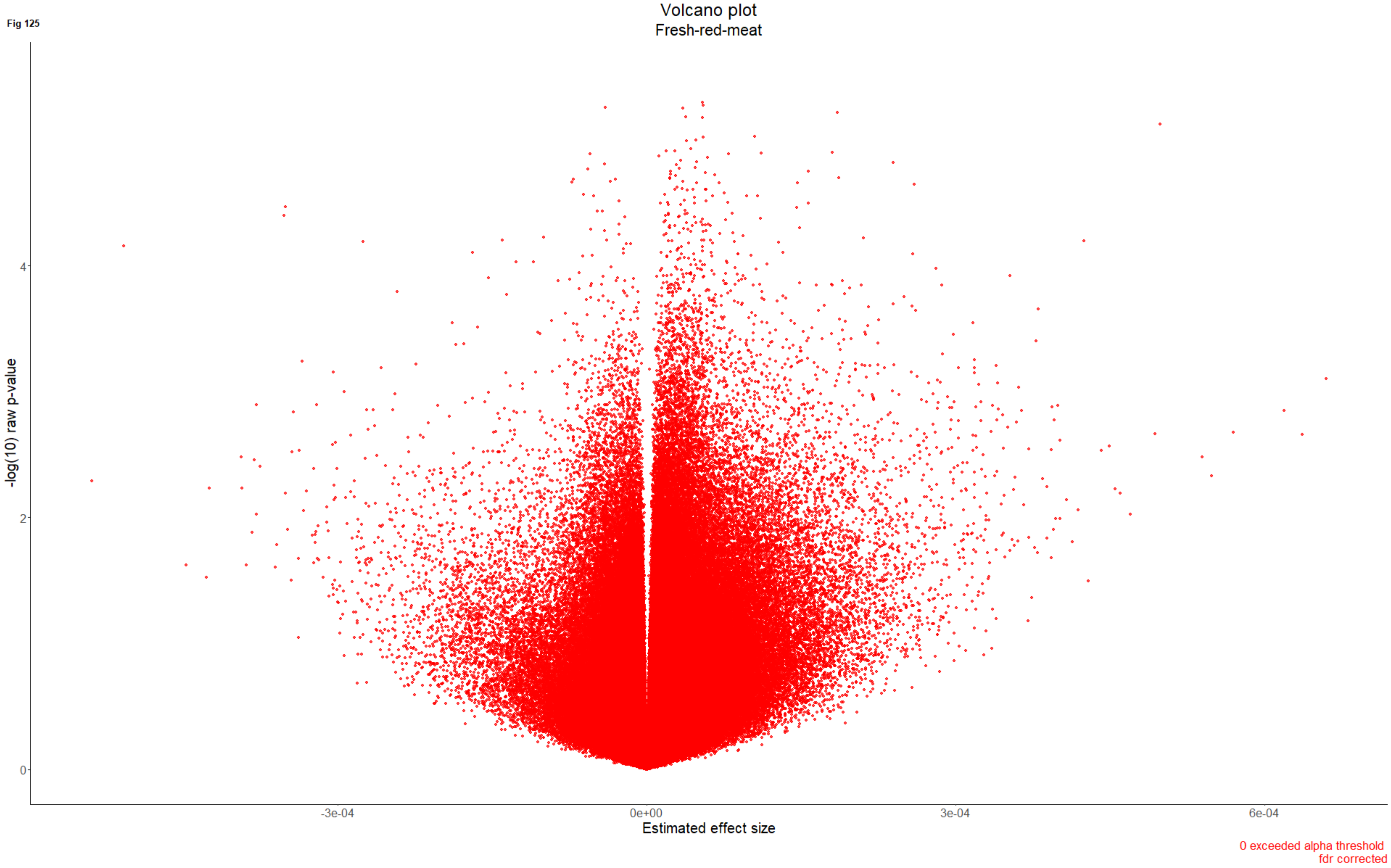
Volcano plot
Fresh-red-meat
-log(10) raw p-value
4
2
0
-3e-04
0e+00
3e-04
6e-04
Estimated effect size
0 exceeded alpha threshold
fdr corrected

Fig 126

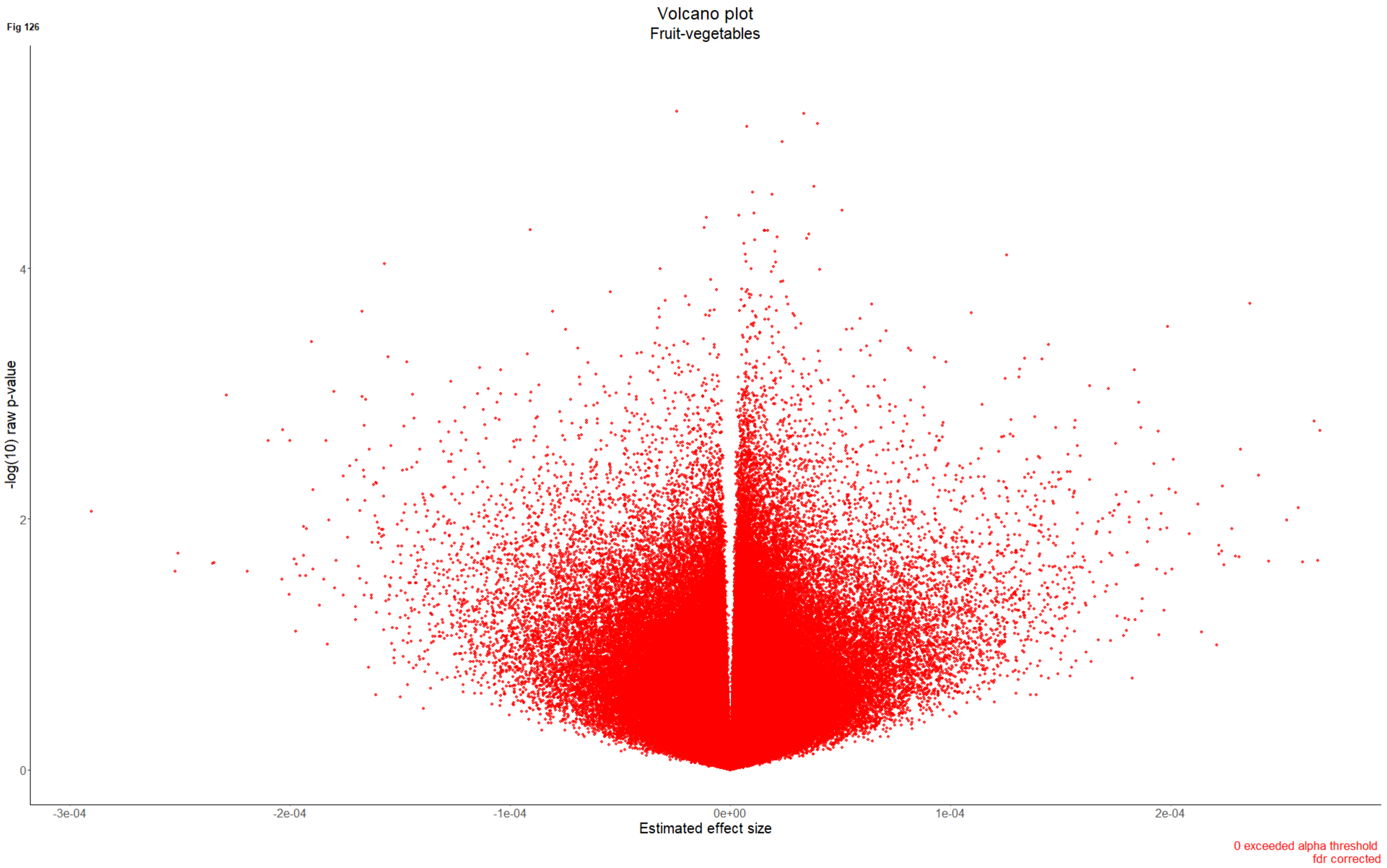

Volcano plot
Fruit-vegetables
-log(10) raw p-value
0
2
4
-3e-04
-2e-04
-1e-04
0e+00
1e-04
2e-04
Estimated effect size
0 exceeded alpha threshold
fdr corrected

Fig 127

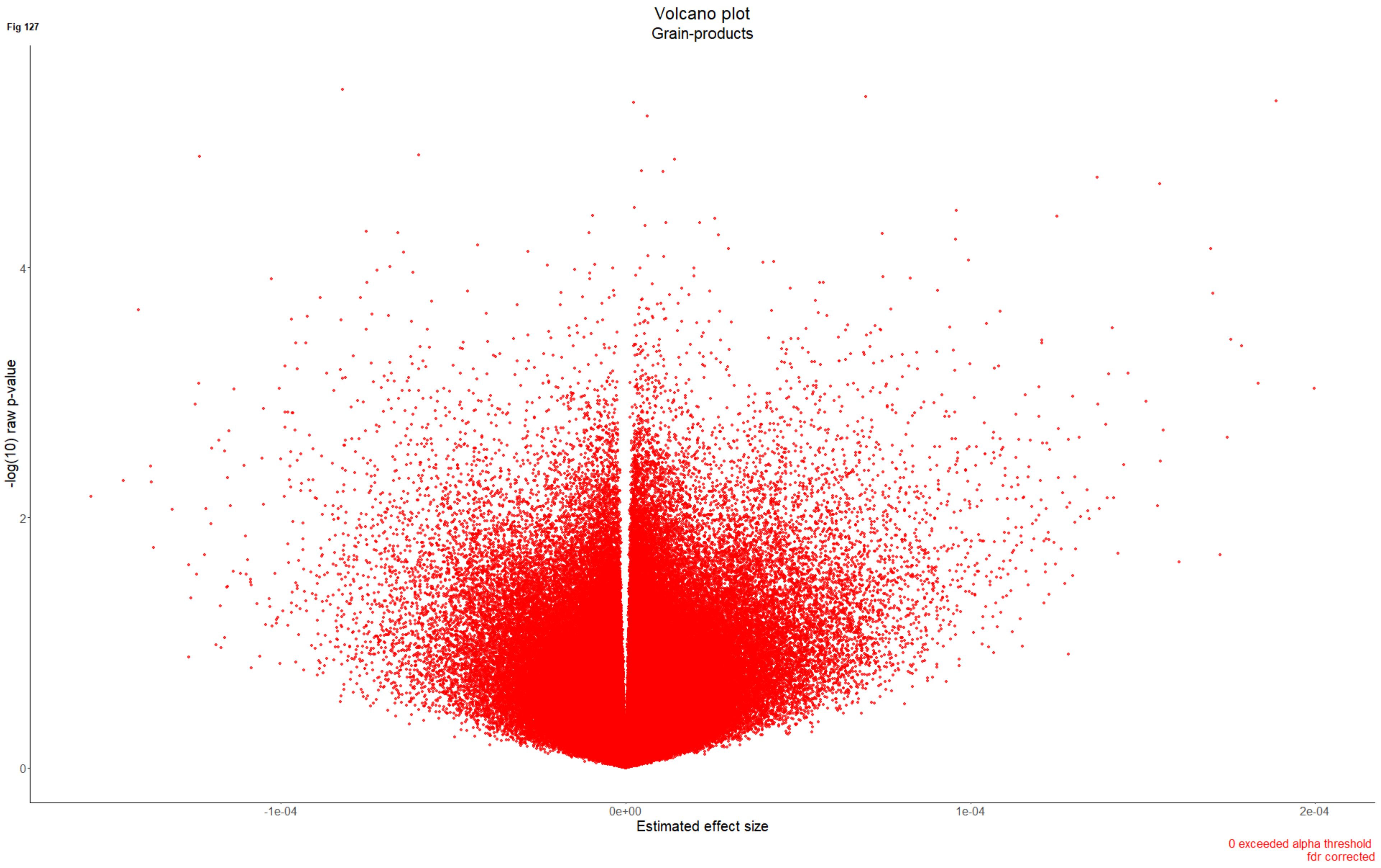
Volcano plot
Grain-products
-log(10) raw p-value
4
2
0
-1e-04
0e+00
1e-04
2e-04
Estimated effect size
0 exceeded alpha threshold
fdr corrected

Fig 128

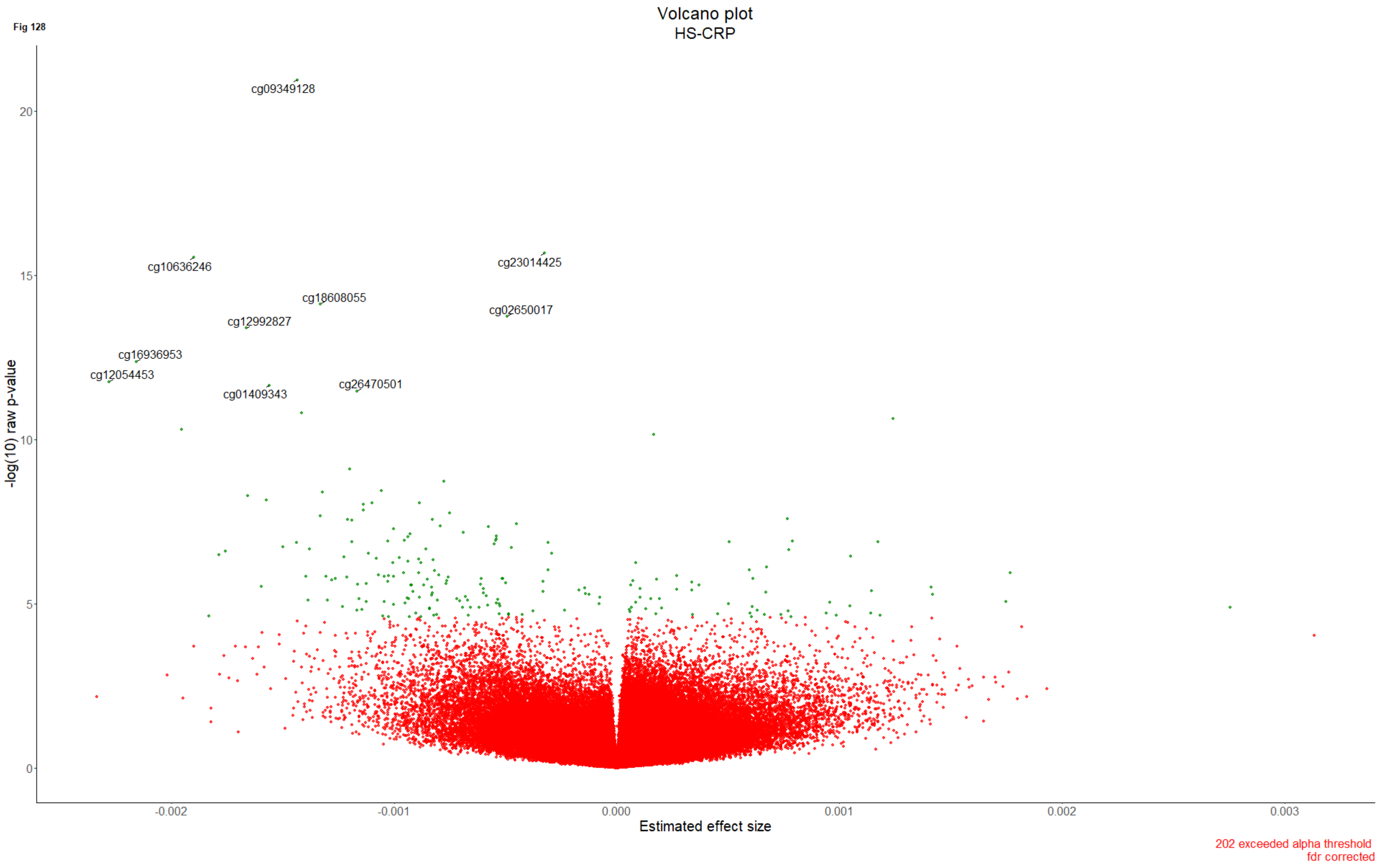
Volcano plot
HS-CRP
cg09349128
cg10636246
cg23014425
cg18608055
cg02650017
cg12992827
cg16936953
cg12054453
cg01409343
cg26470501
20
15
10
5
0
-log(10) raw p-value
-0.002
-0.001
0.000
0.001
0.002
0.003
Estimated effect size
202 exceeded alpha threshold
fdr corrected

Fig 129

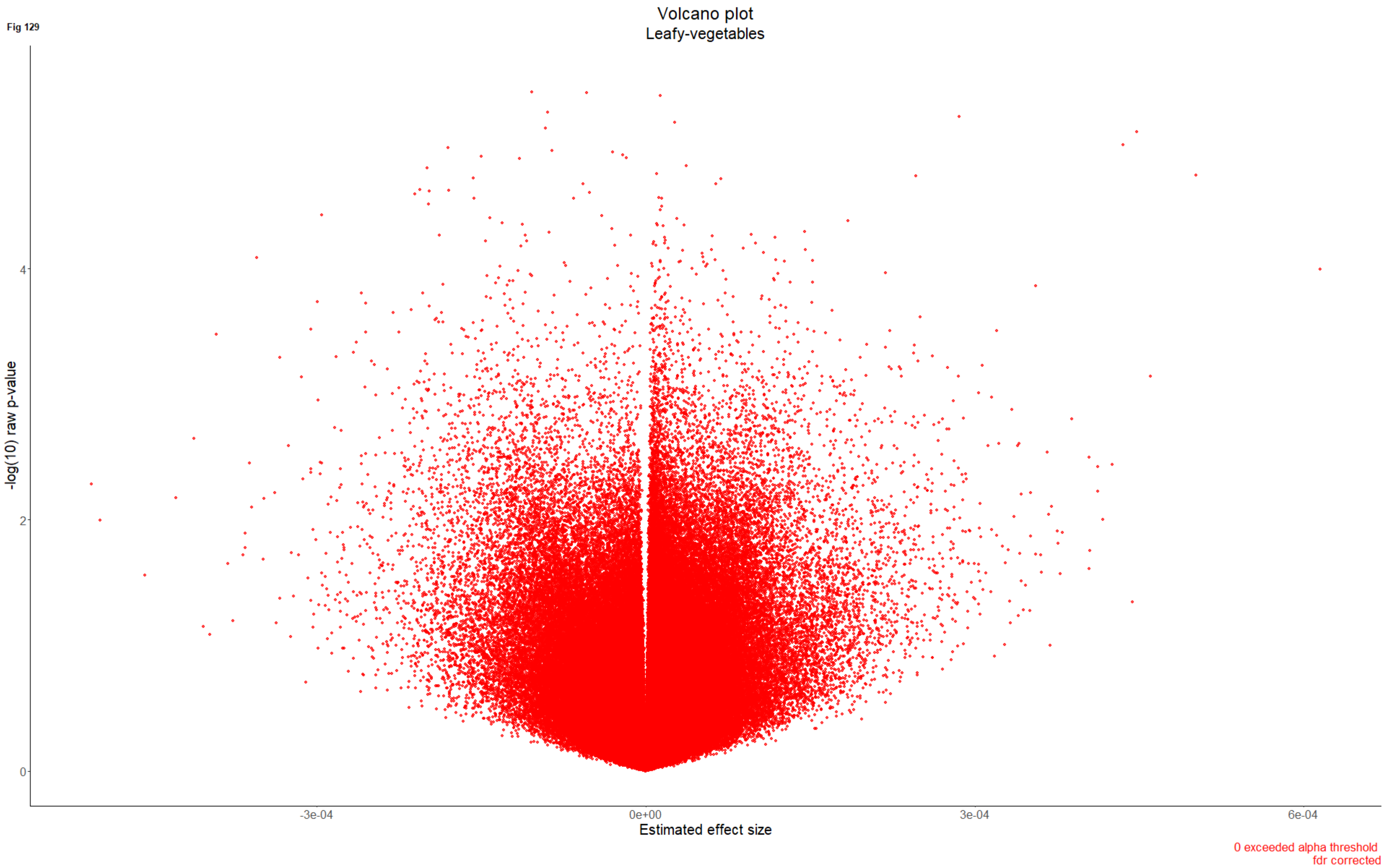

Volcano plot
Leafy-vegetables
-log(10) raw p-value
4
2
0
-3e-04
0e+00
3e-04
6e-04
Estimated effect size
0 exceeded alpha threshold
fdr corrected

Fig 130

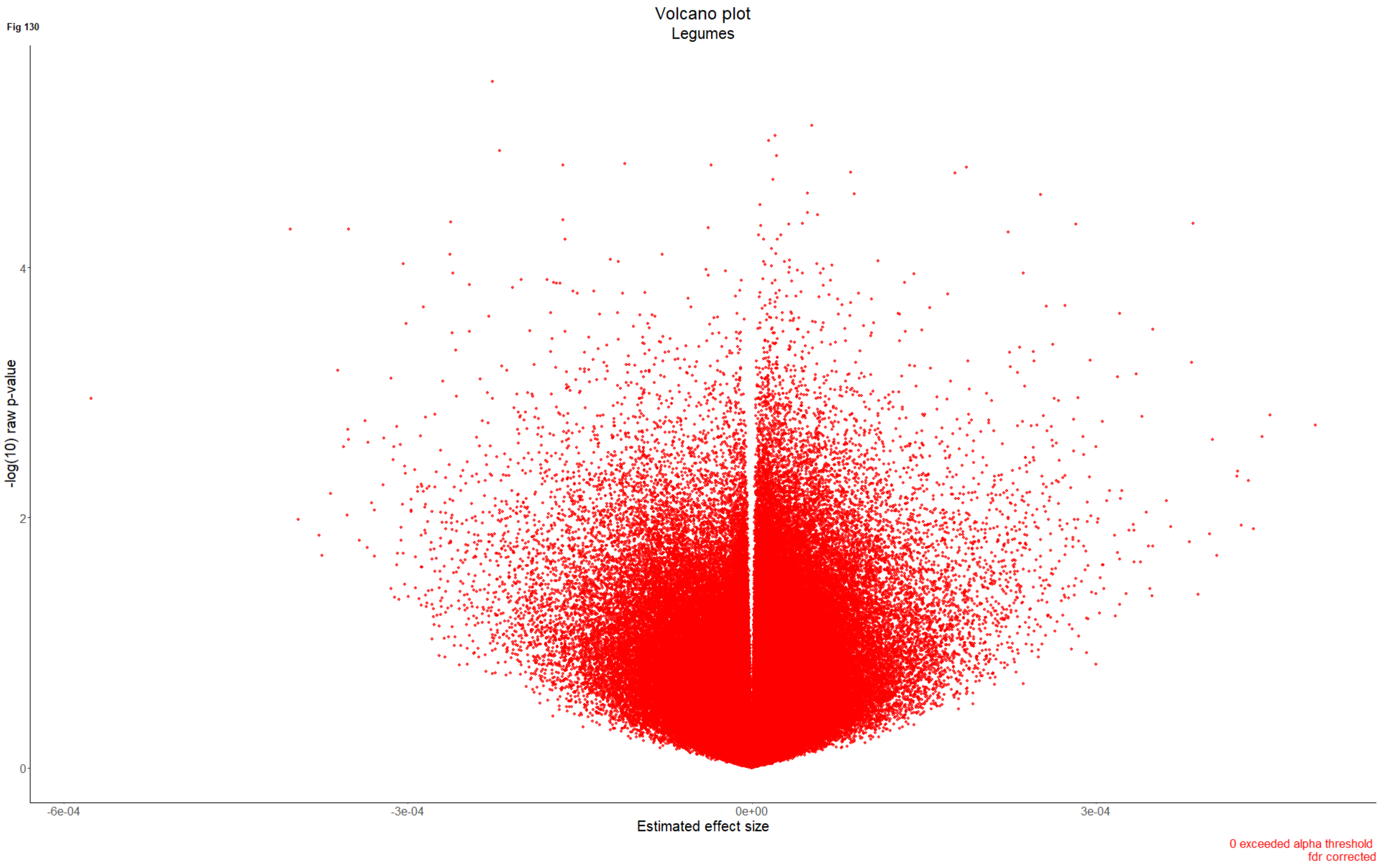
Volcano plot
Legumes
-log(10) raw p-value
4
2
0
-6e-04
-3e-04
0e+00
3e-04
Estimated effect size
0 exceeded alpha threshold
fdr corrected

Fig 131

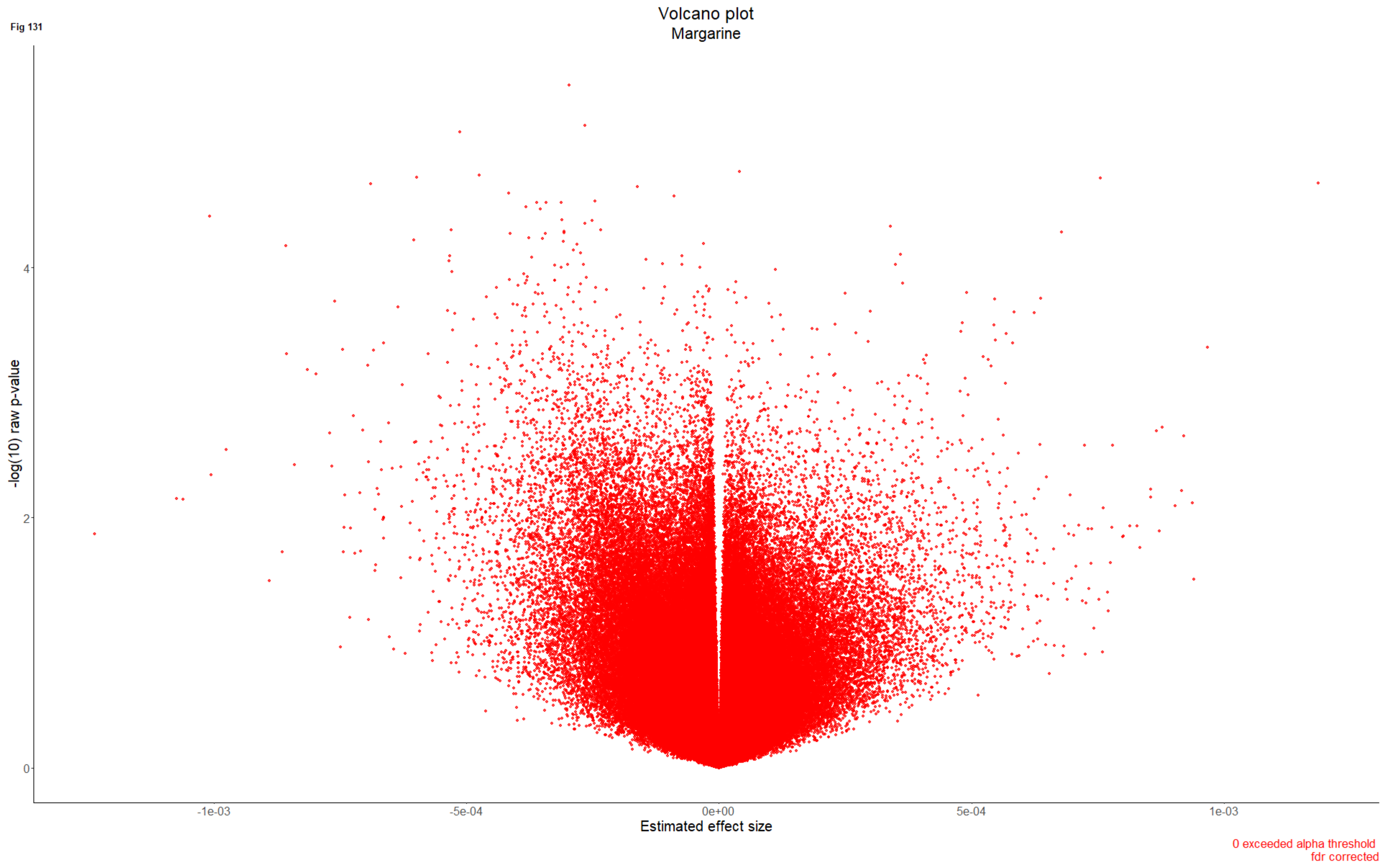
Volcano plot
Margarine
-log(10) raw p-value
0
2
4
-1e-03
-5e-04
0e+00
5e-04
1e-03
Estimated effect size
0 exceeded alpha threshold
fdr corrected

Fig 132

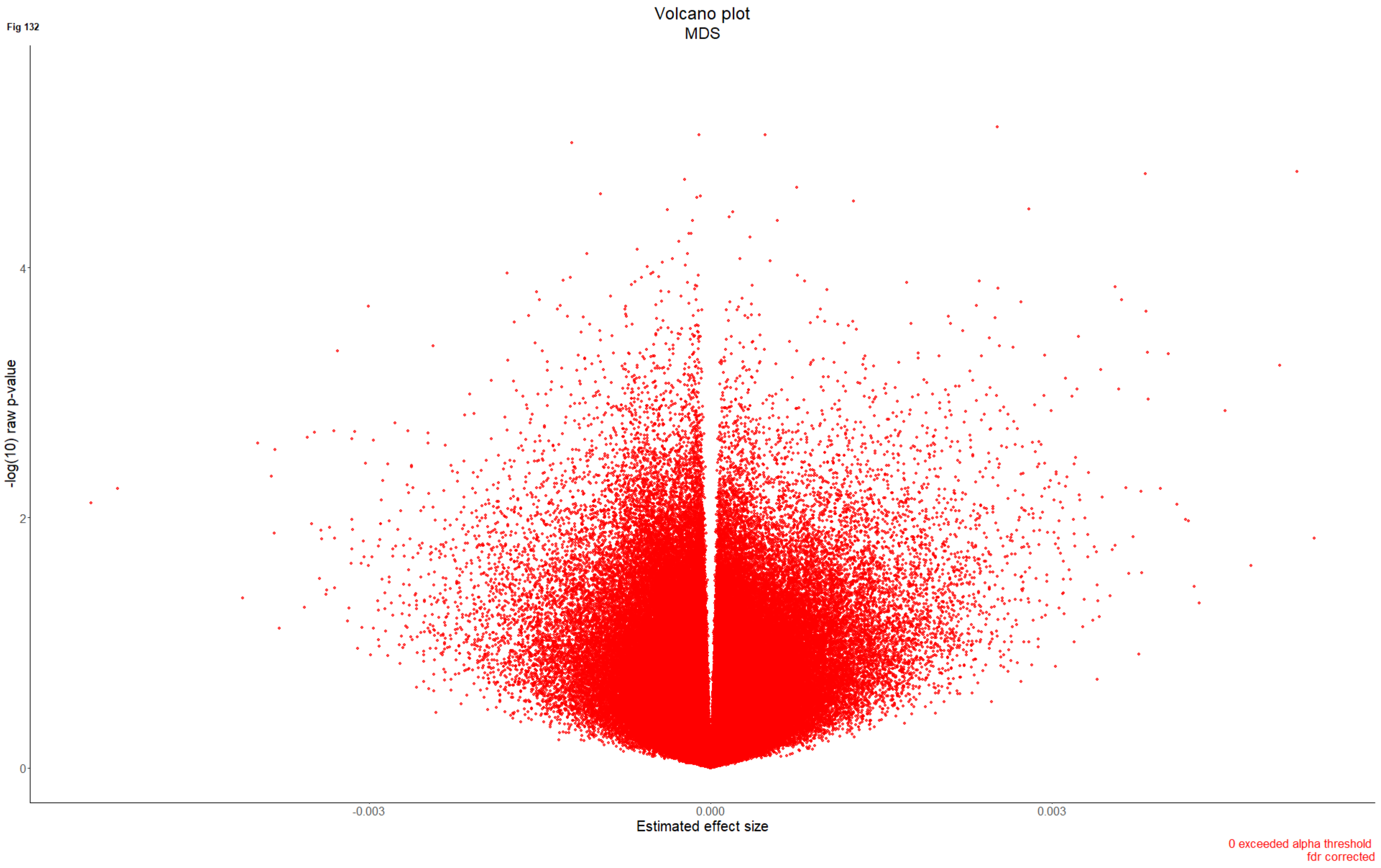
Volcano plot
MDS
-log(10) raw p-value
4
2
0
-0.003
0.000
0.003
Estimated effect size
0 exceeded alpha threshold
fdr corrected

Fig 133

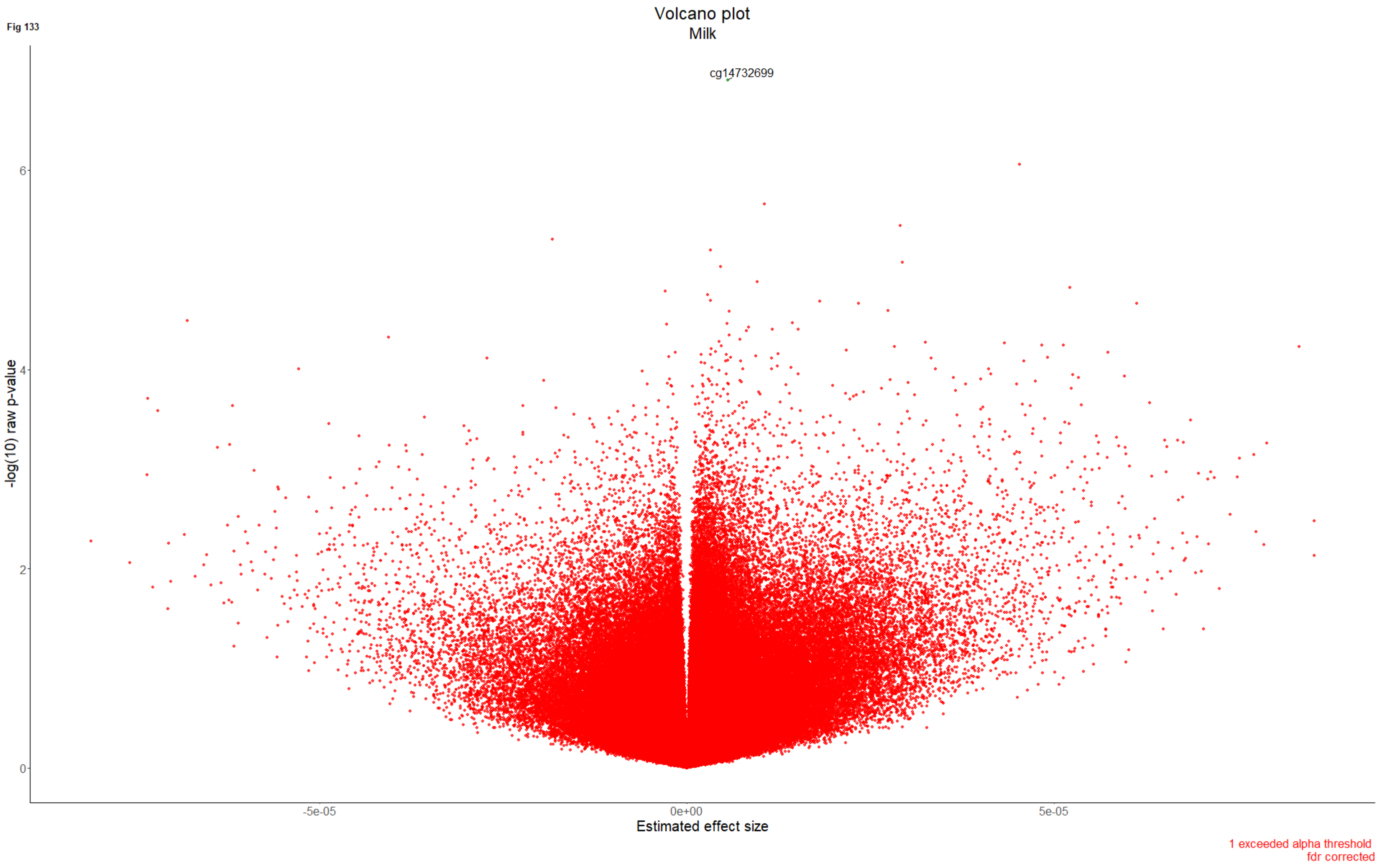
Volcano plot
Milk
cg14732699
-log(10) raw p-value
0
2
4
6
-5e-05
0e+00
5e-05
Estimated effect size
1 exceeded alpha threshold
fdr corrected

Fig 134

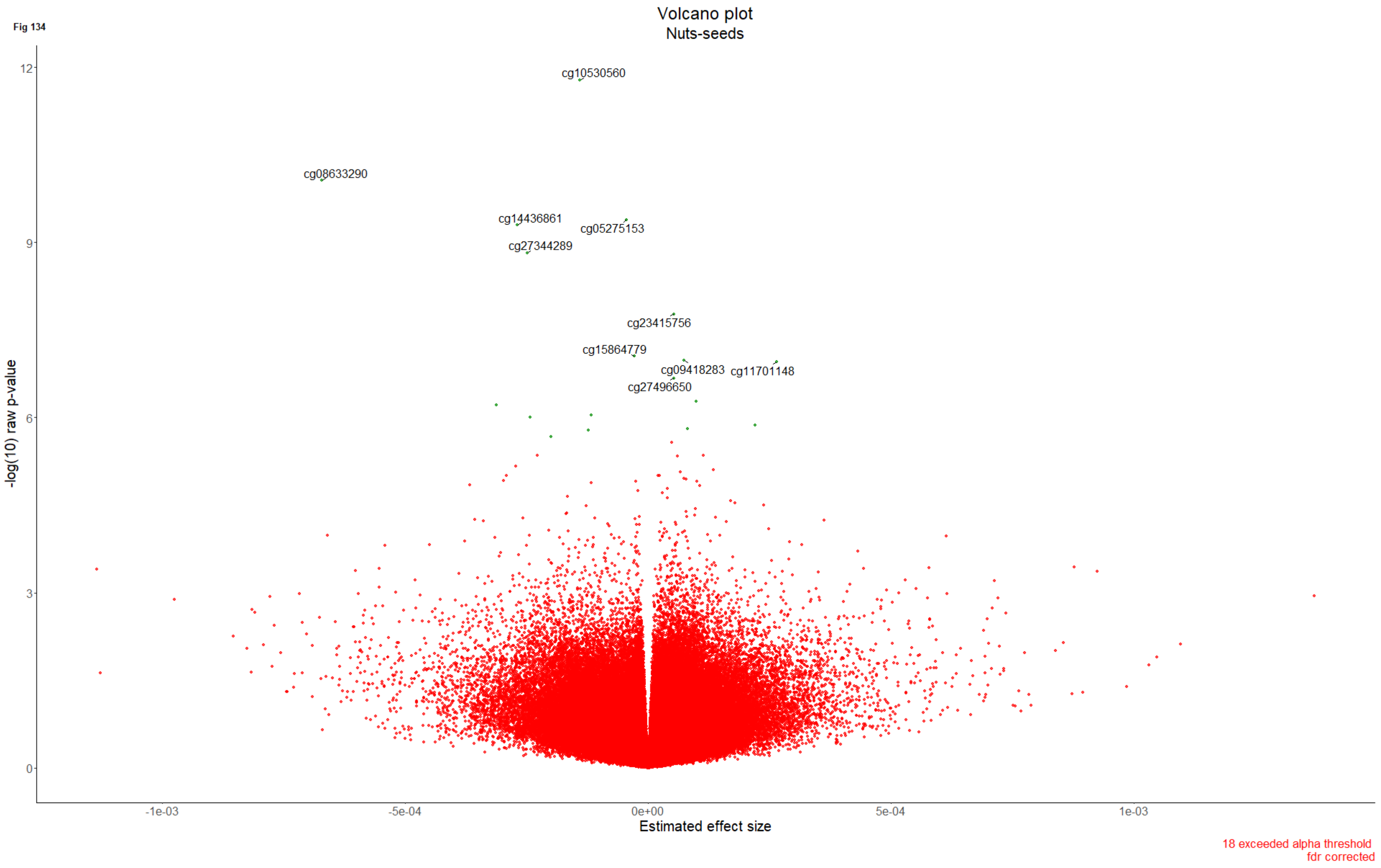
Volcano plot
Nuts-seeds
cg10530560
cg08633290
cg14436861
cg05275153
cg27344289
cg23415756
cg15864779
cg09418283
cg11701148
cg27496650
-log(10) raw p-value
12
9
6
3
0
-1e-03
-5e-04
0e+00
5e-04
1e-03
Estimated effect size
18 exceeded alpha threshold
fdr corrected

Fig 135

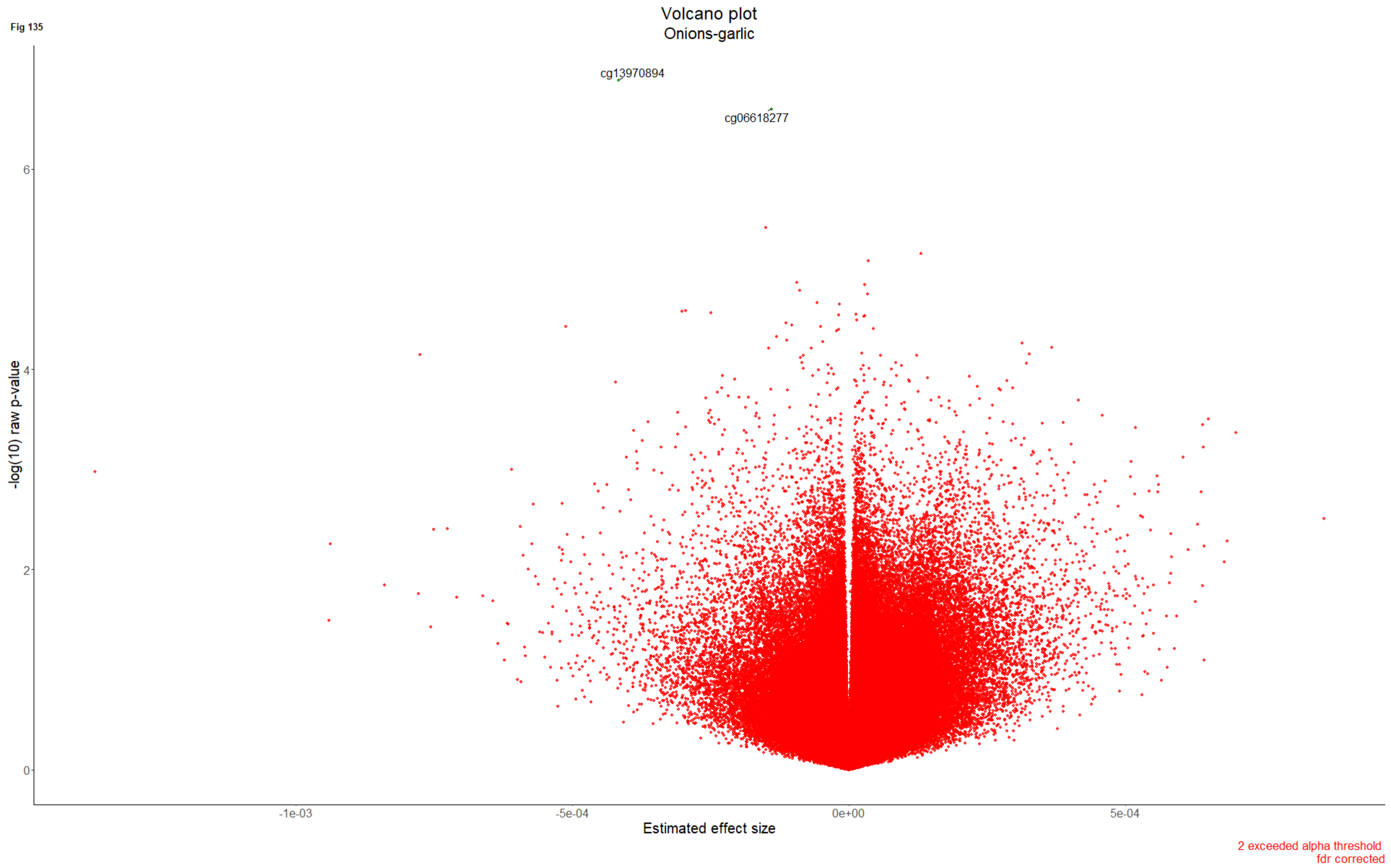
Volcano plot
Onions-garlic
cg13970894
cg06618277
-log(10) raw p-value
6
4
2
0
-1e-03
-5e-04
0e+00
5e-04
Estimated effect size
2 exceeded alpha threshold
fdr corrected

Fig 136

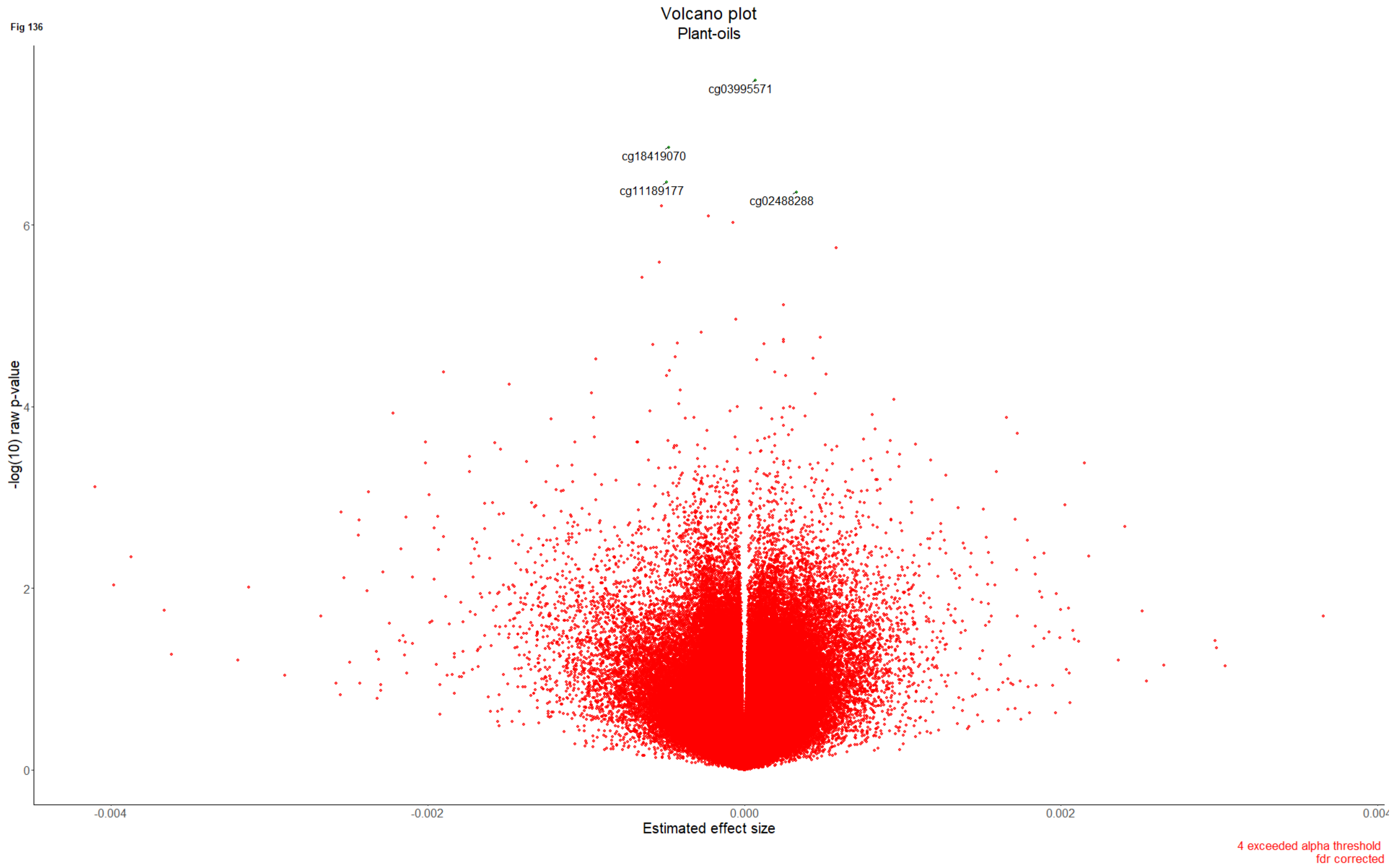

Volcano plot
Plant-oils
cg03995571
cg18419070
cg11189177
cg02488288
-log(10) raw p-value
0
2
4
6
-0.004
-0.002
0.000
0.002
Estimated effect size
4 exceeded alpha threshold
fdr corrected

Fig 137

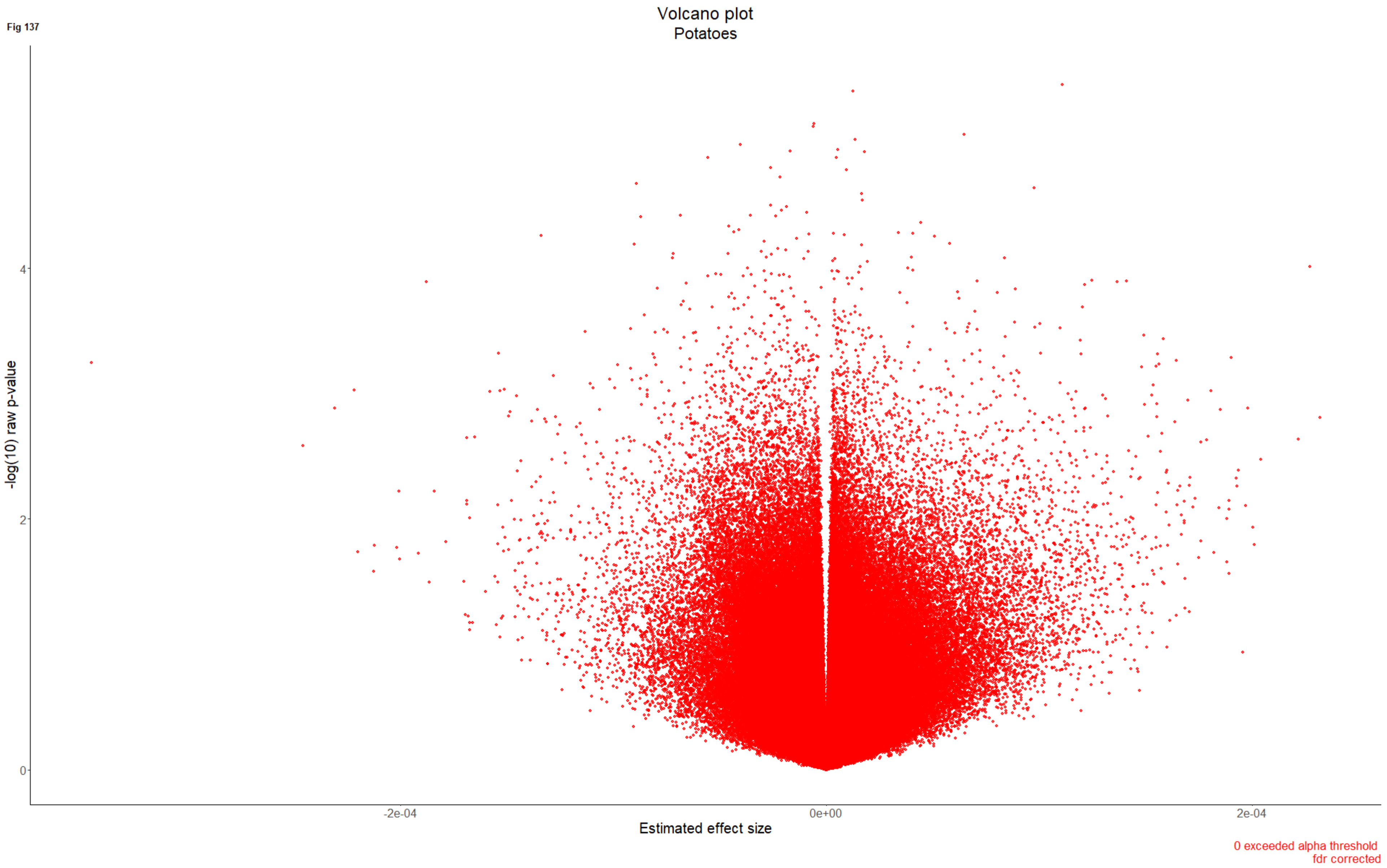
Volcano plot
Potatoes
-log(10) raw p-value
4
2
0
-2e-04
0e+00
2e-04
Estimated effect size
0 exceeded alpha threshold
fdr corrected

Fig 138

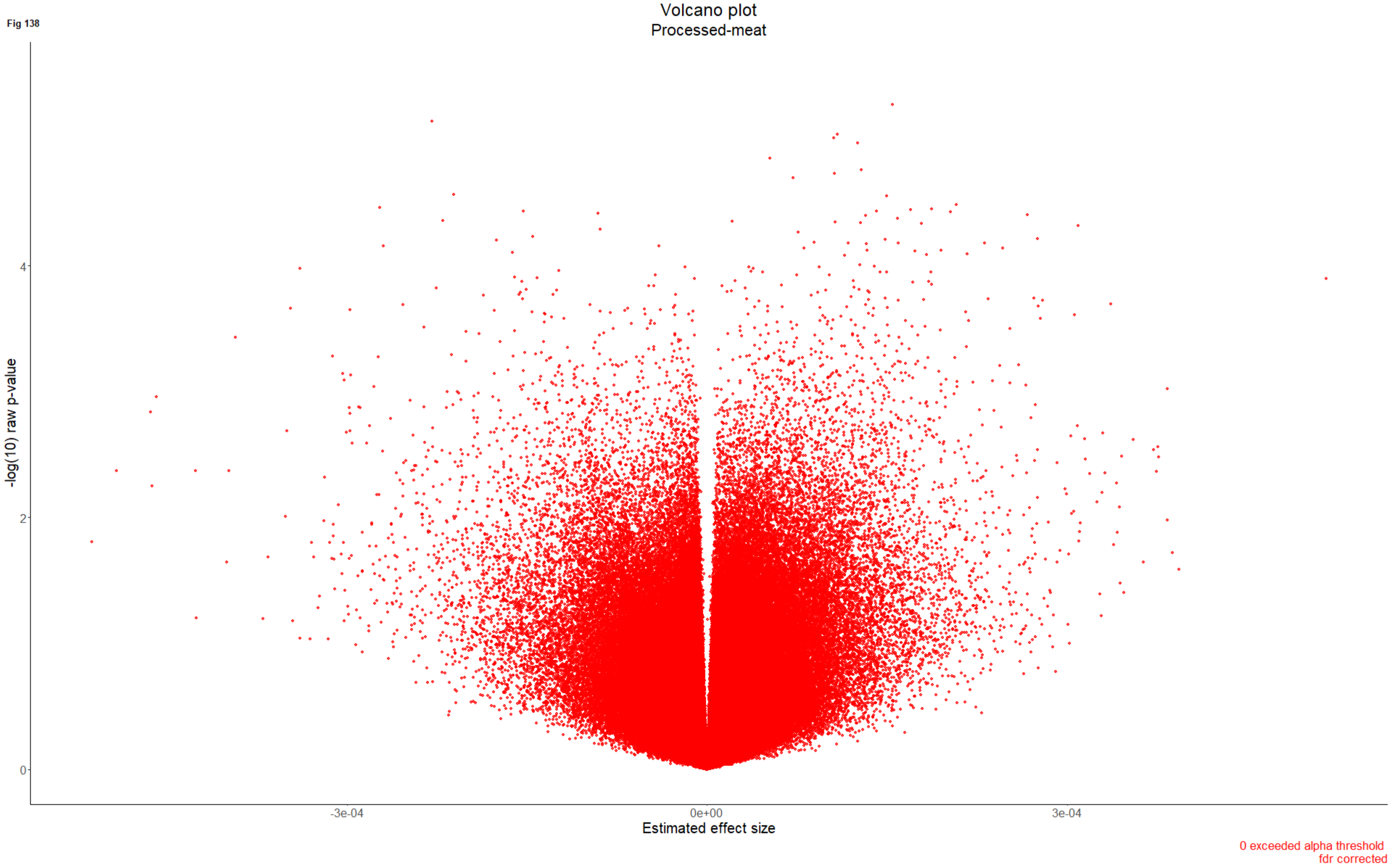

Volcano plot
Processed-meat
-log(10) raw p-value
4
2
0
-3e-04
0e+00
3e-04
Estimated effect size
0 exceeded alpha threshold
fdr corrected

Fig 139

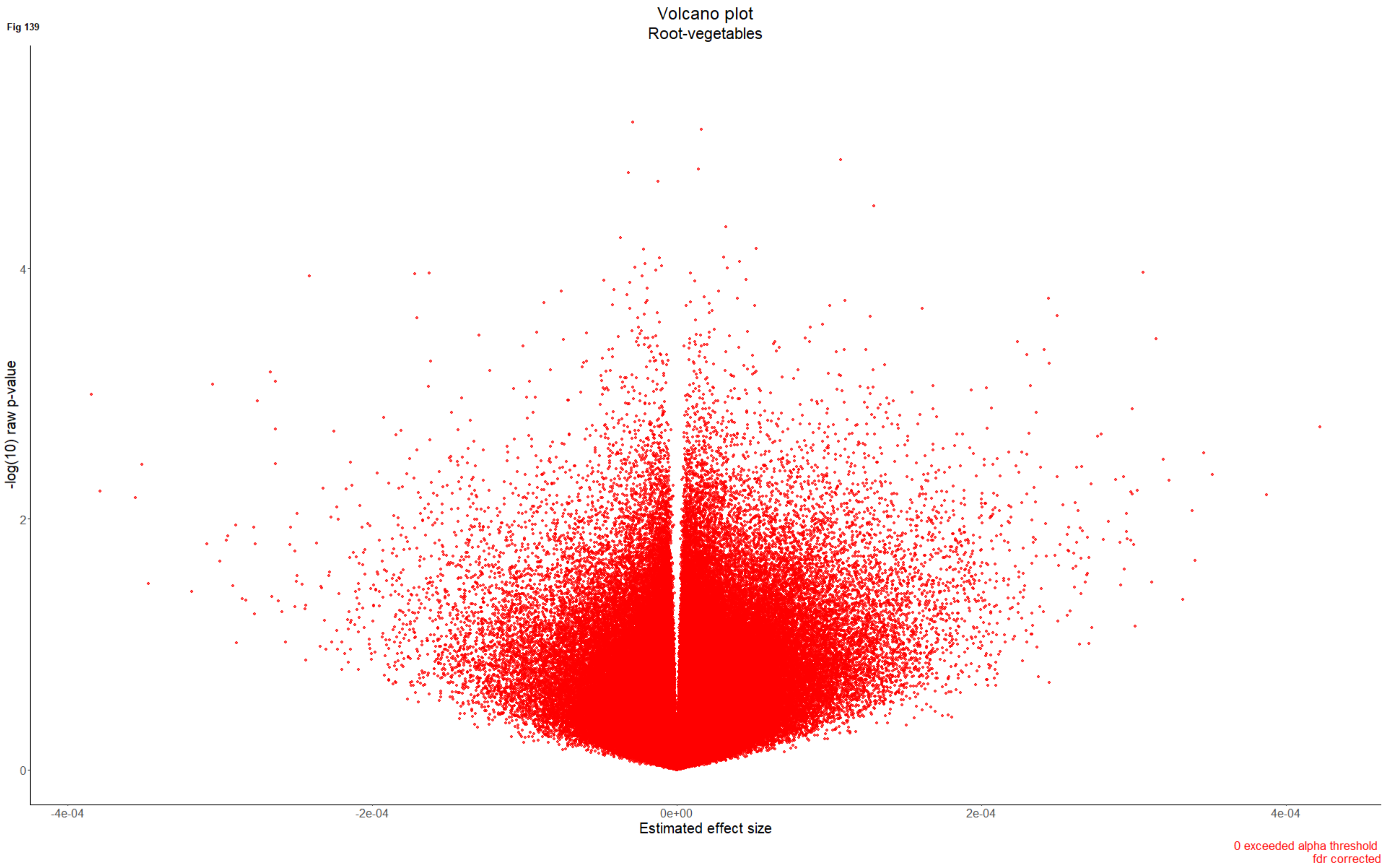
Volcano plot
Root-vegetables
-log(10) raw p-value
0
2
4
-4e-04
-2e-04
0e+00
2e-04
4e-04
Estimated effect size
0 exceeded alpha threshold
fdr corrected

Fig 140

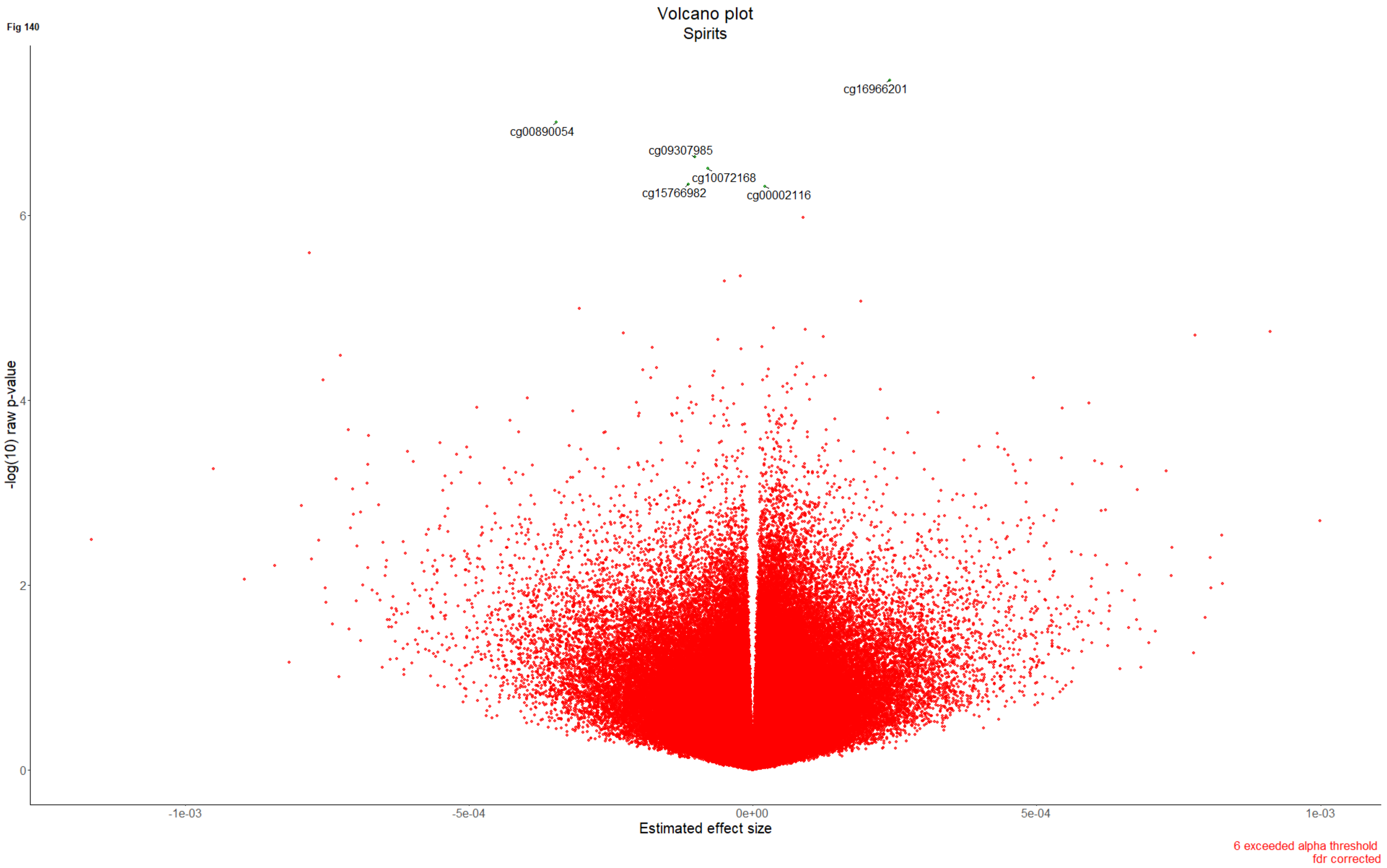

Volcano plot
Spirits
cg16966201
cg00890054
cg09307985
cg10072168
cg15766982
cg00002116
-log(10) raw p-value
0
2
4
6
-1e-03
-5e-04
0e+00
5e-04
1e-03
Estimated effect size
6 exceeded alpha threshold
fdr corrected

Fig 141

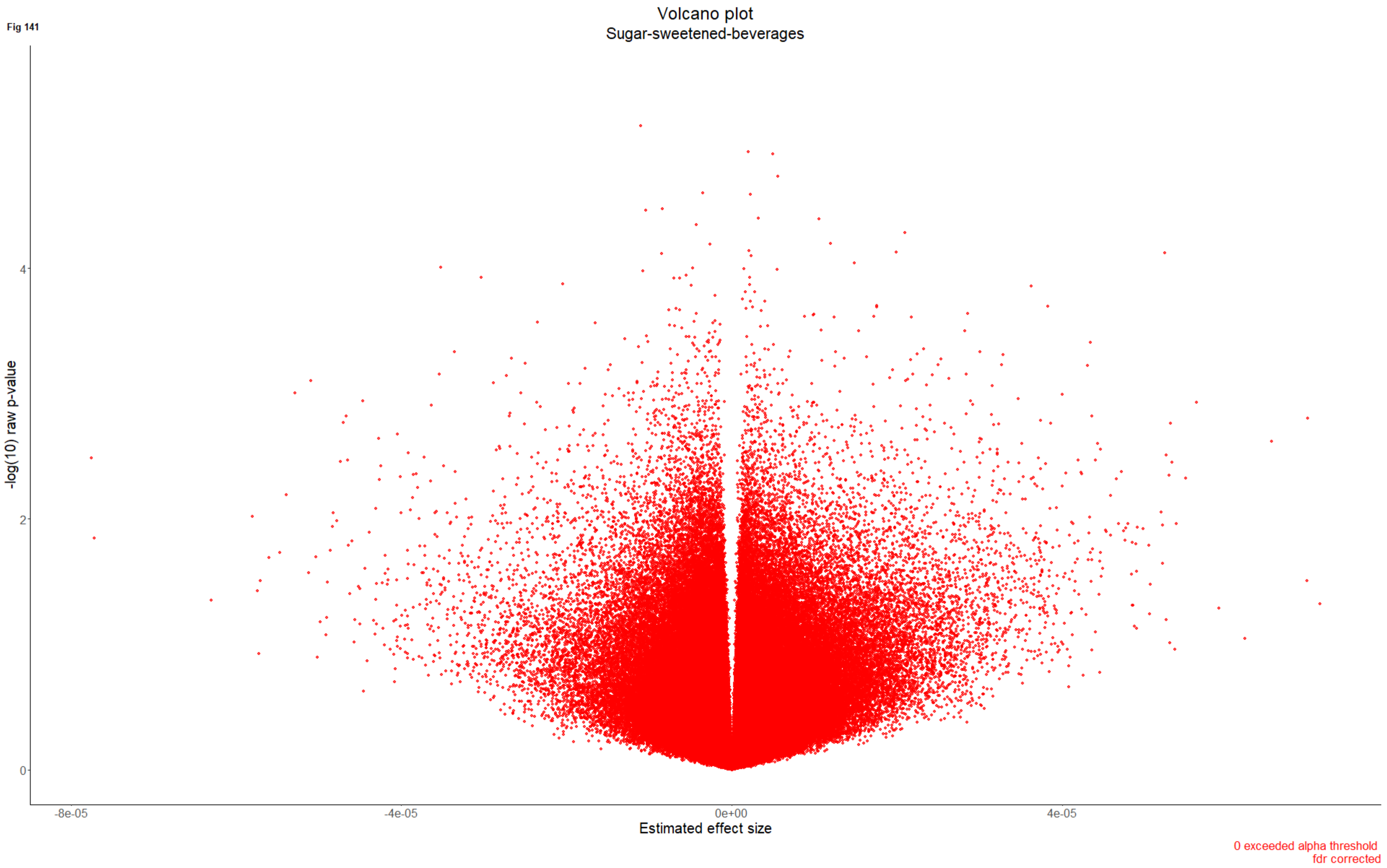
Volcano plot
Sugar-sweetened-beverages
-log(10) raw p-value
4
2
0
-8e-05
-4e-05
0e+00
4e-05
Estimated effect size
0 exceeded alpha threshold
fdr corrected

Fig 142

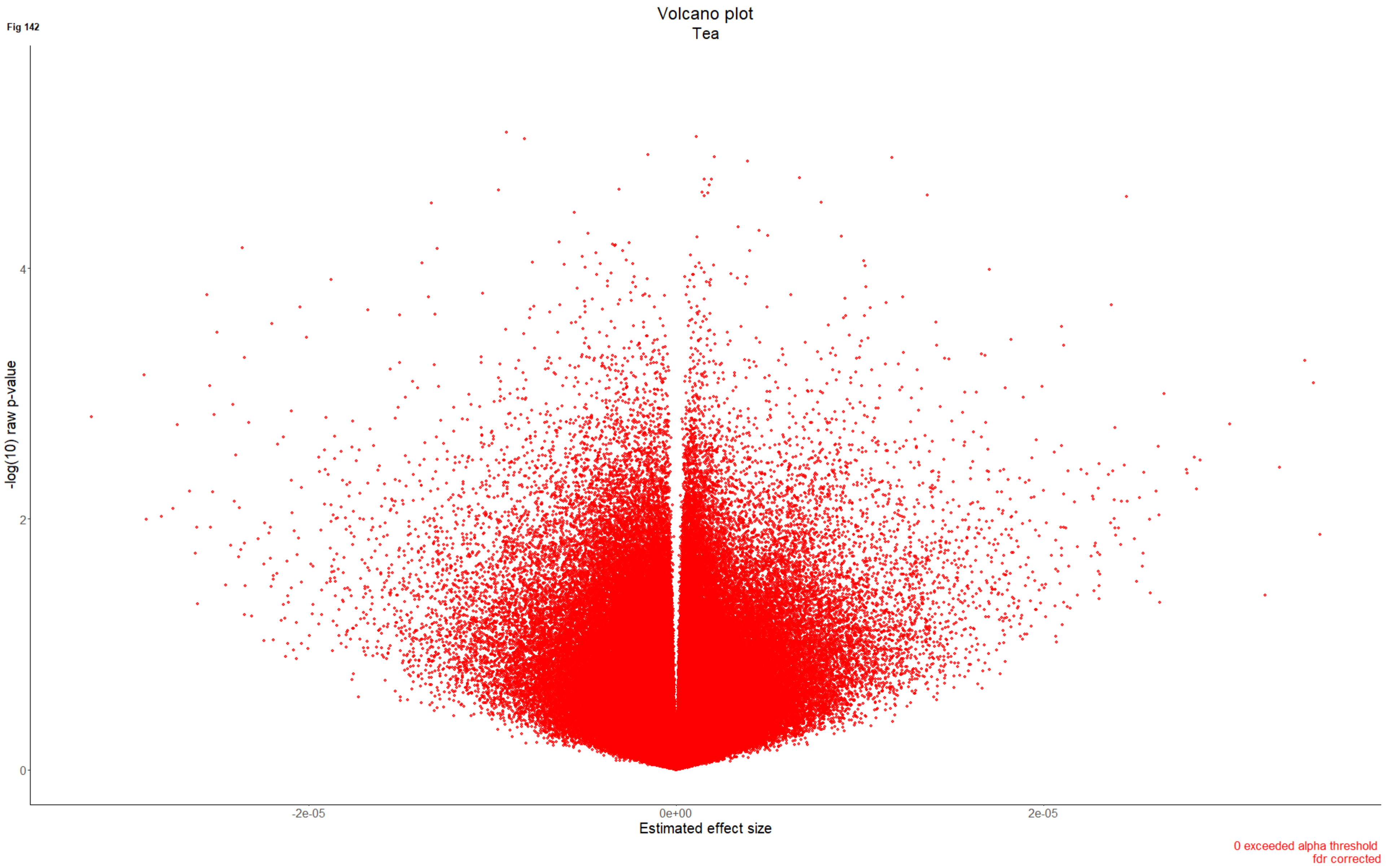
Volcano plot
Tea
-log(10) raw p-value
0
2
4
-2e-05
0e+00
2e-05
Estimated effect size
0 exceeded alpha threshold
fdr corrected

Fig 143

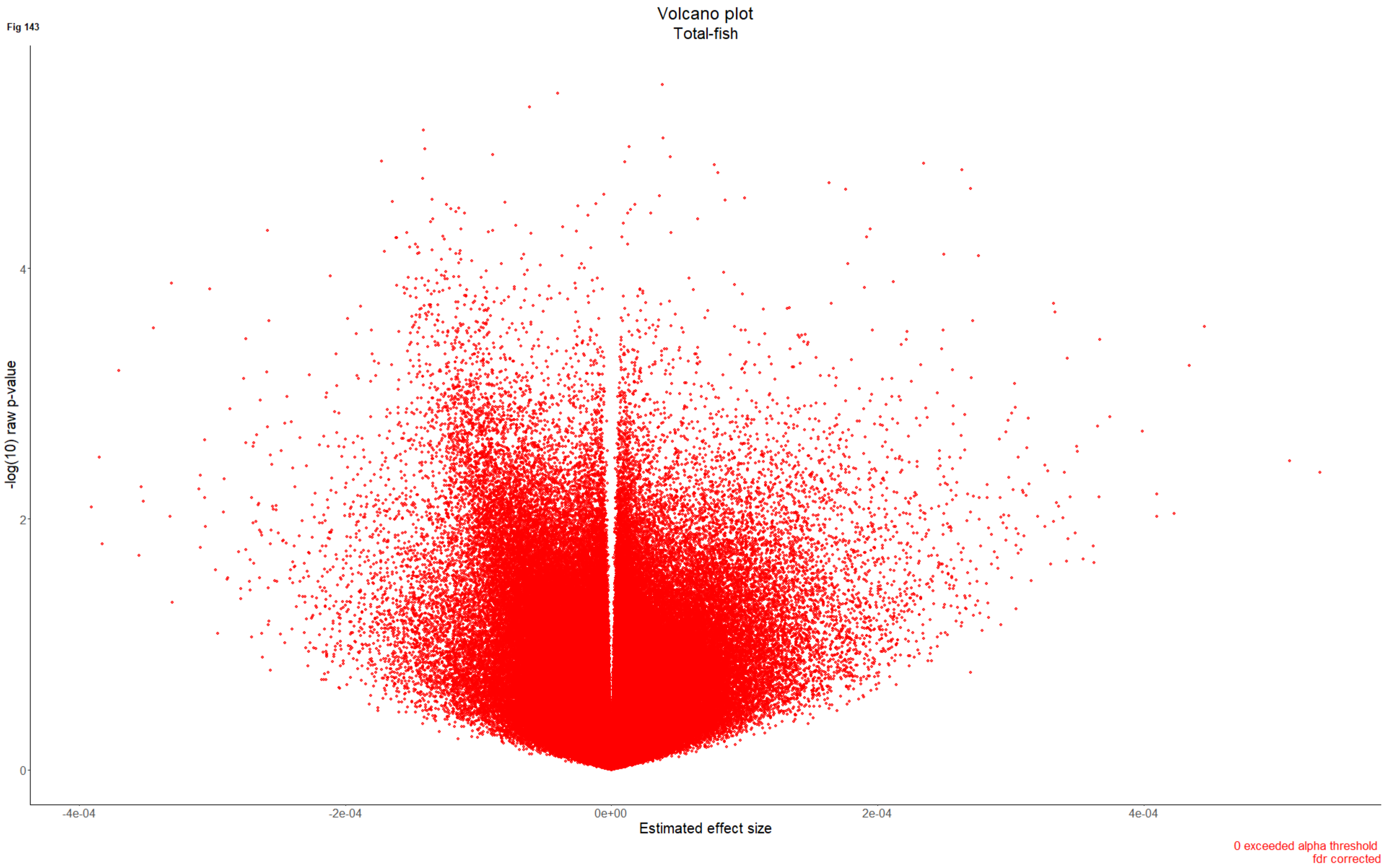
Volcano plot
Total-fish
-log(10) raw p-value
0
2
4
-4e-04
-2e-04
0e+00
2e-04
4e-04
Estimated effect size
0 exceeded alpha threshold
fdr corrected

Fig 144

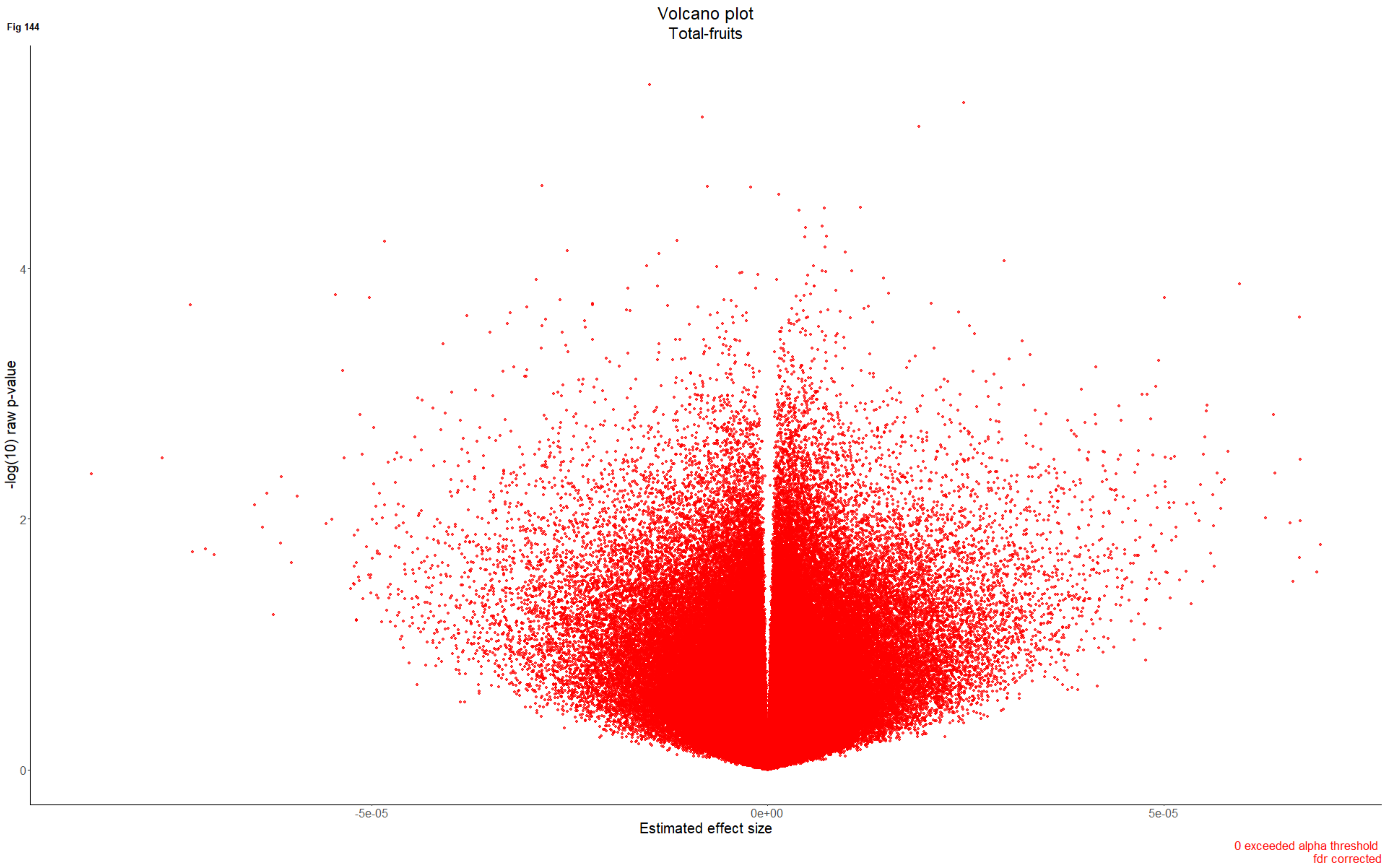
Volcano plot
Total-fruits
-log(10) raw p-value
4
2
0
-5e-05
0e+00
5e-05
Estimated effect size
0 exceeded alpha threshold
fdr corrected

Fig 145

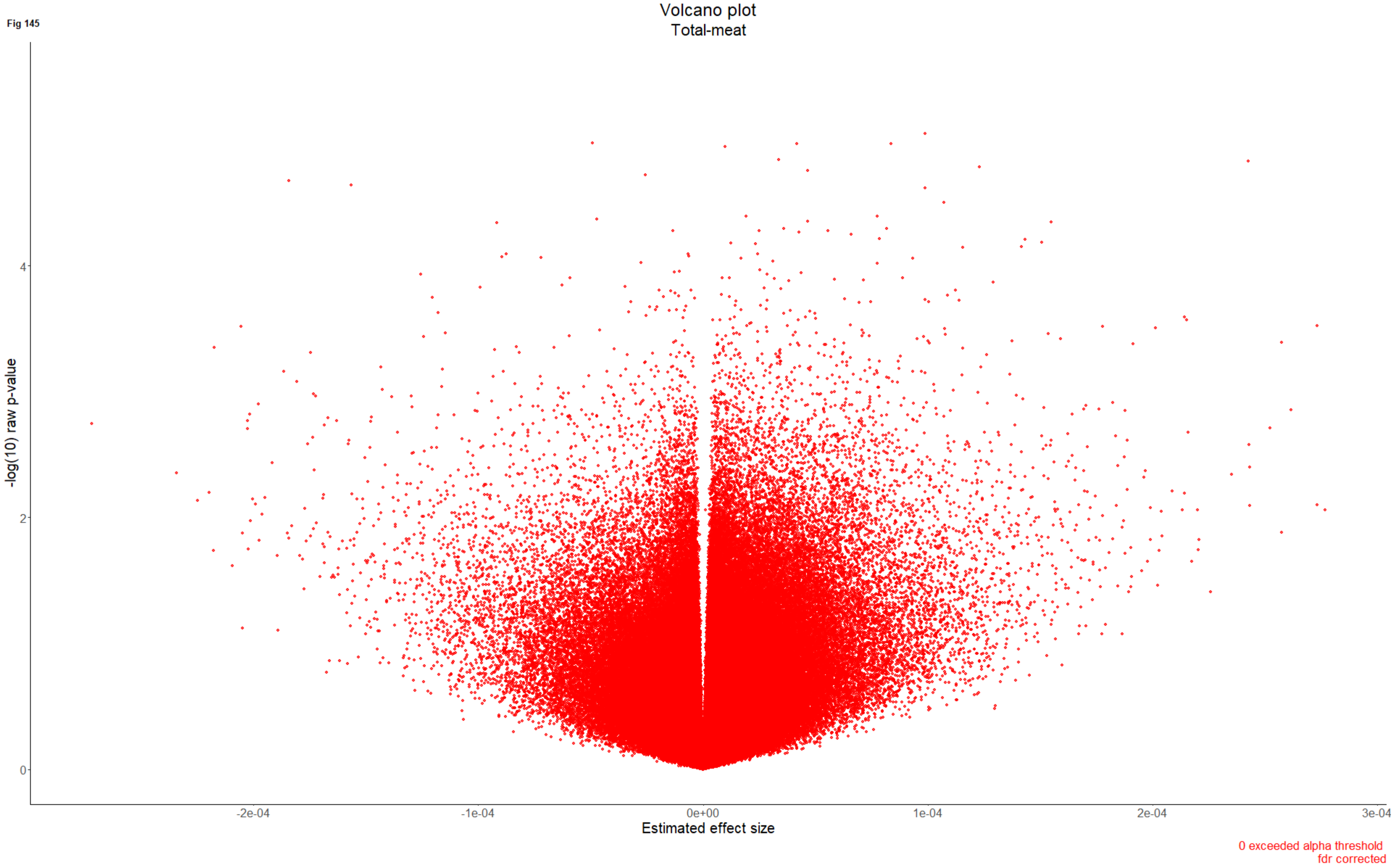

Volcano plot
Total-meat
-log(10) raw p-value
4
2
0
-2e-04
-1e-04
0e+00
1e-04
2e-04
3e-04
Estimated effect size
0 exceeded alpha threshold
fdr corrected

Fig 146

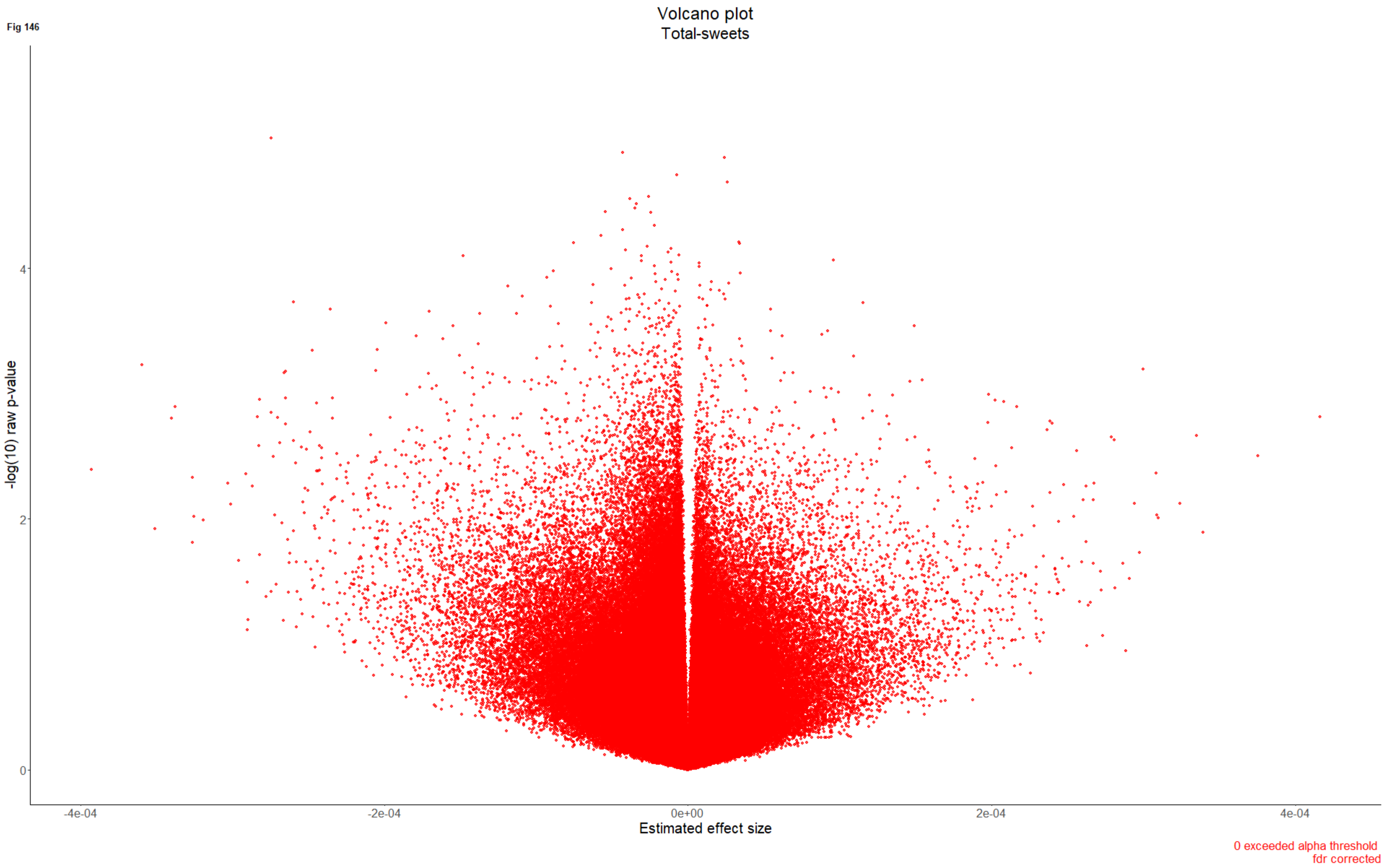
Volcano plot
Total-sweets
-log(10) raw p-value
4
2
0
-4e-04
-2e-04
0e+00
2e-04
4e-04
Estimated effect size
0 exceeded alpha threshold
fdr corrected

Fig 147

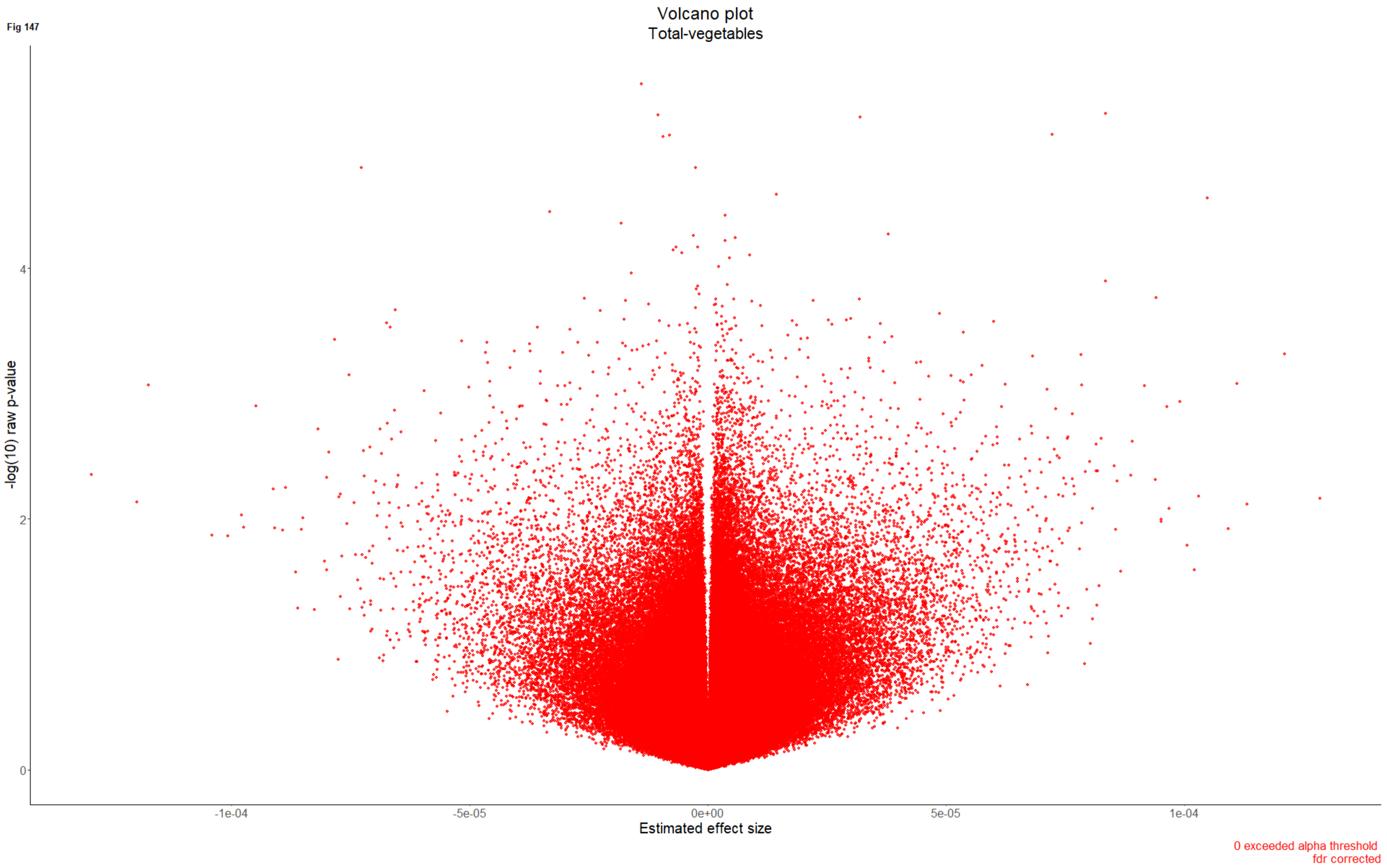
Volcano plot
Total-vegetables
-log(10) raw p-value
4
2
0
-1e-04
-5e-05
0e+00
5e-05
1e-04
Estimated effect size
0 exceeded alpha threshold
fdr corrected

Fig 148

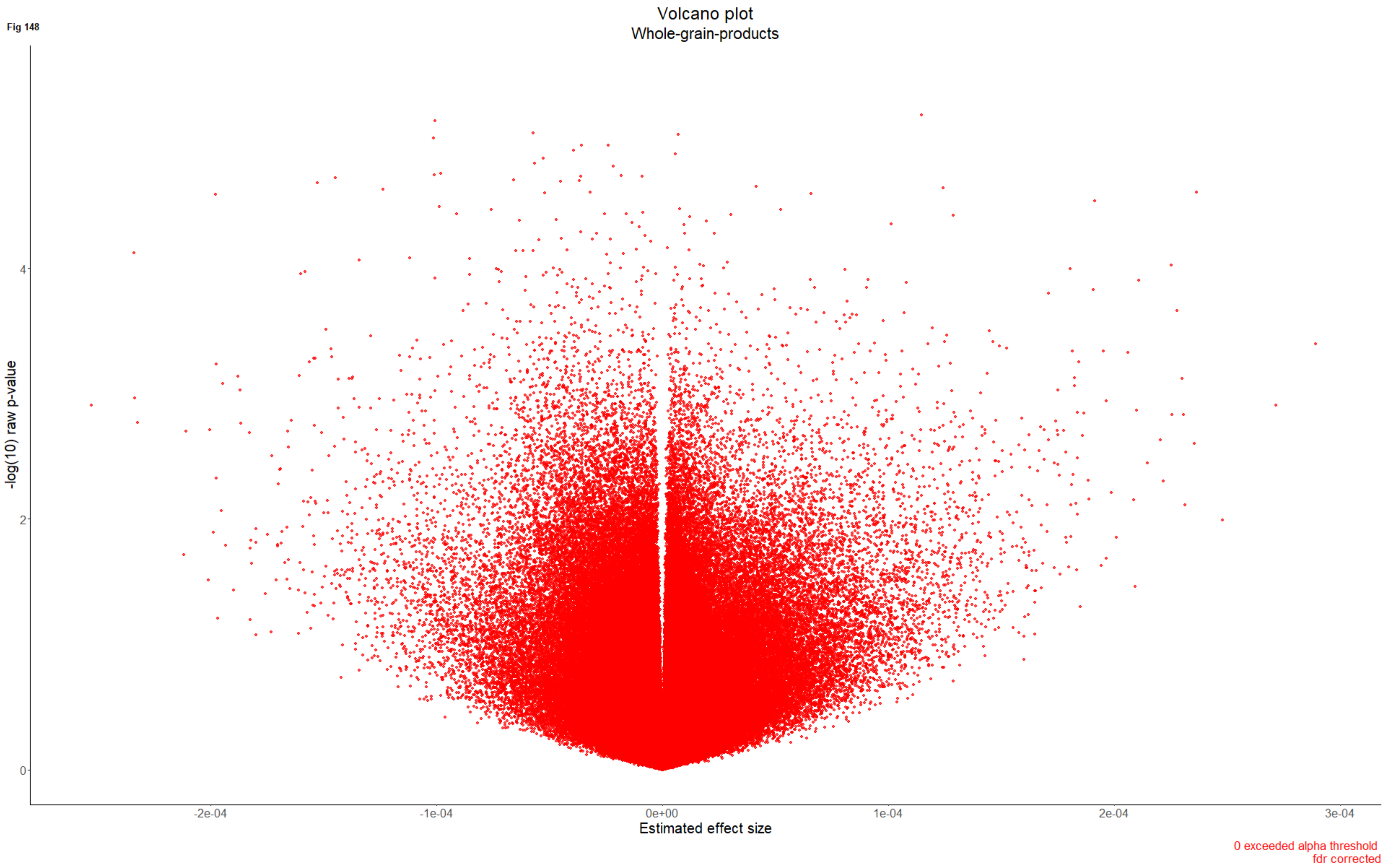
Volcano plot
Whole-grain-products
-log(10) raw p-value
4
2
0
-2e-04
-1e-04
0e+00
1e-04
2e-04
3e-04
Estimated effect size
0 exceeded alpha threshold
fdr corrected

Fig 149

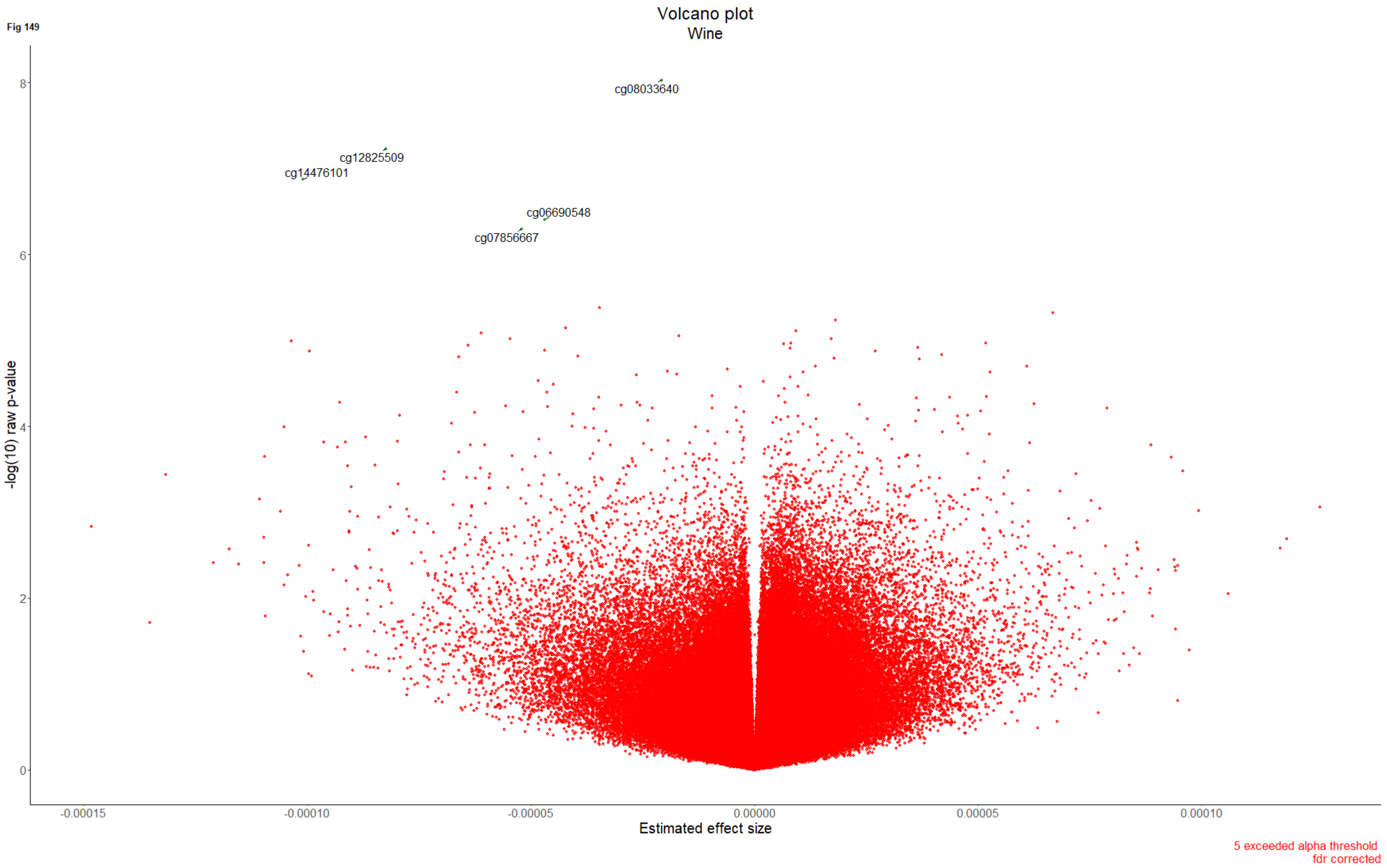
Volcano plot
Wine
cg08033640
cg12825509
cg14476101
cg06690548
cg07856667
-log(10) raw p-value
8
6
4
2
0
-0.00015
-0.00010
-0.00005
0.00000
0.00005
0.00010
Estimated effect size
5 exceeded alpha threshold
fdr corrected

Fig 150

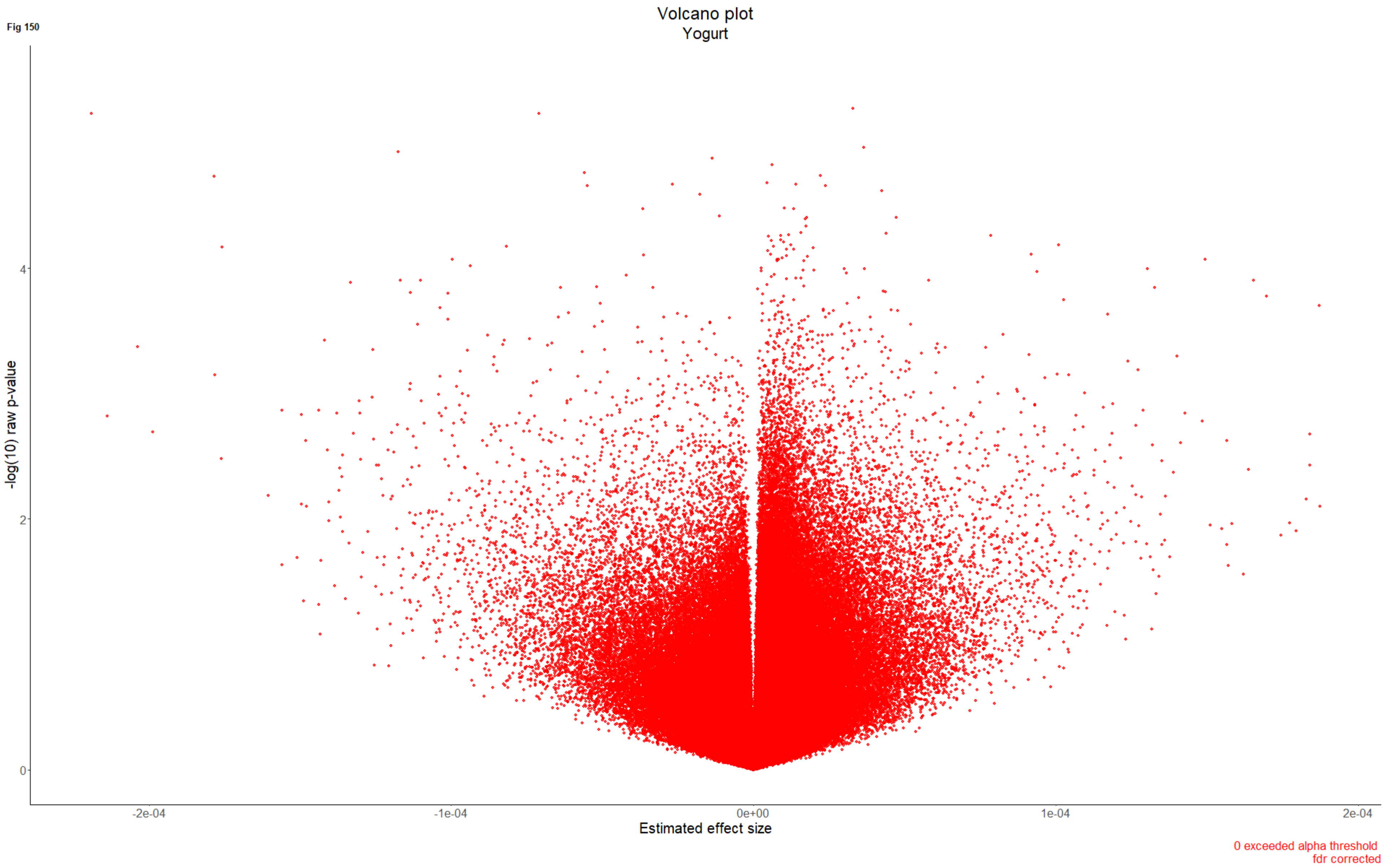
Volcano plot
Yogurt
-log(10) raw p-value
0
2
4
-2e-04
-1e-04
0e+00
1e-04
2e-04
Estimated effect size
0 exceeded alpha threshold
fdr corrected
